# In vitro and in silico neuroprotective evaluation of new biotransformation metabolites of (-)-*α*-bisabolol

**DOI:** 10.1038/s41598-025-11694-4

**Published:** 2025-07-26

**Authors:** Reham Mansour, Ramadan A. Eldomany, Amira Mira, Mohamed A. Sabry, Saleh H. El-sharkawy, Amal F. Soliman

**Affiliations:** 1 Department of Pharmacognosy, Faculty of Pharmacy, Kafr El-Sheikh University, Kafr El-Sheikh, 33516 Egypt; 2 Department of Microbiology and Immunology, Faculty of Pharmacy, Kafr El-Sheikh University, Kafr El-Sheikh, 33156 Egypt; 3 Department of Pharmacognosy, Faculty of Pharmacy, Mansoura University, Mansoura, 35516 Egypt; 4 Department of Medicinal Chemistry, Faculty of Pharmacy, Mansoura National University, Gamasa, 7731168 Egypt; 5 Department of Medicinal Chemistry, Faculty of Pharmacy, Mansoura University, Mansoura, 35516 Egypt; 6 Department of Basic Sciences, Faculty of Physiotherapy, Rashid University, Rashid, 22754 Egypt; 7 Pharmacognosy Department, Faculty of Pharmacy, Mansoura National University, Gamasa, 7731168 Egypt

**Keywords:** Bisabolol, Biotransformation, Hamanasic acid A, COX inhibitors, Cholinesterase inhibitors, Neuroprotective, Drug discovery, Molecular biology

## Abstract

Biotransformation of (-)-*α*-bisabolol **(1)** was investigated by screening twenty-two fungal strains in an effort to produce new more polar and potentially bioactive metabolites. Three fungi were selected for scale-up biotransformation: *Cordyceps sinensis*, *Alternaria alternata* and *Aspergillus flavus.* Five metabolites were isolated: 10*β*,11-dihydroxy-*α*-bisabolol **(2)**, Hamanasic acid A **(3)**, 2,3-dihydro-*α*-bisabolol **(4)**, 7-dehydroxy-10,11-epoxy-3-methylcarboxy-*α*-bisabolol **(5**) and 10*β*,11,15-trihydroxy-*α*-bisabolol **(6),** with metabolites **4**, **5**, and **6** being newly identified. Structural elucidation was performed using spectroscopic methods. *α*-bisabolol and its metabolites were evaluated for their cyclooxygenase (COX) and acetylcholinesterase (AChE) inhibitory activities, as well as neuroprotective effects against H_2_O_2_ and A*β*_1-42_-induced toxicity in SH-SY5Y cells. In vitro results showed that metabolite **5** exhibited the strongest COX-2 inhibition (IC_50_ = 2.508 µM), while **2** showed AChE inhibition (IC_50_ = 12.94 µM), These outcomes were more confirmed by molecular docking. Metabolites **6** and **2** demonstrated superior neuroprotective effects against H_2_O_2_ and A*β*_1-42_-induced toxicity compared to *α*-bisabolol. Importantly, metabolite **2** showed pronounced AChE inhibitory activity alongside favorable ADMET attributes. These findings suggest that *α*-bisabolol and its metabolite **2** are potential candidates for the modulation of neurodegenerative diseases involving inflammation, neurotoxicity, or cholinergic dysfunction. Further in vivo investigations are mandatory to ensure the study outcomes.

**Supplementary Information:**

The online version contains supplementary material available at 10.1038/s41598-025-11694-4.

## Introduction

(-)-*α*-bisabolol (levomenol) is a naturally occurring oily sesquiterpene alcohol with limited solubility in water and a distinct woody-floral scent^1^. It is derived from the essential oils of many edible and decorative plants such as German chamomile *(Matricaria chamomilla*, F:Asteraceae), *Eremanthus erythropappus, Smyrniopsis aucheri, Vanillosmopsis* species, *Salvia runcinata*, *S. stenophylla* (Lamiaceae)^2^ and the marine seaweed *Padina gymnospora*^3^.

(-)-*α*-bisabolol is an important active ingredient in cosmetic formulations, attributed to its anti-inflammatory, antimicrobial and skin-soothing properties. Additionally, it is readily absorbed through the skin without causing photosensitivity or skin irritation following application^4,5^. It also fulfills several pharmacological activities including: anticancer^6^, anti-nociceptive^7^, neuroprotective^8^, peripheral nervous blocking^9^, anti-leishmanial^10^ and insecticidal activities^11^. The Food and Drug Administration (FDA) has categorized (-)-*α*-bisabolol as ‘Generally Regarded As Safe GRAS’, because of its low toxicity as well as its antimicrobial properties^12^.

The neuroprotective action of (-)-*α*-bisabolol was evaluated by testing the following: antioxidant, anti-aggregation, anti-apoptotic activities as well as acetylcholinesterase (AChE) inhibitory potential using amyloid *β*-protein (A*β*) induced neurotoxicity in Neuro-2a cells. Results revealed that cells pre-treated with (-)-*α*-bisabolol showed improved potential to scavenge reactive oxygen and nitrogen species, restored mitochondrial membrane potential (MMP) loss and prevented induced apoptosis. Furthermore, AChE was reduced significantly in cells pretreated with *α*-bisabolol^13^. In another study, *α*-bisabolol was reported to prevent the formation of A*β* oligomers and to disaggregate already formed mature fibrils using(A*β*_23-35_) induced neurotoxicity model^8^. The reported anti-inflammatory activity of *α*-bisabolol indicated a reduction in the expression of cyclooxygenase-2 enzyme (COX-2), tumor necrosis factor-*α* (TNF-*α*), prostaglandin E2 (PG E2), nitric oxide, nitrite, interleukin-6 (IL-6), interleukin-1*β* (IL-1*β*), macrophage inflammatory protein-2 (MIP-2), chemokine (KC) and reducing recruited leukocytes to the site of injury^14–17^.

(-)-*α*- bisabolol is a lipophilic compound which affects its solubility in biological fluids, bioavailability, physicochemical and pharmacokinetic properties. To overcome this problem, biotransformation techniques were adopted using different fungal strains as biological catalysts, with (-)-*α*-bisabolol serving as the substrate, to generate more polar derivatives under mild conditions. One of the key characteristics of biotransformation is the preservation of the original carbon skeleton of the substrate^18^. These processes often eliminate the need to protect other reactive functional groups. Furthermore, biotransformation offers high stereo-selectivity and regio-selectivity, enabling complex modifications in a single step that would typically require multiple stages in chemical synthesis. It also allows for the functionalization of remote, non-activated positions and is considered both environmentally friendly and cost-effective due to reduced waste production and energy consumption^19^.

According to previous documented biotransformation studies of (-)-*α*-bisabolol; a suspension culture of *Bipolaris Sorokiniana* was able to transform (-)-*α*-bisabolol into *α* –bisabolol oxide B and 4,5- bisabolol epoxide^20^. In another study, *Glomeralla Cingulata* was able to transform *α* –bisabolol into 3,4- dihydroxy -bisabolol oxide B. Also, *Aspergillus Niger* was reported to transform *α* –bisabolol into bisabolol oxide B, *α*-tetrahydrobisabolene-2,5,6-triol and 3,4-dihydroxy bisabolol oxide B^21^. *Absidia coerulea* was able to transform (-)-*α*-bisabolol into (1*R*,5*R*,7*S*)-5,hydroxyl-*α*-bisabolol, (1*R*,5*S*,7*S*)‐5‐hydroxy‐*α*‐bisabolol, (1*R*,5*R*,7*S*,10*S*)‐5‐hydroxy‐bisabolol oxide B, (1*R*,7*S*,10*S*)‐1‐hydroxybisabolol oxide B , 12‐hydroxy‐*α*‐bisabolol, (1*S*,3*R*,4*S*,7*S*)‐3,4‐dihydroxy‐ *α*‐bisabolol and (1*S,*3*S,*4*S,*7*S*)‐3,4‐dihydroxy‐*α*‐bisabolol^22^.

This study aimed to use biotransformation techniques to convert (-)-*α*-bisabolol into more polar and more biologically active compounds, with the goal of discovering new candidates with potential therapeutic effects on neurodegenerative disorders.

## Results and discussion

**Fig. 1:**
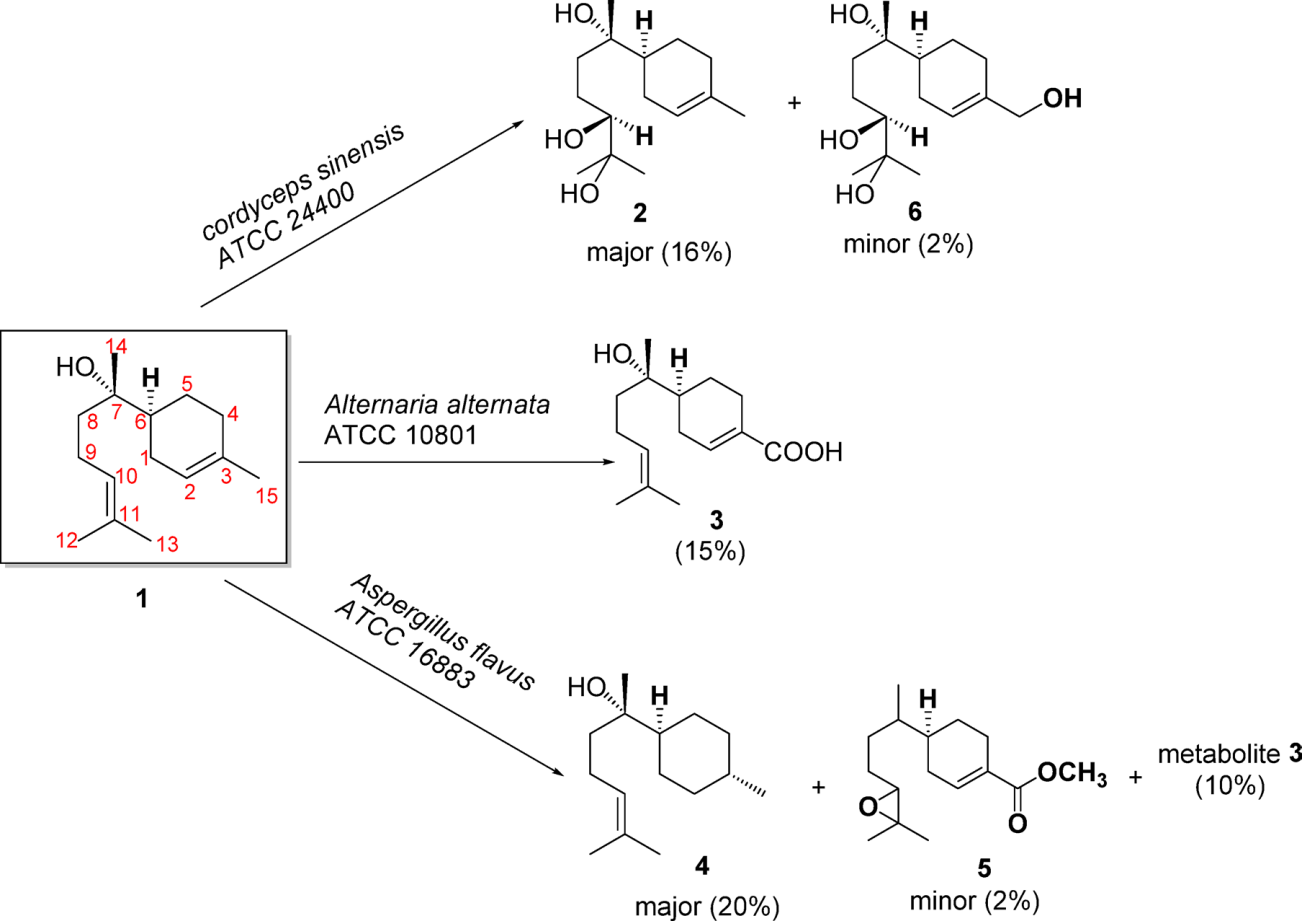
Schematic presentation of the bioconversion of *α*-bisabolol (**1**) to its metabolites using three different fungal strains.

A total of 22 microbial cultures were used in the initial screening of (-)-*α*-bisabolol **(1)**. Five biotransformation metabolites **(2–6)** were produced by *Cordyceps sinensis* ATCC 24400 (**2** and **6**), *Alternaria alternata* ATCC 10801 (**3**) and *Aspergillus flavus* ATCC 16883 (**3**, **4** and **5**) (Fig. 1). All the metabolites were chemically identified on the basis of their Mass, IR, ^1^H-NMR, APT and 2D-NMR spectroscopic data. Metabolites **2** and **3** were previously isolated from natural sources^23,24^. This is the first report describing the isolation and identification of metabolites **4–6** utilizing bio-catalytic transformation.

### Spectral data of (-)-*α*-bisabolol

(-)-*α*-bisabolol (**1)** is a colorless viscous volatile oil. Its molecular formula is **C**_**15**_**H**_**26**_**O** with a molecular weight 222 Da. Its mass spectrum showed a peak at **m/z 204,** corresponding to **[M-H**_**2**_**O]**^**+**^. Its IR spectrum indicated the presence of hydroxyl group (3442.5 cm^-1^), as well as a double bond (1637, 772.2 cm^-1^). [*α*]_D_ – 55.7 (levorotatory) which confirms its configuration (6*S*, 7*S* -*α*- bisabolol)**.** ^1^H-NMR spectrum (see Supplementary Figs. S1 and S2 online) showed three signals assigned to methine hydrogens, two of them were directly attached to sp^2^ carbons (H-2 at δ 5.32, m; H-10 at δ 5.08, t) and the third signal (at δ 1.55 – 1.48, m) corresponding to H-6. There were four signals corresponding to four methyl groups δ_H_ 1.63 (H-12, 3H, S), 1.57 (H-13, 3H, S), 1.05 (H-14, 3H, S), 1.60 (H-15, 3H, S). Five methylene groups were also observed (H-1, m, 1.85–1.73 H*α* and 2.00 to 1.95, H*β*; H-5, m, 1.29–1.16, H*α* and 1.90–1.84, H*β*, H-4 at δ 1.94–1.93, H-8 at δ 1.47–1.42, and H-9 at δ 2.04–1.97. APT spectrum (see Supplementary Fig. S3 online) showed three signals consistent with methine carbons (C-2 at δ 120.5, C-6 at δ 43.00 and C-10 at δ 124.6). It also showed three signals relating to three quaternary carbons, two of them were olefinic C-3 (δ 134.0), C-11 (δ 131.5) and the third was oxygenated carbon C-7 (74.24). Four signals corresponding to methyl carbons were observed: C-12 (δ 25.6), C-13 (δ 17. 6), C-14 (δ 23.1) and C-15 (δ 23.3). Five signals matching with methylene carbons can also be seen: C-1 (δ 26.9), C-4 (δ 31.0), C-5 (δ 23.0), C-8 (δ 40.1), C-9 (δ 22.0) (see Supplementary Tables S1 and S2 online). ^1^H-NMR, APT, IR and [*α*]_D_ of purchased (-)-*α*-bisabolol were consistent with previously reported data of (-)-*α*-bisabolol^25^.

### Microbial transformation using *Cordyceps sinensis* ATCC 24400

Incubation of compound **1** with *Cordyceps sinensis* (ATCC 24400) for 14 days resulted in the formation of two metabolites (**2** and **6**), with yields of 16% and 2%, respectively, without any optimization. The molecular formula of **2** was determined to be C_15_H_28_O_3_ based on ESI–MS analysis (see Supplementary Fig. S13 online), which showed a deprotonated ion peak at m/z 255.1, [M-H]^-^ (calcd. 255.3), indicating a mass increase of 34 Da relative to the parent compound (**1**), suggesting addition of two hydroxyl groups. NMR spectra and [*α*]_D_ − 48.7 value of metabolite **2** were consistent with those previously reported for the natural secondary metabolite, 5,6-dihydroxybisabolol, which was isolated from the filamentous fungus *Bionectria* sp. (MSX 47401)^23^. This study reports, for the first time, the formation of this metabolite (**2**) via microbial biotransformation of (-)-*α*-bisabolol.

Detailed NMR analysis (see Supplementary Tables S1 and S2 online) revealed a high similarity between **2** and *α*-bisabolol. However, the ^1^H-NMR spectrum of **2** (see Supplementary Figs. S4, S5 and S6 online) showed disappearance of the olefinic proton signal at δ_H_ 5.08 (H-10), replaced by a signal at δ_H_ 3.31 (t, 1H), corresponding to an oxygenated methine proton. Additionally, APT spectrum (see Supplementary Fig. S7 online) showed disappearance of two olefinic carbon signals at δ_C_ 124.6 (CH, C-10) and δ 131.5 (C, C-11), with the appearance of two oxidized carbon signals at δ_C_ 79.1(CH) and 73.3(C). These findings suggest that the C-10(11) double bond was oxidized to form a vicinal diol. NOESY spectrum was used to deduce the stereochemistry of the hydroxyl group at C-10, as a correlation between both H-10 and H-6*α* was detected (see Supplementary Fig. S12 online). This correlation supports the assignment of the hydroxyl group at C-10 as *β*-oriented, suggesting the structure of **2** was 10*β*, 11-dihydroxy- *α* – bisabolol. The HMBC ^2^*J* correlation of H-15 (δ 1.58) with C-3 (δ 134.36), and ^3^*J* correlation of H-15 (δ 1.58) with C-2 (δ 120.5) and C-4 (δ 30.9) confirmed the position of the double bond between C-2 and C-3. Furthermore, HMBC ^2^*J* correlation of H-12 (δ 1.14) and H-13 (δ 1.09) with C-11 (δ 71.6) and ^3^*J* correlation of H-13 (δ 1.09) with C-10 (δ 79.12) confirmed the position of hydroxyl groups at C-10 and C-11 (see supplementary Figs. S10 and S11 online).

A proposed biosynthetic pathway of **2** (see supplementary Fig. S51 online) suggests that the transformation involves enzymatic epoxidation of the C-10(11) double bond by a monooxygenase enzyme^26,27^, followed by hydrolytic cleavage of the resulting epoxide by a hydrolase enzyme^28^, yielding the diol 10*β*,11-dihydroxy-*α*-bisabolol. Similar epoxidation–hydrolysis transformation reaction was previously reported using *Glomerella cingulata* transforming *β*-silenene to 1,11,13-trihydroxy-*β*-selinene^29^.

Metabolite **6** was obtained as colorless viscous oil in small amounts (5 mg). The molecular formula of 6 was established as C_15_H_28_O_4_ by HR-ESI-MS spectrum (see supplementary Fig. S49 online) which displayed a peak at *m/z* 156.05156 ([M + K + H]^++^, calcd.156.125). This represents an increase of 16 Da over metabolite **2**, indicating the addition of one oxygen atom.

NMR spectral analysis of **6** was similar to that of metabolite **2** (see supplementary Tables S1 and S2 online) but ^1^H-NMR of **6** (see supplementary Figs. S46 and S47 online) revealed a downfield shift of the olefinic proton H-2 by 0.34 ppm. Furthermore, the methyl signal at **δ**_**H**_ 1.58 (s, 3H, H-15) disappeared, replaced by a new signal resonating at δ_H_ 3.65 (s, 2H), suggesting oxidation of the methyl group at C-15 to the corresponding primary alcohol. This oxidation was more confirmed by APT spectrum (see Supplementary Fig. S48 online) which showed the loss of the methyl carbon signal at δ_C_ 23.3 (CH_3_, C-15) and appearance of a new oxidized methylene signal at δ_C_ 63.7 (CH_2_), Additionally, the olefinic carbon C-2 was de-shielded, shifting downfield from δ_C_ 120.5 to 124.03 ppm. Given the structural similarity between metabolites **2** and **6**, and their co-occurrence in the same fermentation extract, it is plausible that metabolite **2** acts as a biosynthetic intermediate, which undergoes further oxidation to yield metabolite **6**. Consequently, the structure of **6** was assigned as 10*β*,11,15-trihydroxy-*α*-bisabolol. The proposed biosynthetic pathway for **6** mirrors that of metabolite **2**, initially involving epoxidation and hydrolysis of the C-10(11) double bond. Additionally, an allylic hydroxylation step introduces the hydroxyl group at C-15, converting the terminal methyl into a primary alcohol. This allylic hydroxylation reaction has previously been observed in the formation of 15-hydroxybisabolol oxide A glycoside^30^ and is supposed to be catalyzed by cytochrome p450 monooxygenase^38^.

### Microbial transformation using *Alternaria alternata* ATCC 10801

Biotransformation of **1** with *Alternaria alternata* (ATCC 10801) produced one metabolite (**3**) after 7 days’ incubation (15% yield without optimization). Its ^1^H, APT ^13^C-NMR spectra (in CDCL_3_) (see Supplementary Figs. S15, S16, S17 and S18 online) and [*α*]_D_ -63 were consistent with those for (-)-Hamanasic acid A, which was previously reported to be isolated from the aqueous extract of *Flourensia campestris* (Asteraceae) dry aerial parts^24^. This is the first report documenting the isolation of (-)-Hamanasic acid A from (-)-*α*-bisabolol biotransformation.

The molecular formula of **3** was recognized as C_15_H_24_O_3_ using EI-MS spectrum that displayed a peak at m/z 234 which is compatible with the loss of water from the parent ion [M-H_2_O]^+^ (see Supplementary Fig. S23 online) The structure was more confirmed by the HMBC ^2^*J* and ^3^*J* correlations of H-2 (δ 7.01) with C-1(δ 24.9), C-3(δ 129.95) and C-15(δ 171.6), H-10 (δ 5.05) with C-9(δ 22.11), C-8(δ39.86) and C-12(δ 25.72) and H-13(δ 1.54) with C-10(δ 124.28) and C-11(δ 131.95) (see Supplementary Figs. S20, S21 and S22 online). The proposed biosynthetic pathway of **3** could be explained by oxidation reaction of methyl group(C-15) to carboxylic acid forming 3-carboxy-*α*-bisabolol. Similar biotransformation reaction was previously reported and proved to be catalyzed by cytochrome P450 enzymes^31^.

### Microbial transformation using *Aspergillus flavus* ATCC 16883

Biotransformation of **1** with *Aspergillus flavus* (ATCC 16883) produced three metabolites (**3**, **4** and **5**). All metabolites were obtained after 7 days’ incubation (10% (**3**), 20% (**4**) and 2% (**5**) yield without optimization). The molecular formula of **4** was established as C_15_H_28_O by HR-ESI-MS analysis which exhibited a peak at m/z 101.11519 corresponding to [M + 2K + H]^+++^ (calcd. = 101.1216) (see Supplementary Fig. S36 online) [*α*]_D_ -40.

The ^1^H-NMR and APT spectra for metabolite **4** were compared with those of *α*-bisabolol (see supplementary Figs. S25, S26, S27 and S28 online), and most of the data were consistent, except for the disappearance of one olefinic proton signal assigned to H-2 (δ_H_ 5.32) with the appearance of two new signals at δ_H_ 1.35 and δ_H_ 1.99, attributed to H-2*α* and H-2*β*, respectively. Additionally, a new signal appeared at δ_H_ 2.17 (1H). APT spectrum showed two new signals at δ_c_ 29.0 (CH_2_) and 43.1 (CH), with the disappearance of C-2 and C-3 signals at δ_c_ 120.5 (CH) and 134.0 (C_q_), respectively. These changes suggest the structure of **4** is 2,3- dihydro- *α* – bisabolol by reduction of the olefinic bond between C-2 and C-3.

The structure was more convinced by the HSQC analysis (see Supplementary Figs. S29, S30 and S31 online) and HMBC ^2^*J* and ^3^*J* correspondences of H-4 with C-2, H-10 with C-12 and C-13, H-9 with C-10 and C-11, H-1 with C-5 and H-14 with C-6 (see supplementary Figs. S32, S33, S34 and S35 online). APT signal of the methyl substituent at C-3 was used to determine its stereochemistry. Resonance value greater than 14.0 ppm indicated an equatorial position, while a signal at less than 12.0 ppm fixed the group as axial^32^. The signal for the methyl group at δ_C_ 14.12 ppm suggests an *α*-configuration for the methyl group at C-3.

The proposed biosynthetic pathway of **4** could be explained by the bio-reduction of carbon–carbon double bond by ene reductase (ER) enzyme at the expense of NAD(P)H transforming *α*-bisabolol to 2,3-dihydro-*α*-bisabolol^33^.

Metabolite **5** was obtained in small amounts. The molecular formula of **5** was established as C_16_H_26_O_3_ (M. wt = 266 Da) by HR-ESI-MS analysis which presented a peak (m/z 248.9641) corresponding to [M- OH] (calcd. = 249.179) (see Supplementary Fig. S43 online). [*α*]_D_ − 70. ^1^H-NMR and APT spectroscopic data of metabolite **5** were analyzed and compared with those of *α*-bisabolol (see supplementary Figs. S39, S40, S41 and S42 online). APT spectrum revealed upfield shifts of two signals assigned to C-10 and C-11 from δ_C_ 124.6 to δ_C_ 66.5 (C-10) and from δ_C_ 131.5 to 62.8 (C-11), this might suggest that the double bond at C-10(11) was replaced by an epoxy group. This change in the APT spectrum was correlated to the disappearance of a triplet assigned to H-10 at δ_H_ 5.08 (1H) with the appearance of a new triplet at δ_H_ 2.71 (1H) in the ^1^H-NMR spectrum. Furthermore, APT spectrum revealed a new signal at δ_C_ 56.2 (CH_3_), a new quaternary carbon signal at δ_C_ 165.3 with disappearance of the signal assigned to C-15 at δ_C_ 23.3, indicating that the methyl group at C-15 was replaced by a new methyl ester group. This was more confirmed by ^1^H-NMR spectrum which showed a new signal at δ_H_ 4.62 (s, 3H), indicative of a methoxy group, and a downfield shift of the signal assigned to H-2 from δ_H_ 5.32 to 6.94 (1H). Finally, ^1^H-NMR showed an upfield shift of a signal assigned to H-14 by 0.24 ppm and a new signal at δ_H_ 1.44 (1H), also APT spectrum showed an upfield shift of a signal assigned to C-14 by 3.68 ppm, disappearance of a quaternary carbon signal assigned to C-7 at 74.2 and appearance of new signal at 42.8 (CH). This might indicate the removal of hydroxyl group at C-7. This was more confirmed by returning the chemical shift value of H-14 to the normal values of methyl hydrogens due to the disappearance of the geminal hydroxyl group at C-7. Suggesting the structure of **5** was 7-dehydroxy-10,11-epoxy-3-methylcarboxy-*α* – bisabolol.

The proposed pathway of **5** (see Supplementary Fig. S52 online) could be explained as follows: first, the methyl group attached to C-3 is oxidized to a carboxylic acid group, forming metabolite **3**, which is then esterified to form 3-methylcarboxy-*α*-bisabolol. This esterification step likely involves an S-adenosyl methionine-dependent methyl-transferase, a member of a large family of enzymes with broad biological activity, including trans-methylation^34^. Second, dehydration occurs leading to the formation of a double bond between C7 and C8, which removes the hydroxyl group from the lateral aliphatic chain of 3-methylcarboxy-*α*-bisabolol. Third, reduction of the newly formed C-7(8) double bond by ene reductase enzyme resulting in formation of 7-dehydroxy-3-methylcarboxy-*α*-bisabolol. Fourth, epoxidation reaction between C-10 and C-11 resulting in the formation of 7-dehyroxy-10,11-epoxy-3-methylcarboxy-*α*-bisabolol. Similar epoxidation reaction was reported before by using *Bipolaris Sorokiniana* to modify *α*-bisabolol^20^. Metabolite **5** is a novel compound which was isolated for the first time from biotransformation of (-)-*α*-bisabolol.

#### In vitro cyclooxygenase-1 (COX-1) and cyclooxygenase-2 (COX-2) enzyme inhibitory assay

The potential of *α*-bisabolol and its metabolites to prevent inflammation was assessed by evaluating their ability to inhibit both COX-1 and COX-2 enzymes, using celecoxib in addition to indomethacin as reference compounds^35,36^ (see supplementary Fig. S53 online). Results (Table 1) indicated that the parent compound (**1**) and its derivatives **3**, **4**, **5** and **6** presented a moderate to potent anti-inflammatory activity against COX-2 enzyme with IC_50_ (minimum inhibitory dose causing 50% activity) ranging from 2.508 to 6.449 μM and COX-2 selectivity index (S.I) ranging from 4.362 to 12.58. Metabolite **5** was slightly more potent as COX-2 inhibitor than *α*-bisabolol with nearly the same selective index, this increased activity of **5** was expected to be related to the presentation of epoxide group in the lateral aliphatic hydrocarbon chain. **6** also showed a moderate anti-inflammatory activity but a significant COX-2 enzyme selectivity which was higher than the parent compound and metabolite** 5**, this may be due to oxidation of CH_3_ at C-15 leading to the presentation of hydroxyl group, increasing its ability to interact with COX-2 enzyme active site. Results were more confirmed using molecular docking studies.

**Table 1:** Results of In vitro COX-1 and COX-2 inhibitory assay of *α*-bisabolol and metabolites 2–6 presented by minimum inhibitory dose causing 50% activity (IC_50_) (μM) and COX-2 selective index (S.I = IC_50_ COX-1/IC_50_ COX-2) reference compounds (indomethacin and celecoxib).

Compound	COX-1 (IC50)	COX-2 (IC50)	S.I
**2**	70.98 ± 4.4	17.34 ± 0.66	4.093
**3**	28.13 ± 1.74	6.449 ± 0.24	4.362
**4**	35.31 ± 2.18	4.107 ± 0.15	8.598
**5**	16.83 ± 1.04	2.508 ± 0.09	6.711
**6**	67.82 ± 2.28	5.39 ± 0.18	12.58
α-Bisabolol	20.139 ± 1.19	2.925 ± 0.13	6.889
Indomethacin*	0.145 ± 0.01	0.456 ± 0.02	0.318
Celecoxib*	21.27 ± 1.32	1.138 ± 0.044	18.69

*Positive controls.

Values were represented as means ± standard deviations (SD) with n = 3.

#### In silico COX-2 enzyme study

**Fig. 2:**
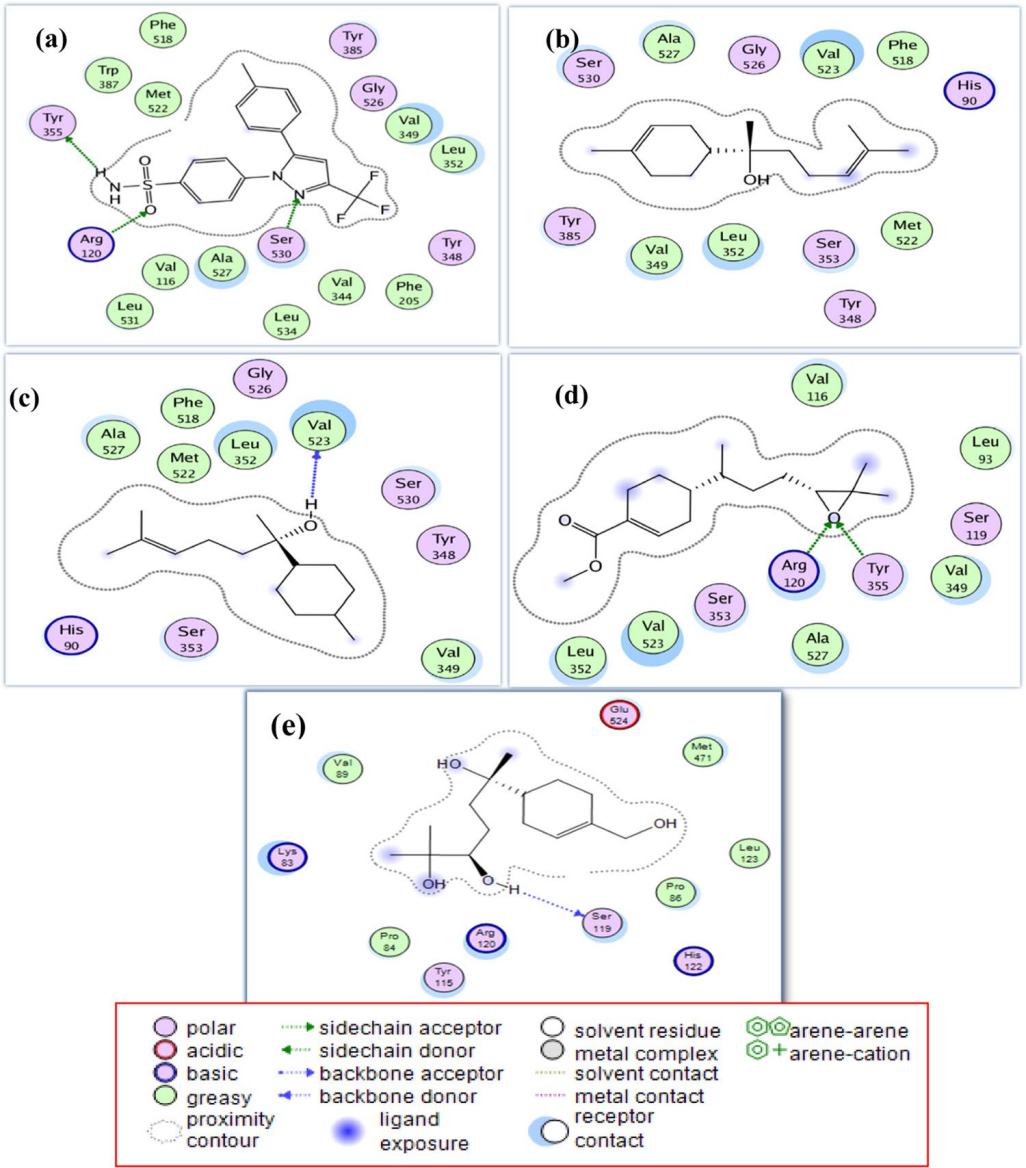
Two-dimensional (2D) binding mode and residues involved in the recognition of (**a**) celecoxib, (**b**) bisabolol, the most potent compounds; (**c**) metabolite 4, (**d**) metabolite 5 and (**e**) metabolite 6 docked and minimized in the COX-2 binding pocket.

*α*-Bisabolol and metabolites (**2**–**6**) were docked into the active site of the COX-2 enzyme, with celecoxib used as a reference inhibitor. Promising binding conformations were selected based on docking scores and binding interactions (see Supplementary Table S4 online). The results demonstrated that metabolites **4**, **5**, and **6** exhibited stronger interactions with the active site of COX-2 enzyme compared to the parent compound, *α*-bisabolol (Fig. 2). Metabolite **5** mimicked celecoxib by interacting with arginine 120 (Arg120) and tyrosine 355 (Tyr355) amino acid residues by hydrogen bonding via the newly introduced epoxide group, besides strong hydrophobic interactions with valine 349 (Val349), leucine 352 (Leu352), and alanine 527 (Ala527). Metabolite **4** also shared celecoxib’s binding profile through strong hydrophobic interactions with Val349, Leu352, and Ala527, and formed a hydrogen bond via its hydroxyl group with Val523. Metabolite **6** exhibited a unique hydrogen bond with serine 119 (Ser119) residue through its lateral hydroxyl group, along with a strong hydrophobic interaction with Tyr115. The COX-2 selectivity of **4**, **5** and **6** could be explained based on the structural differences between COX-1 and COX-2 iso-enzymes. Some of these structural differences are reflected in the substitution of a relatively bulky isoleucine (Ile) residue at position 523 in COX-1 with a valine residue at the same position in the active site of the COX-2 enzyme. This substitution creates a gap in the wall of its channel, allowing access to an additional side pocket^37^. Additional significant structural changes are observed at positions 115 and 119. At position 115, a nonpolar leucine residue in COX-1 is replaced by an uncharged polar tyrosine in COX-2. Similarly, at position 119, a nonpolar valine is substituted with an uncharged polar serine, contributing to the altered binding environment of the COX-2 active site^38^. As shown in Fig. 2, metabolites **4** and **5** interact with Val523, a characteristic amino acid residue located within the COX-2 channel, thereby enhancing their selectivity toward COX-2. In contrast, metabolite** 6** interacts with Tyr115 and Ser119, which are also distinctive residues of the COX-2 enzyme, further supporting its selective binding. The selectivity of **4** and **5** was further explored by docking them into the COX-1 binding site (see supplementary Table S3 online).

#### In vitro evaluation of the neuroprotective activity

Alzheimer’s disease (AD) is one of the most common neurodegenerative disorders. Pathogenesis of AD includes pathological buildup of extracellular plaques (senile plaques) of a 40–42 amino acid peptide called amyloid *β*-protein (A*β*), intracellular aggregation of abnormally phosphorylated tau-protein^39^ and loss of cholinergic neurons in the basal forebrain^40^. The major observed amyloid *β* (A*β*) peptides being A*β*_1–40_ or A*β*_1–42_; and the other peptide fragment A*β*_25–35_ is also a biologically active toxic peptide against the neurons^41^. A*β* in turn causes increased levels of H_2_O_2_ and lipid peroxides to accumulate in cells, increasing the oxidative stress and leading to neurotoxicity^42^. Acetylcholine esterase (AChE) is also playing a vital role in the pathogenesis of Alzheimer by promoting the deposition of insoluble fibrils containing both A*β* and AChE, which are more toxic to cells than A*β* alone^43^. Another hypothesis in the pathogenesis of AD is “cholinergic deficiency hypothesis” which suggests that the symptoms of AD are caused by presynaptic deficiency of acetylcholine. It was also observed that microglial cells were present in larger number and size in brains with AD than in normal brains^44^. So, drugs that have the ability to protect neural cells against amyloid *β*-protein toxicity, inhibit AChE enzyme and enhance the presynaptic acetylcholine release, are predicted to be good potentials in managing the symptoms of AD.

#### Determination of non-cytotoxic concentrations of the tested samples

*α*-Bisabolol and its metabolites **2–5** showed non-cytotoxic influence on SH-SY5Y cells at concentrations: 6.25,12.5 and 25 μM, while metabolite **6** at 25 μM caused significant decrease of cell viability (see Supplementary Fig. S54 online). Positive controls: catechin and epicatechin-3-gallate (ECG) showed the cytotoxic effect only at higher concentration of 200 μM. So, evaluation of the neuroprotective effect of *α*-bisabolol and its bio-transformed metabolites (**2–5**) against the neurotoxicity either induced by H_2_O_2_ or A*β*_1-42_ in SH-SY5Y cells, was tested at non-cytotoxic concentrations: 6.25, 12.5 and 25 μM while metabolite **6** was evaluated for its neuroprotection at only the concetrations of 6.25 and 12.5 μM.

#### Protection against H_2_O_2_ induced-neurotoxicity in SH-SY5Y cells

**Fig. 3:**
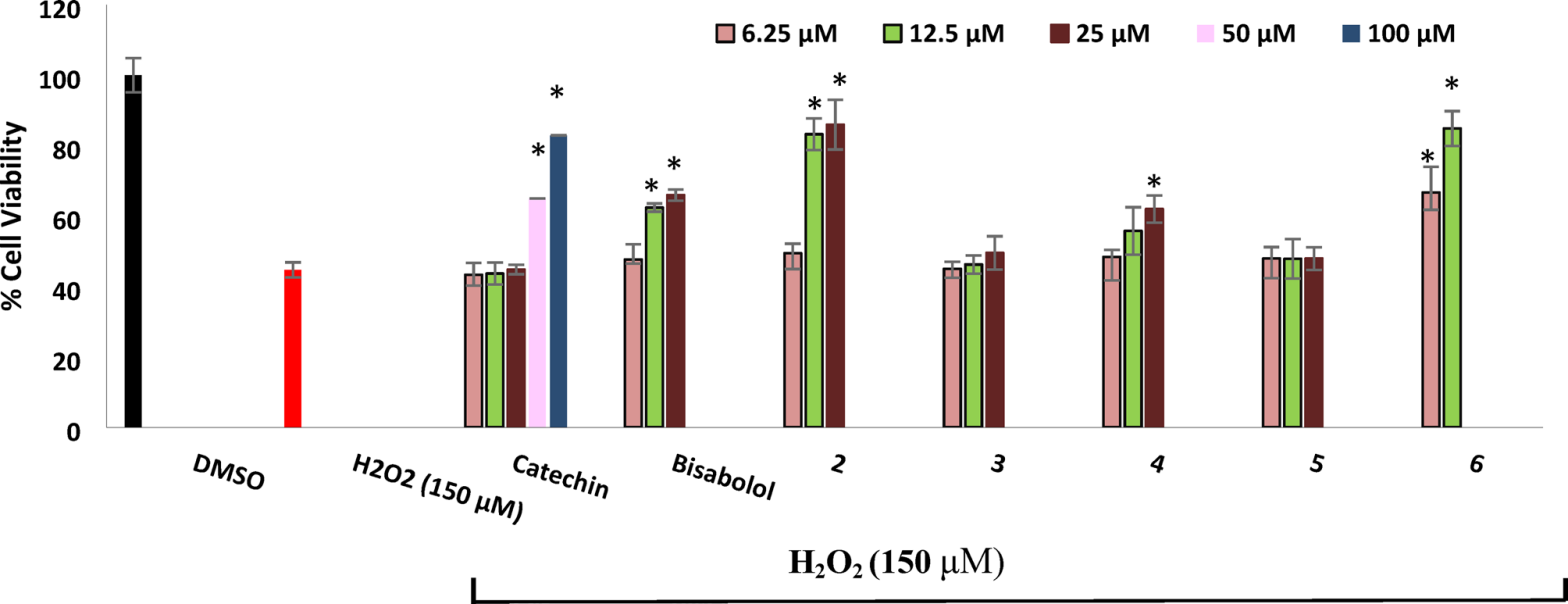
Neuroprotection of bisabolol and its metabolites against H_2_O_2_-induced neurotoxicity in SH-SY5Y cells. Results were represented as means ± standard deviations (SD), with n = 5. * Indicates a statistically significant difference compared to the cell viability of H_2_O_2_-treated cells at *p* < 0.001. Statistical significance was determined using one-way ANOVA followed by Dunnett’s post-hoc test in GraphPad Prism® 10. Metabolites **2** and **6** showed more potent neuroprotection than the parent compound and other metabolites.

**Fig. 4:**
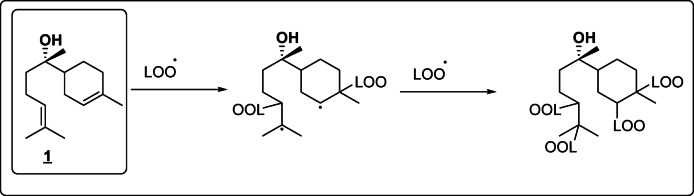
Expected radical scavenging activity of *α*-bisabolol (**1**) by binding to fatty acid peroxyl radicals (LOO·) and stopping the lipid peroxidation chain reaction in the cell membrane.

Results (Fig. 3) outlined that treatment of SH-SY5Y cells with 150 μM of H_2_O_2_ resulted in a reduction of cell viability with about 55.27% when compared with dimethyl sulfoxide (DMSO)-treated cells (as a negative control). Metabolites **6**, **2**, parent compound **1** and metabolite **4** were only the neuroprotective against the toxic effect of H_2_O_2_ in a decreasing order. Bisabolol at 12.5 and 25 μM could significantly increase cell viability of H_2_O_2_ -treated cells with 17.67 and 21.22%, respectively. With higher neuroprotective potential, metabolite **2** doubled the neuroprotective effect of its parent compound by increasing the cell viability of SH-SY5Y cells after H_2_O_2_ exposure with 38.5 and 41.2% at the same concentrations. The most potent neuroprotective activity was achieved by metabolite **6** which at 6.25 and 12.5 μM increased cell viability by 21.95 and 40.13%. Metabolite **4** showed lower protection than bisabolol by increasing cell viability only with 11 and 17.26% while metabolites **3** and **5** didn’t show any neuroprotective activity. It is worth mentioning that only high concentrations of catechin (50 and 100 μM), used as a positive control, exhibited neuroprotective effects with lower potency compared to metabolite **2** at 12.5 and 25 μM. The antioxidant activity is proposed as the underlying mechanism for the neuroprotective effects of *α*-bisabolol and its active derivatives against H_2_O_2_ induced-neurotoxicity. H_2_O_2_ exerts its cytotoxicity by interacting with metal ions in the medium, leading to the generation of highly reactive and toxic hydroxyl radicals, which initiate a lipid peroxidation chain reaction leading to cell membrane damage and neurotoxicity^45^. Therefore, compounds capable of scavenging free radicals and enhancing cell viability are considered biologically active. Although previous studies have demonstrated the antioxidant activity of *α*-bisabolol, its exact mechanism of action remains unclear^46,47^. We hypothesize that *α*-bisabolol may act similarly to vitamin A by binding to fatty acid peroxyl radicals (LOO·), thereby stopping the lipid peroxidation chain reaction in the cell membrane^48^ (Fig. 4). This hypothesis may also explain the reduced antioxidant activity observed in metabolite **4**, in which one of the double bonds, presumed to be involved in radical scavenging, was reduced through the addition of two protons, partially impairing its activity. On the other hand, the higher neuroprotective potential of **2** and **6** could be attributed to their enhanced electron or hydrogen donating capabilities, as they are the di-hydroxyl and tri-hydroxyl derivatives of *α*-bisabolol, respectively. This explanation is further supported by a previous study showing that *α*-Bisabolol *β*-D-fucopyranoside (ABFP), a synthetic glycoside derivative of *α*-bisabolol, exhibited superior scavenging activity against free radicals, H_2_O_2_, and hydroxyl radicals likely owing to the electron or hydrogen donating properties of its sugar moiety^49^.

#### Protection against A_1-42_ induced-neurotoxicity in SH-SY5Y cells

**Fig. 5:**
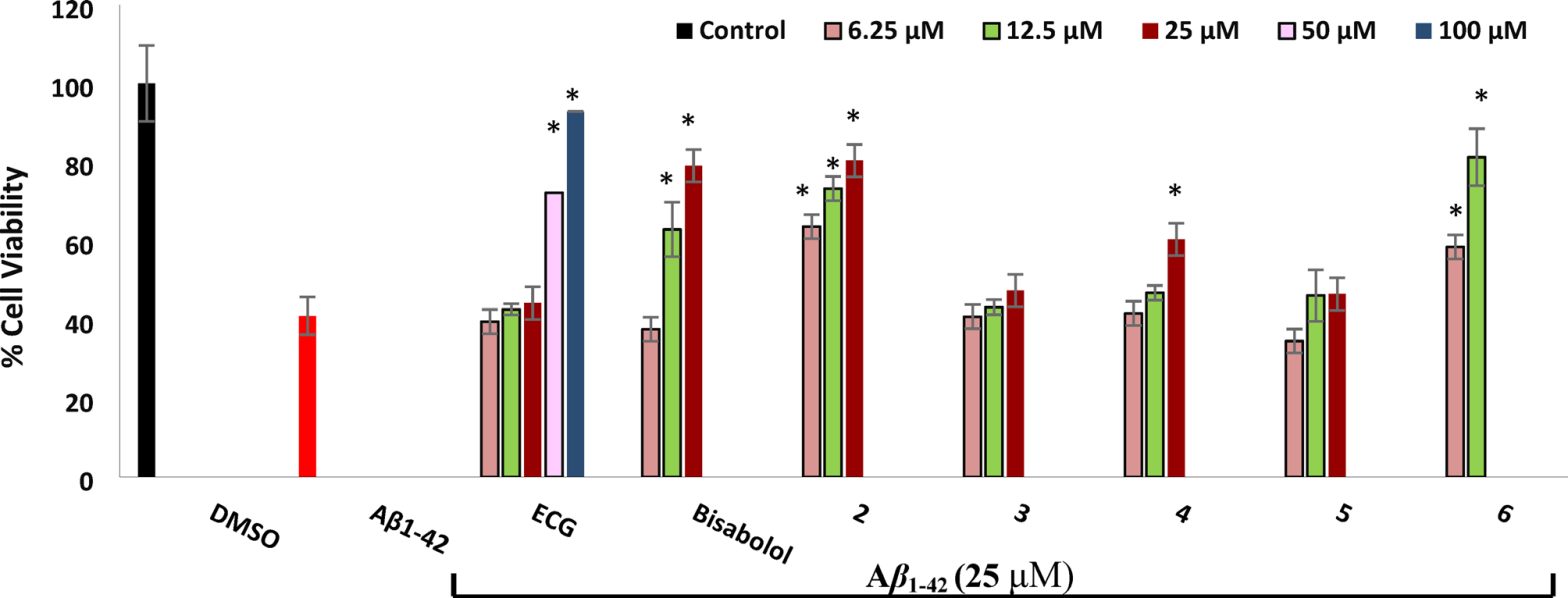
Neuroprotection against A*β*_1-42_ induced-neurotoxicity in SH-SY5Y cells. ECG: Epigallocatechin-3-gallate was used as a positive control. Values were represented as means ± standard deviations (SD), n = 5. * Indicates a statistically significant difference compared to the cell viability of A*β*_1-42_ -treated cells at *p* < 0.001*.* Statistical significance was determined using one-way ANOVA followed by Dunnett’s post-hoc test in GraphPad Prism® 10. Metabolites **2** and **6** were more neuroprotective than *α*-bisabolol and other metabolites.

Cultivation of SH-SY5Y cells with aggregated A*β*_1-42_ (25 μM) for 24 h resulted in 59% reduction of cell viability (Fig. 5). Clearly, prior treatment of the cells with *α*-bisabolol **(1)** and metabolites **2** and **6** for 24 h produced significant protection. **2** and **6** showed more protection than *α*-bisabolol as it increased cell viability by 22.6, 32.3 and 39.44% (**2** at 6.25, 12.5 and 25 μM) and 17.47 and 40.3% (**6** at 6.25 and 12.5 μM) while bisabolol increased it by 21.92 and 38.14 at 12.5 and 25 μM (bisabolol at 6.25 μM showed no protection). Metabolite **4** showed lower protection at only 25 μM, increasing cell viability by 19.4%. Interestingly, epicatechin-3-gallate, used as a positive control, exhibited neuroprotection only at 50 and 100 μM. It was previously reported that *α*-bisabolol had protective abilities against (A*β*_23-35_) induced neurotoxicity by preventing the formation of oligomers, disaggregation of already formed mature fibrils^8^ and it significantly scavenged the reactive oxygen species (ROS) and reactive nitrogen species (RNS) produced from A*β* toxicity^13^. In our study metabolites **2** and **6** were more potent as neuronal protective against A*β*_1-42_ than *α*-bisabolol. This could be explained by their expected higher ROS and lipid peroxyl radicals scavenging activity than *α*-bisabolol due to their increasing electron or hydrogen donating activity^49^, leading to reducing oxidative stress inside cells and thus protecting against A*β* toxicity.

#### Acetyl cholinesterase (AChE) inhibitory activity

**Fig. 6:**
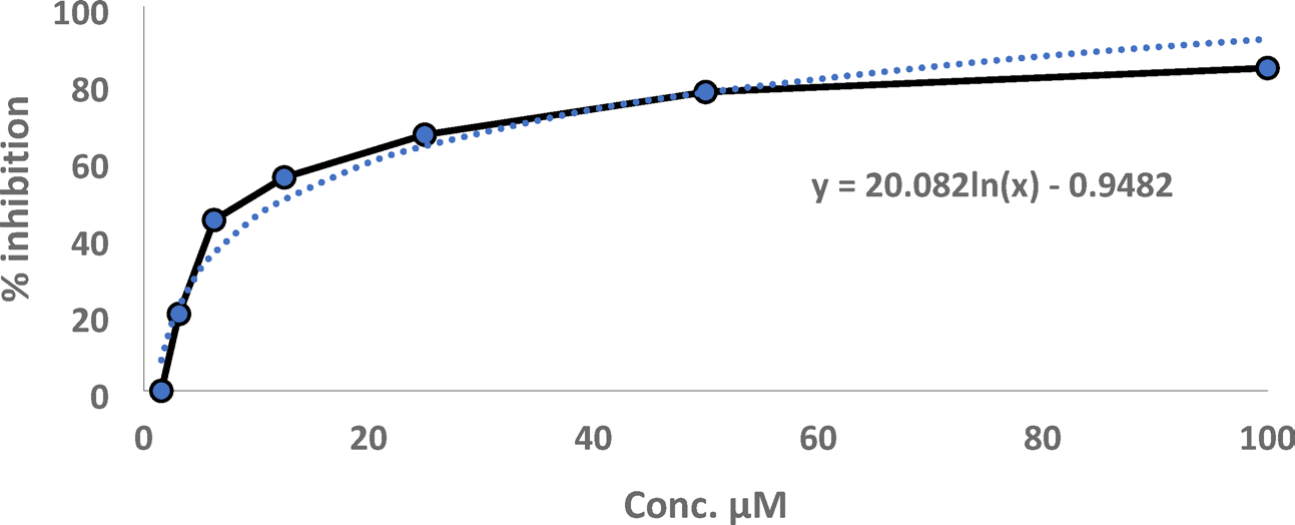
Dose–response curve of AChE inhibition by metabolite **2**.

Among the tested samples, the results (see Supplementary Table S5 online) revealed that only **2** had the inhibitory activity against AChE with IC_50_ value of 12.94 µM (Fig. 6). Metabolite **3** showed very weak activity (20% inhibition) at 100 µM. The parent compound **1**, along with metabolites **4**, **5**, and **6**, showed no inhibitory activity against AChE at concentrations up to 100 µM. The inhibitory activity of **2** was further investigated by molecular docking studies.

#### Molecular docking simulation against acetylcholine esterase active site

**Fig. 7:**
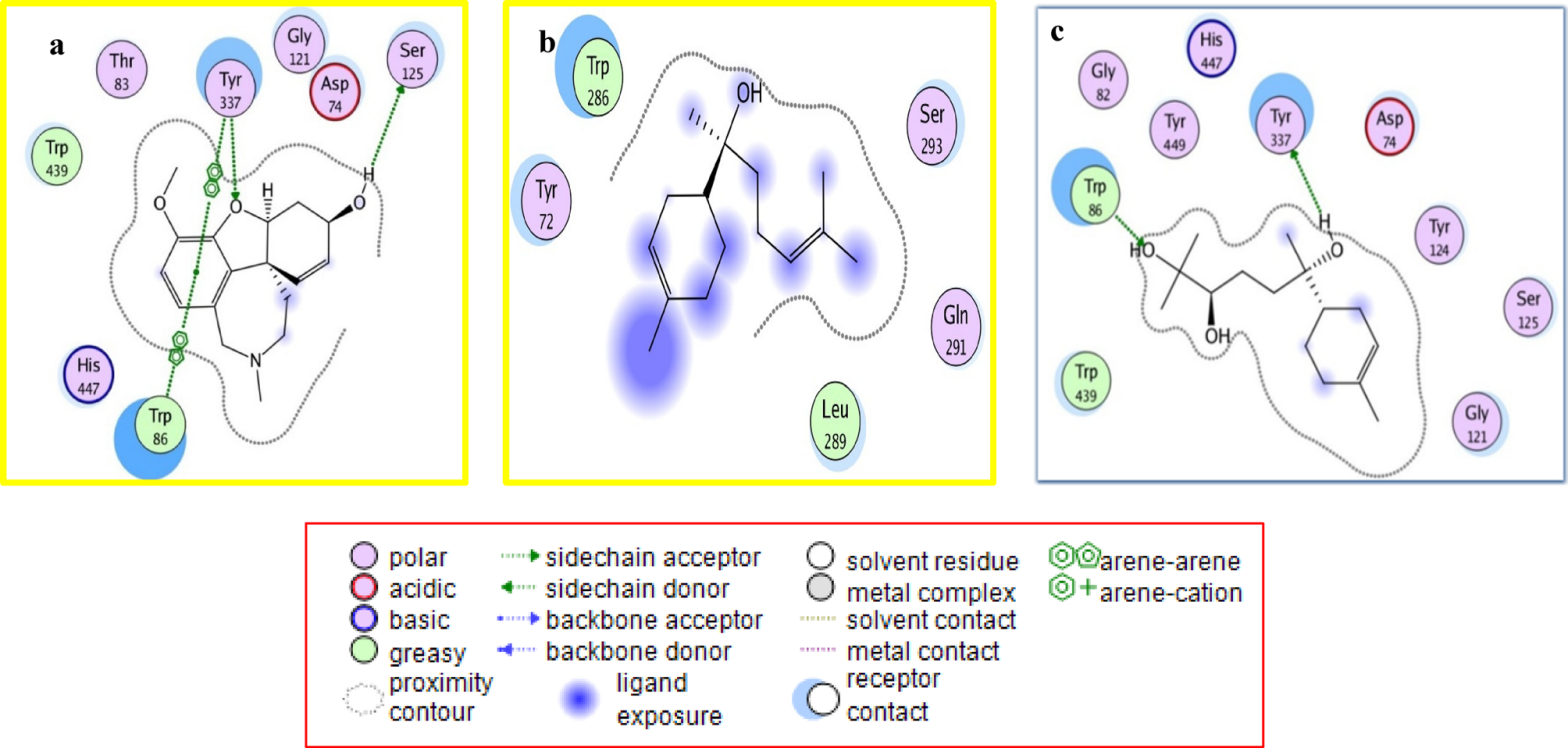
2D binding mode and residues involved in the recognition of (**a**) galanthamine, (**b**) *α*-bisabolol and (**c**) the most potent compound (metabolite 2) docked and minimized in the AChE binding pocket.

Docking results (see Supplementary Table S6 online) of the newly biosynthesized metabolite **2** to the active site of acetylcholinesterase, with galanthamine as standard inhibitor, presented the varieties of binding interactions and binding scores of **2** compared to that of the starting compound *α*-bisabolol (Fig. 7). Metabolite **2** showed better binding affinity than starting compound *α*-bisabolol. **2** shared galanthamine in binding with Tryptophan 86 (Trp86) and Tyr337 amino acid residues via hydrogen bonds through its alcoholic hydroxyl moieties. It also bound with Trp86 and Tyr337 amino acid residues via strong hydrophobic interactions.

#### Drug-likeness and ADMET (absorption, distribution, metabolism, excretion and toxicity) prediction

*α*-Bisabolol and metabolites **2–6** were tested whether they obey Lipinski’s rule of five or not utilizing Swiss ADME predictor^50^. Lipinski’s Rule of Five (RO5) is widely used to estimate drug-likeness and oral bioavailability of compounds based on their physicochemical properties. According to RO5, an ideal oral drug should have a molecular weight < 500 g/mol, calculated partition coefficient (clog P) < 5, no more than 5 hydrogen bond donors (HBD), and no more than 10 hydrogen bond acceptors (HBA)^51^. Additional criteria include a polar surface area (PSA) ≤ 140 Å^2^ and fewer than 10 rotatable bonds, are required to improve the predictions of oral bioavailability^52^. Results (see Supplementary Table S7 online) showed that *α*-bisabolol and its metabolites (**2–6)** obey Lipinski’s rule of five, indicating good oral absorption of the compounds through gastrointestinal tract (GIT). Further evaluation was performed by calculating the pharmacokinetic ADMET properties using preADMET prediction tools^53^. Results (see Supplementary Table S8 online) revealed that they could be easily absorbed from GIT with high percentage of human intestinal absorption (HIA%) ranging from 80 to 100% except for metabolite 6 that have moderate HIA% (72.70). Their calculated aqueous solubility (log S) values ranged from -2.82 to -4.37 that proved acceptable solubilization in intestinal fluids prior to absorption. *α*-Bisabolol and metabolites **2–5** were predicted to be non-inhibitors of cytochrome CYP3A4 enzyme, an important enzyme for the metabolism of drugs in humans, that indicates low probability of drug-drug interaction. In addition, carcinogenicity tests indicated that all tested compounds were non-carcinogenic. Moreover, the human ether-a-go-go related gene (hERG) K^+^ channel inhibition prediction showed that *α*-bisabolol and metabolites **2–4**, **6** had a low risk of blocking (hERG) K^+^ channel and so, causing no fatal cardiac arrhythmias.

Although lipophilicity plays a crucial role in BBB permeability, it can’t be considered as the sole determinant. Excessive lipophilicity can lead to greater distribution volumes, increasing metabolic liability and lowering plasma and brain levels of unbound drug. Simple physicochemical measurements and calculations can provide a preliminary prediction of a compound’s ability to cross the blood–brain barrier (BBB) and reach the central nervous system (CNS). Key properties associated with BBB permeability include limited molecular weight (typically < 450 Da), clog P (2–4), HBD (> 3) and low polar surface area (< 90 Å^2^)^54,55^. Although, α-bisabolol meets these criteria, its lipophilic nature (clog P = 4.23) may reduce its bioavailability and consequently limit its ability to cross the blood–brain barrier (BBB). When comparing the most neuroprotective metabolites (**2** and **6**), metabolite 2 presented the most of the criteria for BBB permeability (clog P = 2.27; MW = 256.38 g/mol; TPSA = 60.69 Å^2^), except for the number of hydrogen bond donors (HBD = 3), slightly exceeding the suggested limit (< 3). In contrast, metabolite **6** didn’t show a fulfillment of BBB permeability requirements (HBD > 3; clog P < 2).

Regarding cyclooxygenase-2 inhibitory activity, metabolite **5** showed higher potency than α-bisabolol and exhibited a better potential to cross the BBB due to favorable physicochemical properties (clog P = 3.51; MW = 266.38 g/mol; TPSA = 38.83 Å^2^; HBD = 0). However, it possessed a moderate risk of blocking the hERG potassium channel, which may lead to potentially fatal cardiac arrhythmias.

This study suggested that biotransformation of bisabolol into the di-hydroxyl derivative enhanced its polarity, neuroprotective and acetylcholine esterase inhibitory activity with limited enhancing of BBB permeability. It would be of interest to validate this hypothesis using an in vitro blood–brain barrier (BBB) model that closely mimics the in vivo conditions. Furthermore, additional investigations are required to evaluate the impact of efflux systems and molecular transporters at the BBB on the uptake of these compounds.

### Conclusion

Five metabolites were isolated through fungal biotransformation of (-)-*α*-bisabolol. Metabolites **2** (10*β*,11-dihydroxy-*α*-bisabolol) and **3** (Hamanasic acid A) are known natural products but reported here for the first time as biotransformation metabolites of *α*-bisabolol. Metabolites **4** (2,3-dihydro-*α*-bisabolol),**5** (7-dehydroxy-10,11-epoxy-3-methylcarboxy-*α*-bisabolol**)** and **6** (10*β*,11,15-trihydroxy-*α*-bisabolol) were a newly identified natural derivatives of *α*-bisabolol. In vitro COX-1 and COX-2 enzyme inhibitory assays showed that metabolite **5** has marked inhibition of both COX-1 and COX-2 enzymes, with slightly greater potency against COX-2 compared to *α*-bisabolol. However, its ADMET predictions indicated a moderate risk of blocking (hERG) K^+^ channel, which may be associated with potentially fatal cardiac arrhythmias.

In vitro evaluation of the neuroprotective activity revealed that both metabolites **2** and **6** exhibited strong neuroprotective effects against H_2_O_2_- and A*β*_1-42_-induced toxicity. Notably, metabolite **2** also demonstrated significant AChE inhibitory activity, supporting its potential as a lead compound for production of new neuroprotective agents. Concisely, the in vitro and in silico results suggested that metabolite **2** is a promising candidate for further investigations aiming to explore new therapeutic effects targeting acetylcholine deficiency, neurotoxicity, and neuro-inflammation which are the common pathogenic profile of various neurodegenerative diseases.

### Study limitations

This study included in silico and in vitro results which are predictive and may not reflect the actual biological efficacy so, further in vivo validation is mandatory to investigate both the therapeutic potential and the safety at the physiological systems. In addition, development of the metabolites through biotransformation technique may exhibit product variability with the changes in the culture or strain conditions.

## Materials and methods

### Chemicals

(-)-*α*-bisabolol (≥ 93% v/v) was obtained from (SIGMA-ALDRICH, Milwaukee, WI, USA).

### Microorganisms

The microbial strains were obtained from the American Type Culture Collection (ATCC), Tokyo, Japan; the Northern Regional Research Laboratory (NRRL); and the Assiut University Mycological Centre (AUMC), Egypt. The cultures were preserved on potato dextrose agar (PDA) slants at (4 °C). The following 22 microorganisms were used in the biotransformation screening; *Penicillium brevicompactum* ATCC 9056 (F. *Aspergillaceae*)*, Penicillium glabrum* ATCC 20468 (F. *Aspergillaceae*)*, Aspergillus niger* ATCC 10549, NRRL 328 and NRRL 599 (F. *Aspergillaceae*)*, Aspergillus versicolor* AUMC 150201 (F. *Aspergillaceae*)*, Rhizopus species* ATCC 36060 (F. *Mucoraceae*)*, Cunninghamella elegans* NRRL 1392 (F. *Cunninghamellaceae*)*, gyminascella citrina* NRRL 6050 (F. *Gymnascaceae*)*, Cordyceps sinensis* ATCC 24400 (F. *Ophiocordycipitaceae*), *Aspergillus flavus* ATCC 16883 (F. *Aspergillaceae*), *Alternaria alternata* ATCC 10801 (F. *Pleosporaceae*), *Oudemasiella mucida* ATCC 58725 (F. *Physalacriaceae*), *Cunninghamella black* NRRL 1369 (F. *Cunninghamellaceae*), *Cunninghamella elegans* ATCC 8688a (F. *Cunninghamellaceae*), *Phlebia rufa* ATCC 76992 (F. *Meruliaceae*), *Penicillium chrysogenum* ATCC 9480 (F. *Aspergillaceae*), *Alternaria chartarum* ATCC 26683 (F. *Pleosporaceae)*, *Cordyseps ophioglossoids* ATCC 24400 (F. *Ophiocordycipitaceae*), *Coriolus versicolor* ATCC 20565 (F. *Polyporaceae*), *Phanerochaete chrysosporium* ATCC 34541(F. *Phanerochaetaceae*) and *Mucor* sp. ATCC 56651 (F. *Mucoraceae*).

*Cordyceps sinensis* ATCC 24400, *Aspergillus flavus* ATCC 16883 and *Alternaria alternata* ATCC 10801 were chosen for large scale fermentation as they are the most efficient microorganisms capable of transforming bisabolol to its metabolites and as they reproducibly produced metabolites.

### Screening procedures

The screening process was conducted following a two-stage fermentation protocol, using α –medium as previously reported in the literatures^56,57^.

*Stage I*: Surface growth from fresh fungal slants was suspended in 5 mL of sterile liquid medium. This suspension was transferred into 250 mL Erlenmeyer flasks containing 50 mL of sterile medium. Cultures were incubated at 27 °C for 72 h with mild agitation at 200 rpm in a temperature-controlled rotary shaker incubator.

*Stage II*: A 5 mL aliquot from the Stage I culture was inoculated into fresh 250 mL Erlenmeyer flasks containing 50 mL of sterile medium. After incubation for 24 h under the same conditions, substrate suspension was prepared by dissolving 500 mg of (-)-*α*-bisabolol in 1 mL of the suitable solvent (dimethyl sulfoxide (DMSO)), then 10 μL of the prepared suspension were added to each flask. Samples were collected every 24 h over a period of two weeks. Substrate and culture controls were included in parallel.

### Scale-up biotransformation

Scale-up biotransformation was performed using the most efficient fungal strains capable of converting (-)-*α*-bisabolol into its metabolites with the highest yield. The procedure followed the same steps as the initial screening, with modifications in culture volume. Specifically, the entire 25 mL culture from Stage I was transferred into 500 mL Erlenmeyer flasks containing 100 mL of fresh sterile medium. After 24 h of incubation at 27 °C with agitation (200 rpm), 100 μL of the substrate suspension (500 mg (-)-*α*-bisabolol/ 1 mL DMSO) was added to each flask. Following the biotransformation period, the resulting metabolites were extracted, isolated, and purified using column chromatography techniques.

### Isolation and purification of the metabolites

The fermentation broth of *Cordyceps sinensis* (ATCC 24400) incubated with (-)-α-bisabolol for 14 days was filtered and exhaustively extracted with ethyl acetate (EtOAc) (3 × 1 L). The combined EtOAc extracts were dried over anhydrous sodium sulfate and concentrated under reduced pressure, yielding 250 mg of a yellow residue. This crude extract was subjected to silica gel column chromatography (16 × 1.5 cm, 24 g), using a gradient elution starting with 100% dichloromethane (DCM), followed by increasing proportions of EtOAc in DCM. Fractions eluted with 40% EtOAc in DCM afforded pure metabolite **2** (40 mg), while those eluted with 45% EtOAc in DCM yielded pure metabolite **6** (5 mg). TLC analysis (silica gel GF_254_) showed single violet spots after spraying with *vanillin–sulfuric* acid reagent and heating: R*f* = 0.25 for metabolite **2** and R*f* = 0.22 for metabolite **6** in the solvent system (DCM: EtOAc, 1:4, v/v).

Termination of enzymatic reaction of *Alternaria alternate* (ATCC 10801) was done after 7 days’ incubation with bisabolol by complete extraction (1L × 3) with EtOAc. The combined extracts were concentrated by drying and then evaporated under reduced pressure to yield 200 mg yellow residue. Chromatographic isolation on silica gel (20 × 1.5 cm, 30 g) using a DCM–EtOAc gradient elution yielded pure metabolite **3** (30 mg) from the 20% EtOAc in DCM fraction. Silica gel GF_254_ TLC plate of collected fractions revealed a single major violet spot at *R*_*f*_ = 0.32 in (Petroleum ether: EtOAc, 7:3, v/v) after spraying with *vanillin-sulfuric* acid reagent and heating.

The fermentation broth of *Aspergillus flavus* (ATCC 16883) after 7 days’ incubation with bisabolol was was treated similarly. Extraction with EtOAc (3 × 1 L) and evaporation yielded 200 mg of residue. Column chromatography on silica gel (16 × 1.5 cm, 24 g) with a DCM–EtOAc gradient elution afforded pure metabolite **5** (4 mg) from the 4% EtOAc in DCM fraction and metabolite **4** (40 mg) from the 25% EtOAc in DCM fraction. TLC analysis revealed single violet spots after *vanillin–sulfuric* acid spray and heating: metabolite **5** at R*f* = 0.58 in DCM: EtOAc (2:1, v/v) and metabolite **4** at R*f* = 0.22 in petroleum ether: EtOAc (7:3, v/v).

### Spectroscopic analysis

#### NMR spectroscopy

^1^H-NMR and APT spectra were obtained using a BRUKER Ascend™ 400 spectrometer or BRUKER DRX 600 NMR spectrometer operating at 400 and 100 MH, respectively. Tetramethylsilane (TMS) was used as internal chemical shift standard and CDCl_3_ as the solvent. The chemical shift (δ) values were expressed in parts per million (ppm), with TMS resonance used as the reference. All NMR data is available in the supplementary data online.

#### Mass analysis

The formulas of the metabolites were confirmed by HR-ESI-MS using LC-MS-Q-TOF at Natural Products Research Lab, Fayoum University and also by EI-MS using EI mode on Direct Inlet part to mass analyzer in Thermo Scientific GCMS model ISQ at the Regional Center for Mycology and Biotechnology (RCMB), Al-Azhar University, Nasr City, Cairo. All MS analysis data is available in the supplementary data online.

#### FTIR analysis

The obtained IR spectra were scanned using JASCO FTIR-6200 instrument at the Regional Center for Mycology and Biotechnology (RCMB), Al-Azhar University, Nasr City, Cairo. All IR analysis data is available in the supplementary data online.

#### Optical rotation measurement

Optical rotation was acquired usinga Jasco P-1020 digital polarimeter at faculty of science, Kfr Elsheikh University. UV–vis 2550.

### Biological assay

#### Anti-inflammatory assay

The ability to inhibit COX-1 and COX-2 enzymes was evaluated as previously reported^35,36^, using COX-1 Cayman human enzyme inhibitory assay kit (No.548, USA), COX-2 Cayman human enzyme inhibitory assay kit (No.547, USA) and Tecan Spark reader. Indomethacin (Sigma-Aldrich, USA) was used as reference COX-1 inhibitor, Celecoxib® (Sigma-Aldrich, USA) was used as reference COX-2 inhibitor. The % Relative Inhibition was calculated as follows:

$$\% relative\;inhibition = \frac{{slope\;of\;EC{ - }slope\;of\;S}}{slope\;of\;EC} \times 100$$

The minimum inhibitory dose causing 50% activity (IC_50_) and the selectivity indices (SI) of the tested/reference compounds were calculated

$$SI = \frac{{IC_{{50}} \;of\;COX1}}{{IC_{{50}} \;of\;COX2}}$$

### In vitro neuroprotective assay

#### Determination of the non-cytotoxic concentrations of the tested samples

In this assay, the non-toxic concentrations of the tested samples to SH-SY5Y cells, along with catechin^58^ and epicatechin-3-gallate^59^ as a positive controls were used for the evaluation of the neuroprotective activity. To determine these concentrations, SH-SY5Y cells obtained from the American Type Culture Collection (ATCC) (Manassas, VA, USA) were cultured and treated with various concentrations of the tested samples (6.25, 12.5, 25, 50, 100, and 200 μM). Cell viability was then assessed using a previously described method^56^.

#### Protection against H_2_O_2_ induced-neurotoxicity in SH-SY5Y (human neuro-blastoma) cells

H_2_O_2_ concentration that caused 50% reduction in cell viability was evaluated as previously reported^60^. Then the cultured cells (2 × 10^4^ cells/well) were incubated with test samples (at non-cytotoxic concentrations: 6.25, 12.5, 25 μM) or catechin as a positive control (at non-cytotoxic concentrations: 6.25, 12.5, 25, 50, 100 μM). Lastly, the WST-1 reagent was used to evaluate the cell viability as reported^56^*.*

#### In vitro evaluation of the protection against Aβ_1-42_ induced-neurotoxicity in SH-SY5Y cells

From previous results^56^, it was found that 48 h incubation of SH-SY5Y cells (1 × 10^4^ cells/ well) with A*β*_1_-_42_ at a concentration of 25 μM caused a 50% fall of cell viability. So, the capability of (-)-*α*-bisabolol and its metabolites to protect the cells against the cytotoxic effects of A*β*_1_-_42_ was evaluated by treating SH-SY5Y cells with the test samples at the non-cytotoxic concentrations or epicatechin-3-gallate as a positive control at the non-cytotoxic concentrations and after one day the media was changed by a new fresh one, then the induction of neurotoxicity was exerted by incubation of A*β*_1_-_42_ (25 μM) with the pretreated cells. Finally, evaluation of cell viability was conducted.

#### Acetylcholinesterase inhibitory assay

The inhibitory activity of (-)-*α*-bisabolol and its metabolites on the AChE enzyme was assessed using Ellman’s method and The % of enzyme inhibition was calculated as previously fully described^60^.

#### Molecular docking study

The docking study was conducted following the procedure outlined in the literature references^61,62^. Crystal structures were retrieved from the RCSB Protein Data Bank (PDB). The structure of cyclooxygenase-2 (COX-2) in complex with meclofenamic acid, serving as the co-crystallized ligand, was used for the study, PDB code: 5IKQ^63^. Human acetylcholinesterase (AChE) in complex with huprine W and fasciculin 2 as the co-crystallized ligands (ID: 4BDT) was used in the study^64^. The targets were prepared, and the active site was identified following the procedures outlined in the literature^61,62^. The docking experiments were carried out using the Triangle Matcher Placement and Rigid Receptor Refinement methods. The free binding energy of the studied ligands was evaluated using the London dG and GBVI/WSA dG scoring functions. A more negative score (lower binding energy) indicates a higher likelihood of the molecule docking with the structure and forming more favorable interactions.

#### Drug-likeness and ADMET prediction

Swiss ADME predictor^50^ was used to evaluate either *α*-bisabolol and metabolites **2–6** obey Lipinski’s rule of five or not. The pharmacokinetics ADMET properties were also calculated via preADMET predictors^53^.

### Statistical analysis

The half-maximal inhibitory concentrations (IC_50_) were calculated from the regression equations of the curves prepared in Microsoft Excel 2010 by plotting the % inhibition against sample concentrations. Cell viability was expressed as a percentage of WST-1 reduction, considering that the viability of cells treated with 1% DMSO as a negative control was 100%. Figures were built in Microsoft Excel 2010. All values were the mean ± SD. The values were the average of three independent experiments. Statistical significance was determined using one-way ANOVA followed by Dunnett’s post-hoc test in GraphPad Prism® 10 (Version 10.0.3, GraphPad Software, Inc., USA).

### Spectroscopic data of metabolites

#### 10*β*,11-dihydroxy-*α*-bisabolol (2)

Colorless oil, [*α*]_D_ − 48.7. **IR **_***v* max**_ cm^-1^ (see Supplementary Fig. S14 online): 3442.5, 2923, 2853, 1637 and 772. **HSQC** spectrum (see Supplementary Figs. S8 and S9 online).

#### (-)-Hamanasic acid A (3)

Colorless oil, [*α*]_D_ – 63.0. **IR**_***v*max**_ cm^-1^ (see Supplementary Fig. S24 online): 3443, 2924, 2853, 1649 and 772. **HSQC** spectrum (see Supplementary Fig. S19 online).

#### 2,3- dihydro- *α* – bisabolol (4)

Colorless oil, [*α*]_D_ -40. **IR**_***v*max**_ cm^-1^ (see Supplementary Fig. S38 online): 3442.6, 2955, 2924, 2854, 1644 and 770. **HR-ESI–MS**: m/z, [M + 2K + H]^+++^ (calcd. For C_15_H_28_O, 101.1216; found 101.11519). **EI-MS** (see Supplementary Fig. S37 online): m/z (relative intensity) 224.72 (33) [M]^+^, 205.82 (8) [M-H_2_O]^+^, 102.92 (100), 103.61 (21), 115.56 (10), 117.40 (12), 131.29 (20), 133.02 (33), 146.25 (5), 146.87 (9), 160.11 (29), 163.26 (29), 175.6 (31). ^**1**^**H-NMR** (CDCl_3_, 400 MHz): δ 0.79 (m, 3H), 1.04 (s, 3H), 1.55 (s, 3H), 1.62 (s, 3H), 1.28 (m, 1H),1.25 (m,1H), 1.35 (m, 1H),1.97 (m, 1H), 1.18 (m, 2H), 1.75(m, 2H), 1.86 (m, 2H), 1.94 (m, 2H), 1.99 (m, 1H), 2.17 (m,1H), 5.05 (t, 1H). **APT** (CDCl_3_, 100 MHz): δ 17.1 (CH_3_), 17.6 (CH_3_), 24 (CH_3_), 25.7 (CH_3_), 22.1 (CH_2_), 25.7 (CH_2_), 26.4 (CH_2_), 29.0 (CH_2_), 29.7 (CH_2_), 39.6 (CH_2_), 43.1 (CH), 47 (CH), 74.4 (Cq) 124.4 (CH), 132(Cq).

#### 7-des-hydroxy -10,11-epoxy- 3-methylcarboxy- *α* – bisabolol (5)

Colorless oil, [*α*]_D_ − 70. **IR**_*v*max_ cm^-1^ (see supplementary Fig. S45 online): 1459.8, 772.48, 1733.45, 1219.18. **HR-ESI–MS**; m/z: [M- OH] (calcd. for C_16_H_26_O_3_, 249.179; found, 248.9641). **EI-MS** (see supplementary Fig. S44 online): m/z (relative intensity): 233.64(21) [M- H_2_O- CH_3_]^+^, 207.61(33) [M- CO_2_Me]^+^, 195.39(25) [M-C_4_H_7_O]^+^ , 154.98 (47) [M- C_7_H_12_O]^+^, 104.26 (31), 105.29 (100), 123.76 (23), 130.45 (28), 134.87 (44), 137.34 (22), 151.41 (38), 154.98 (47), 161.99 (60), 164.16 (37). ^**1**^**H-NMR** (CDCl_3_, 400 MHz): δ 0.81 (d, 3H), 1.15 (m, 2H), 1.18 (s, 6H), 1.27 (m, 2H), 1.44 (m, 1H), 1.46 (m, 1H), 1.79 (m, 2H), 1.85 (m, 2H), 2.05 (m, 2H), 2.71 (t, 1H), 4.62 (s, 3H), 6.94 (dd, 1H). **APT** (CDCl_3_, 100 MHz): δ 19.7 (CH3), 20.6 (CH2), 23.4 (2 CH3), 26.4 (CH2), 30.1 (CH2), 32.2 (CH2), 34.7 (CH2), 39.8 (CH), 42.8 (CH), 56.2 (CH3), 62.8 (Cq), 66.5 (CH), 129.8 (CH), 133.8 (Cq), 165.3 (Cq).

#### 10*β*,11,15-trihydroxy- *α* – bisabolol (6)

Colorless oil, [*α*]_D_ − 53.4, **IR**_*v*max_ cm^-1^ (see Supplementary Fig. S50 online): 3442.5, 2918 1646, 708. HR-ESI–MS spectrum: *m/z:* [M + K + H]^++^, calcd. For C_15_H_28_O_4_, 156.125; found, 156.05156). ^**1**^**H-NMR** (CDCl_3_, 400 MHz): δ 1.15 (s, 3H), 1.18 (s, 6H), 1.33 (m, 2H), 1.55 (m, 1H), 1.61 (m, 2H), 1.91 (m, 2H), 1.95 (m, 2H), 1.97 (m, 2H), 3.57 (br.s, 1H), 3.65 (s, 2H), 5.65 (br.s, 1H). **APT** (CDCl_3_, 100 MHz): δ 23.4 (CH_2_), 24.2 (CH_3_), 24.9 (CH_2_), 25.7 (2 CH_3_), 27.7 (CH_2_), 31.9 (CH_2_), 39.4 (CH_2_), 43 (CH), 63.7 (CH_2_), 70.9 (Cq), 74.3 (CH), 75.0 (Cq), 124.03 (CH), 135.9 (Cq).

## Electronic supplementary material

Below is the link to the electronic supplementary material.

Supplementary Material 1

## Data Availability

All data generated or analyzed during this study are included in this published article (and its supplementary information files).
