# Supplementary material for: In vitro and in silico neuroprotective evaluation of new biotransformation metabolites of (-)-α-bisabolol
Source: Sci Rep. 2025 Jul 26;15:27206. doi: 10.1038/s41598-025-11694-4 (PMC12297533; doi:10.1038/s41598-025-11694-4)
Supplement: Supplementary file 1 — Supplementary Material 1 [file 41598_2025_11694_MOESM1_ESM.pdf]

Supplementary

Table of Contents	Page
Figure S1. ^{1}H NMR spectrum of compound 1 ($CDCl_3$, 400 MHz).	5
Figure S2. ^{1}H NMR spectrum expansion of compound 1 ($CDCl_3$, 400 MHz).	6
Figure S3. APT spectrum of compound 1 ($CDCl_3$, 100 MHz)	7
Figure S4. ^{1}H NMR spectrum of compound 2 ($CDCl_3$, 400 MHz).	8
Figure S5. ^{1}H NMR spectrum expansion (1 – 1.7 ppm) of compound 2 ($CDCl_3$, 400 MHz).	9
Figure S6. ^{1}H NMR spectrum expansion (3 - 5.6 ppm) of compound 2 ($CDCl_3$, 400 MHz).	10
Figure S7. APT spectrum of compound 2 ($CDCl_3$, 100 MHz).	11
Figure S8. HSQC spectrum expansion of compound 2.	12
Figure S9. HSQC spectrum expansion of compound 2.	13
Figure S10. HMBC spectrum of compound 2 ($CDCl_3$, 100 MHz).	14
Figure S11. HMBC spectrum expansion of compound 2 ($CDCl_3$, 100 MHz).	15
Figure S12. NOESY spectrum of compound 2 ($CDCl_3$, 100 MHz).	16
Figure S13. ESI- mass spectrum of compound 2.	17
Figure S14. IR spectrum of compound 2.	18
Figure S15. ^{1}H NMR spectrum of compound 3 ($CDCl_3$, 400 MHz).	19
Figure S16. ^{1}H NMR spectrum expansion (1-2.5 ppm) of compound 3 ($CDCl_3$, 400 MHz).	20
Figure S17. ^{1}H NMR spectrum expansion (5-7.3 ppm) of compound 3 ($CDCl_3$, 400 MHz).	21
Figure S18. APT spectrum of compound 3 ($CDCl_3$, 400 MHz).	22

Figure S19. HSQC spectrum of compound 3.	23
Figure S20. HMBC spectrum of compound 3.	24
Figure S21. HMBC spectrum expansion of compound 3.	25
Figure S22. HMBC spectrum expansion of compound 3.	26
Figure S23. EI- mass spectrum of compound 3.	27
Figure S24. IR spectrum of compound 3.	28
Figure S25. ^{1}H NMR spectrum of compound 4 ($CDCl_3$, 400 MHz).	29
Figure S26. ^{1}H NMR spectrum expansion (0.8- 2.2 ppm) of compound 4 ($CDCl_3$, 400 MHz).	30
Figure S27. ^{1}H NMR spectrum expansion (3.1- 5.4ppm) of compound 4 ($CDCl_3$, 400 MHz).	31
Figure S28. ^{13}C NMR spectrum of compound 4 ($CDCl_3$, 100 MHz).	32
Figure S29. HSQC spectrum of compound 4.	33
Figure S30. HSQC expansion of compound 4.	34
Figure S31. HSQC spectrum expansion of compound 4	35
Figure S32. HMBC spectrum of compound 4.	36
Figure S33. HMBC spectrum expansion (X =0.8 -2) of compound 4	37
Figure S34. HMBC spectrum expansion (X= 1.03- 2.05 ppm) of compound 4.	38
Figure S35. HMBC spectrum expansion (X= 5- 7.8 ppm) of compound 4.	39
Figure S36. HR-ESI-MS spectrum of compound 4.	40
Figure S37. EI- mass spectrum of compound 4.	41
Figure S38. IR spectrum of compound 4	42

Figure S39. ^{1}H NMR spectrum of compound 5 ($CDCl_3$, 400 MHz).	43
Figure S40. ^{1}H NMR spectrum expansion (0.8- 2.3ppm) of compound 5 ($CDCl_3$, 400 MHz).	44
Figure S41. ^{1}H NMR spectrum expansion (4.0- 7.5 ppm) of compound 5 ($CDCl_3$, 400 MHz).	45
Figure S42. APT spectrum of compound 5 ($CDCl_3$, 100 MHz).	46
Figure S43. HR-ESI-MS spectrum of compound 5.	47
Figure S44. EI-MS spectrum of compound 5.	48
Figure S45: IR spectrum of compound 5.	49
Figure S46. ^{1}H NMR spectrum of compound 6 ($CDCl_3$, 400 MHz).	50
Figure S47. ^{1}H NMR spectrum expansion (3.4- 4.2 ppm) of compound 6 ($CDCl_3$, 400 MHz**).**	51
Figure S48. APT spectrum of compound 6 ($CDCl_3$, 100 MHz).	52
Figure S49. HR-ESI-MS spectrum of compound 6.	53
Figure S50: IR spectrum of compound 6.	54
Figure S51:The proposed biosynthetic pathway of metabolite 2	55
Figure S52:The proposed biosynthetic pathway of metabolite 5	56
Figure S53. COX-1 and COX-2 minimum inhibitory dose causing 50% activity (IC_{50})	57
Figure S54. The effect of α-bisabolol, its metabolites and positive controls: catechin and epicatechin-3-gallate (ECG) on the SH-SY5Y cells viability	58
Table S1. ^{1}H NMR (400 MHz) spectroscopic data for α-bisabolol and compounds 2,3,4,5 and 6 (in $CDCl_3$)	59

Table S2. ^{13}C NMR (100 MHz) spectroscopic data for α-bisabolol and compounds 2,3,4,5 and 6 (in $CDCl_3$)	60
Table S3. The types of binding interactions of the most selective COX-2 inhibitors compound 4 and 5 in COX-1 compared to indomethacin a reference non-selective COX inhibitor	61, 62
Table S4. The COX-2 inhibition (IC_{50} μM), selectivity index (SI)[a], docking scores[b] and type of binding interactions of bisabolol, its metabolites (2-6) and Celecoxib (reference compound).	63
Table S5. Results of acetylcholine esterase inhibitory assay represented by IC50 (μM)	64
Table S6. AChE Inhibition (IC_{50} μM), Docking Scores, and Binding Interactions of the Tested Isolated Compounds and the Reference Compound (Galanthamine)).	65
Table S7. Calculated parameters of Lipinski's rule of five for α-bisabolol and metabolites 2-6.	66
Table S8. Predicted ADMET data for α-bisabolol and metabolites 2-6.	66

NMR data of (-)-α-bisabolol:

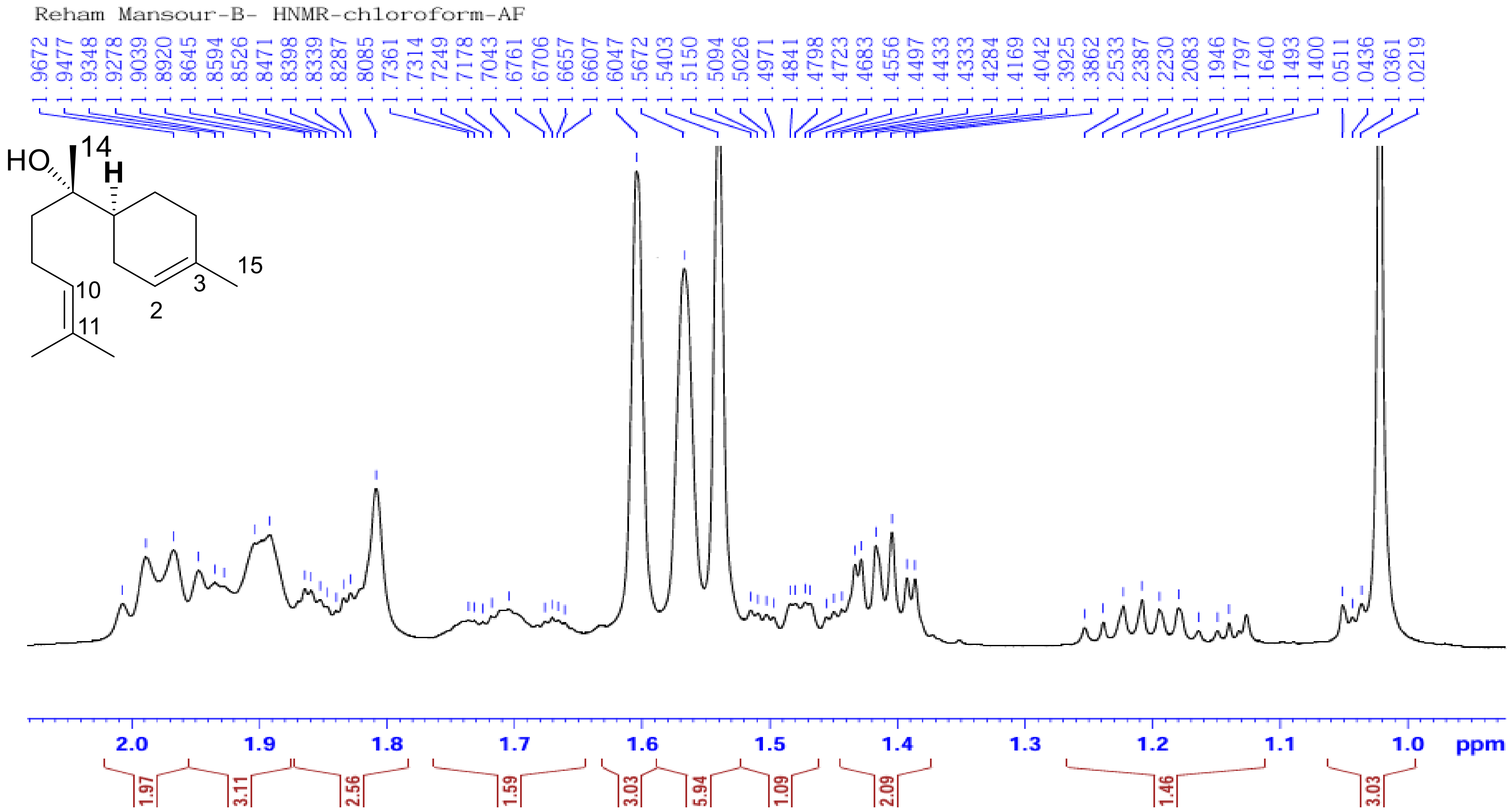

Figure S1. ^{1}H NMR spectrum of compound 1 ((-)-α-bisabolol) ($CDCl_3$, 400 MHz).

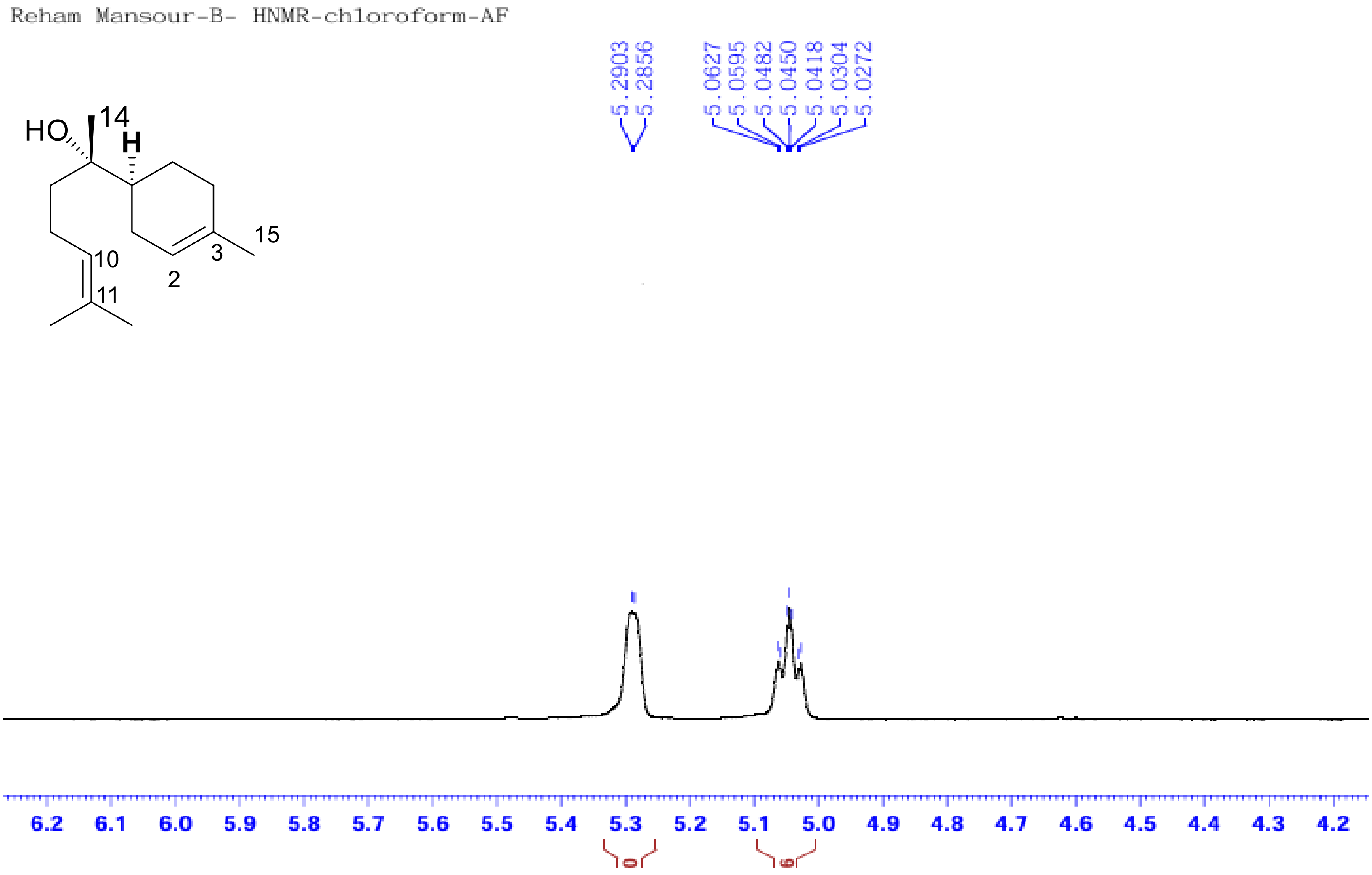

Figure S2. ^{1}H NMR spectrum expansion of compound 1 ((-)-α-bisabolol) ($CDCl_3$, 400 MHz).

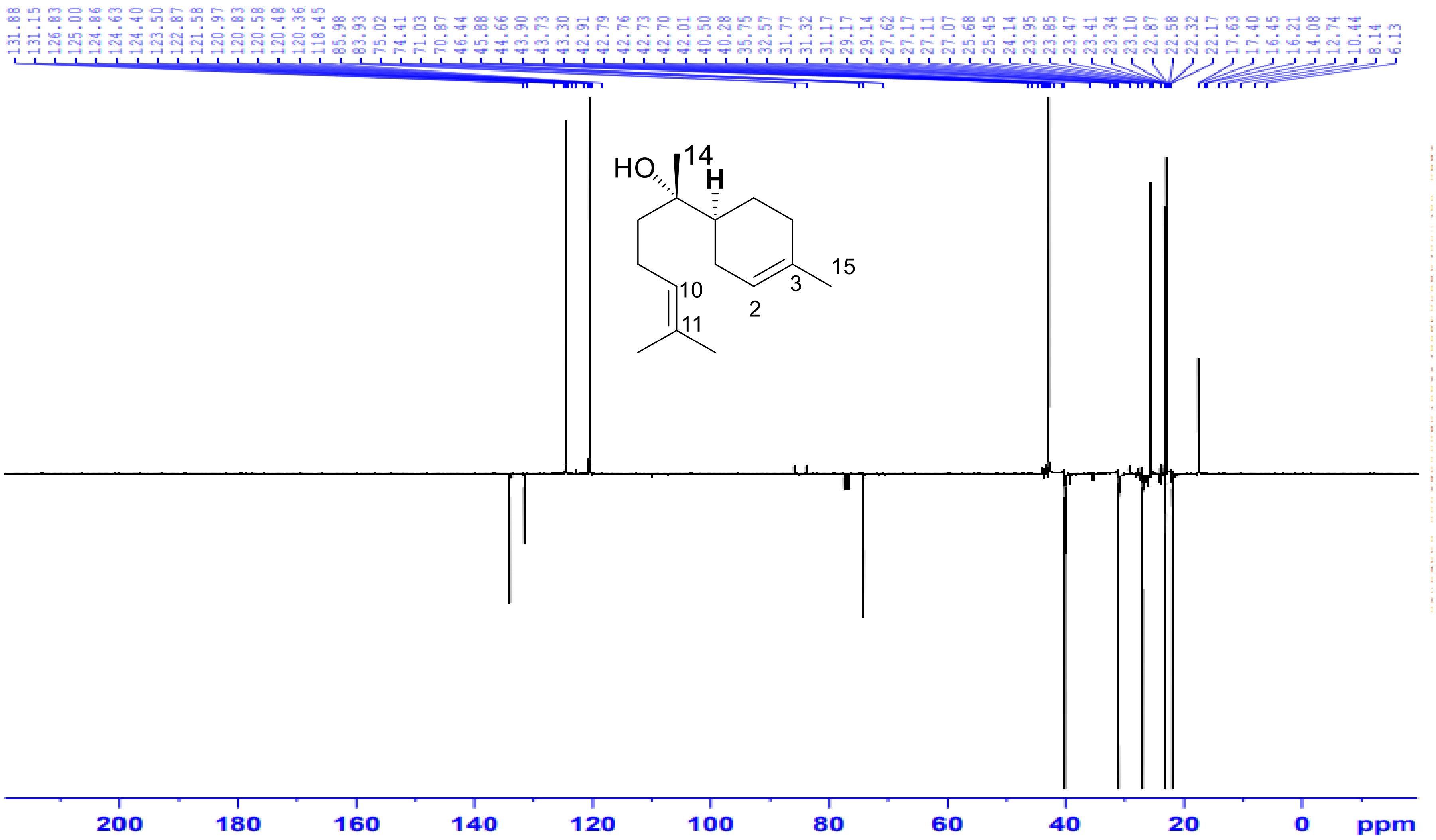

Figure S3. APT spectrum of compound 1 ((-)-α-bisabolol) ($CDCl_3$, 100 MHz).

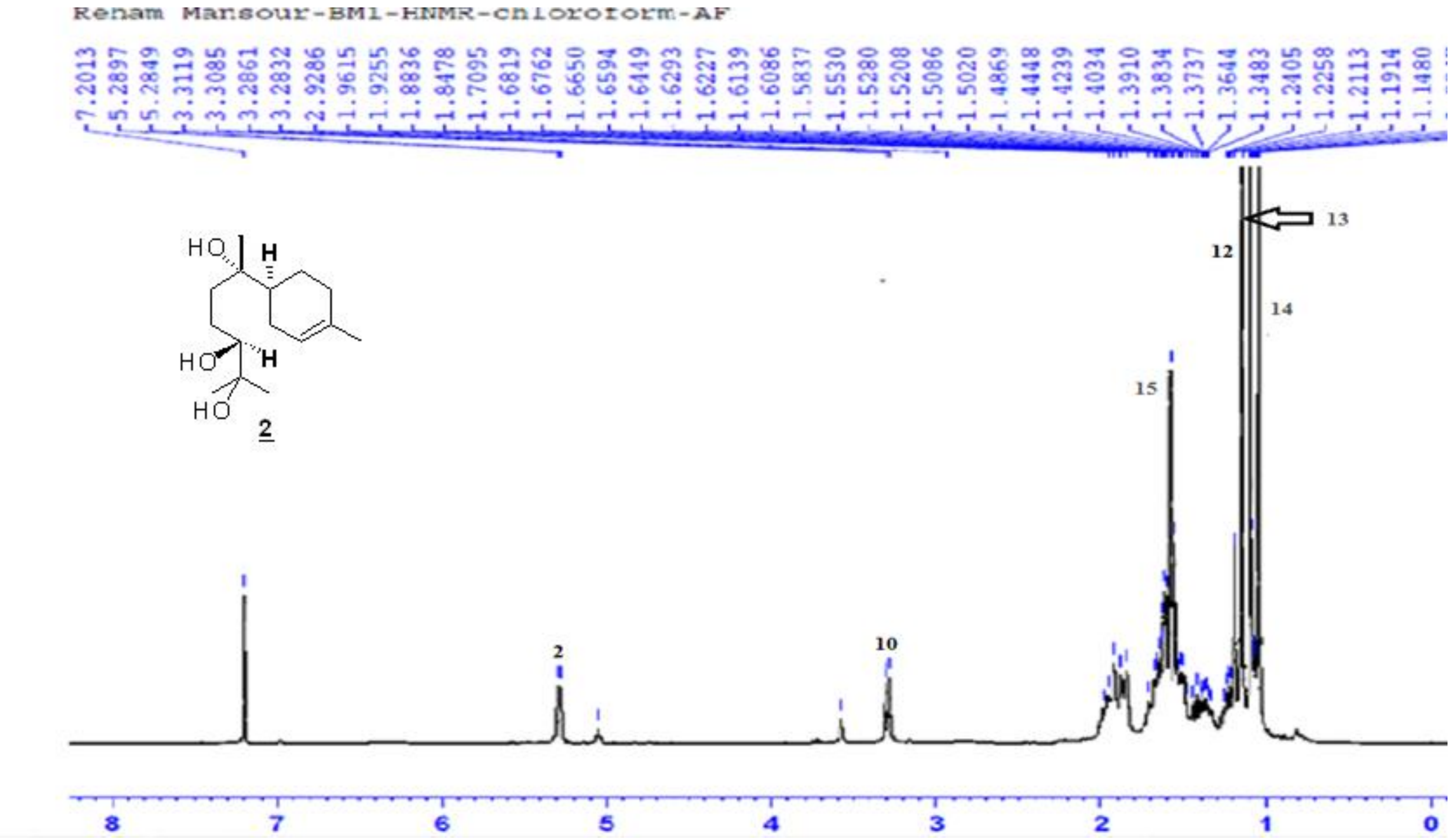

Figure S4. ^{1}H NMR spectrum of compound 2 ($CDCl_3$, 400 MHz).

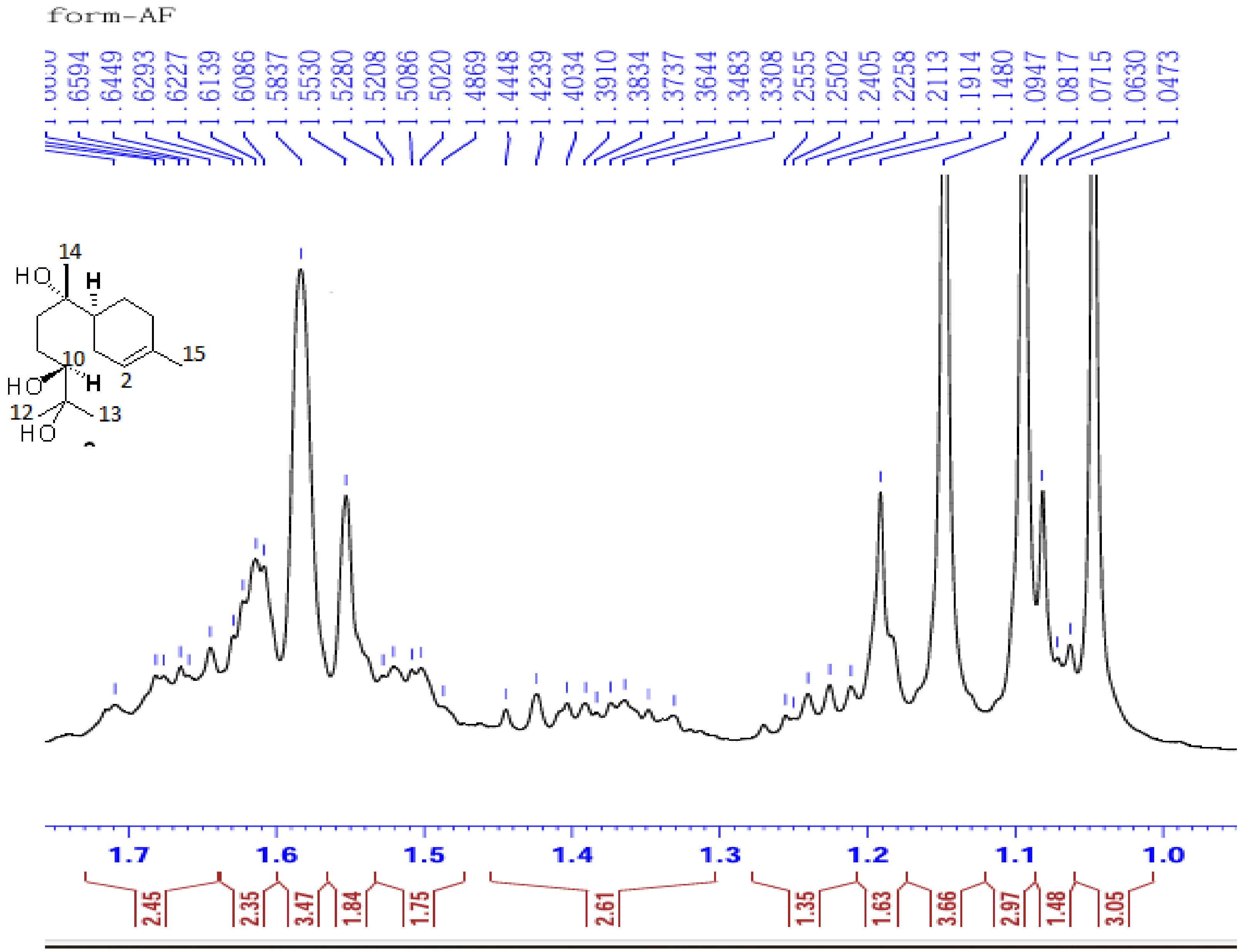

Figure S5. ^{1}H NMR spectrum expansion (1 – 1.7 ppm) of compound 2 ($CDCl_3$, 400 MHz).

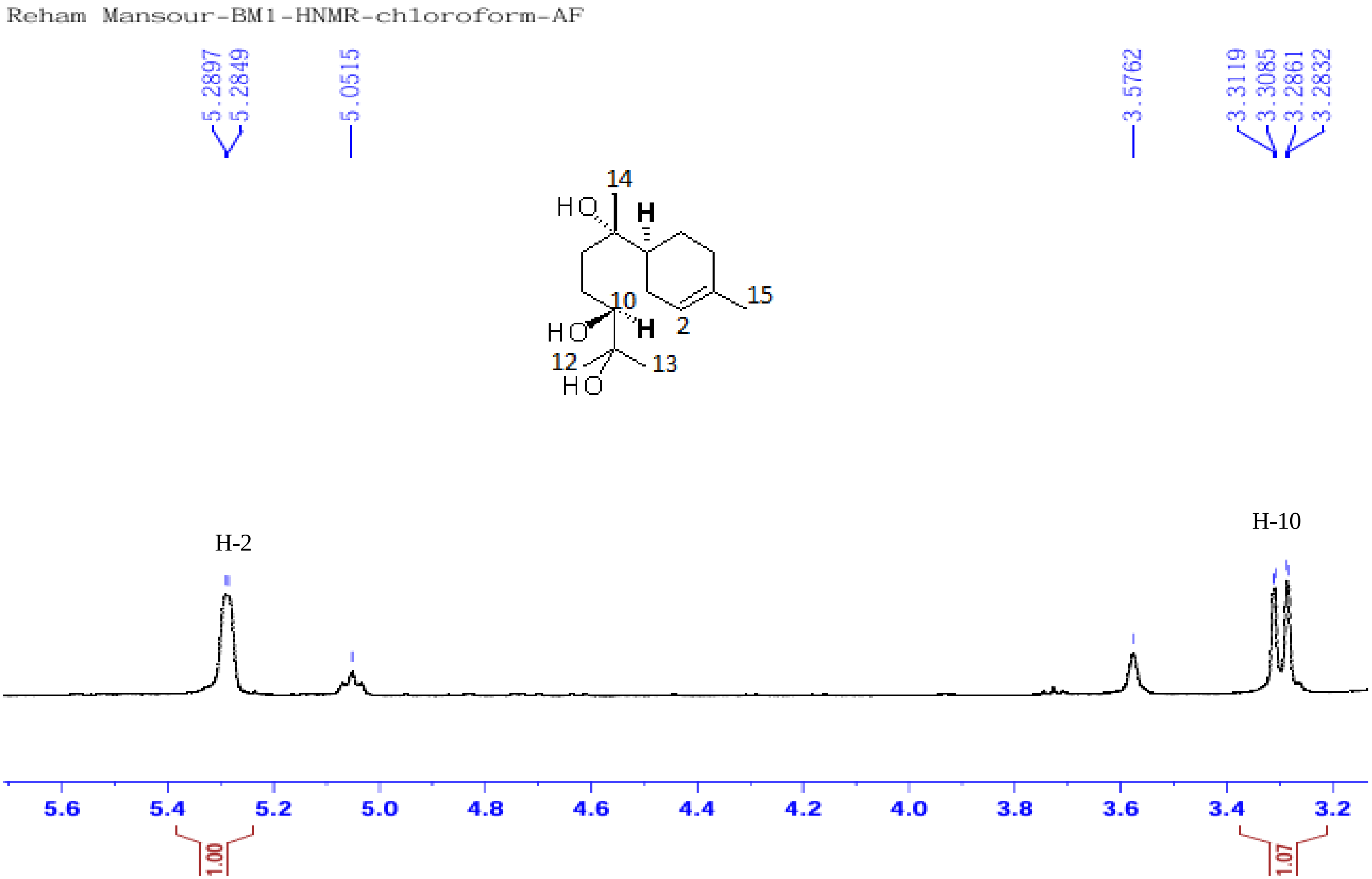

Figure S6. ^{1}H NMR spectrum expansion (3.00 - 5.6 ppm) of compound 2 ($CDCl_3$, 400 MHz).

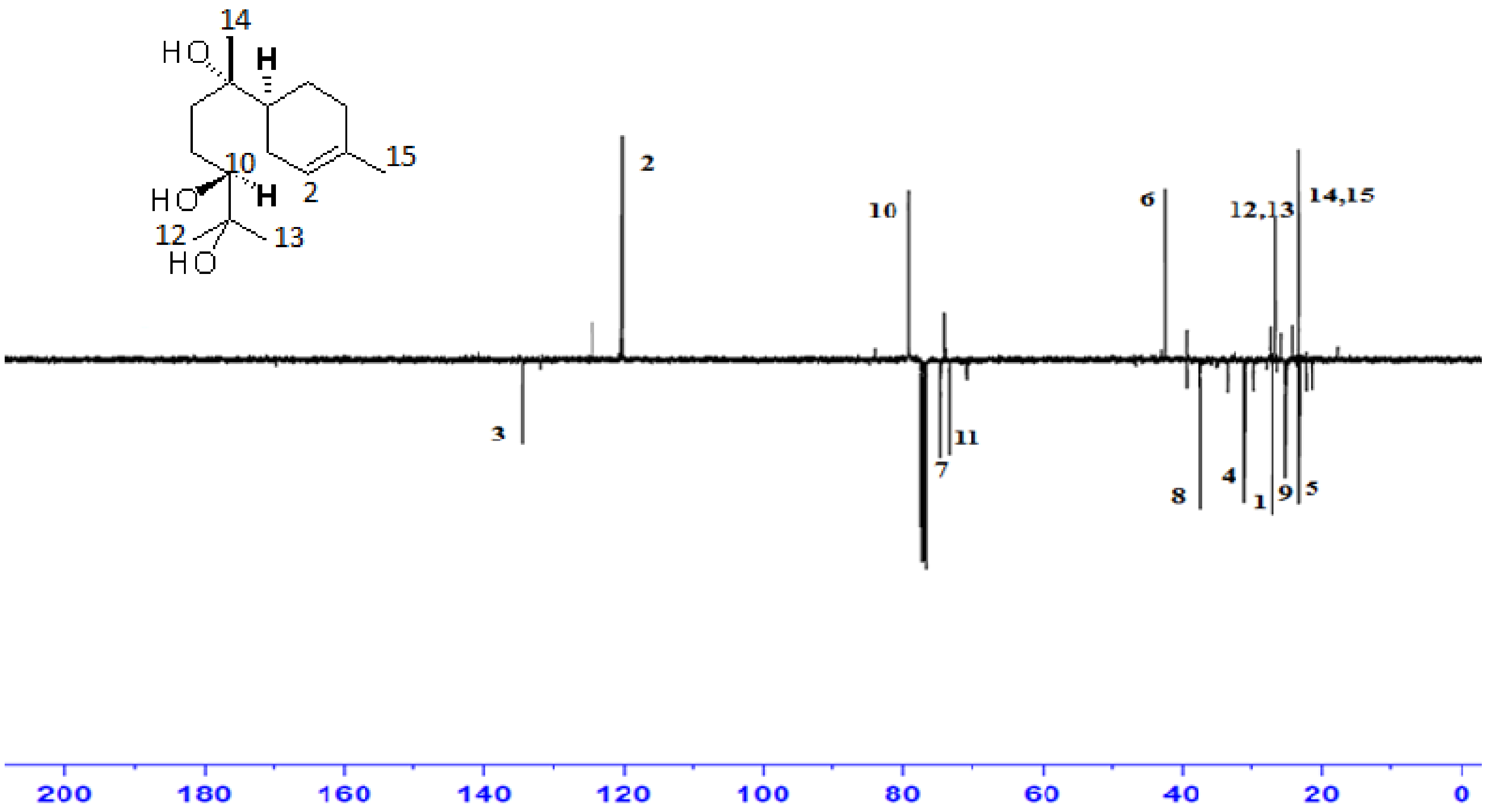

Figure S7. APT spectrum of compound 2 ($CDCl_3$, 100 MHz).

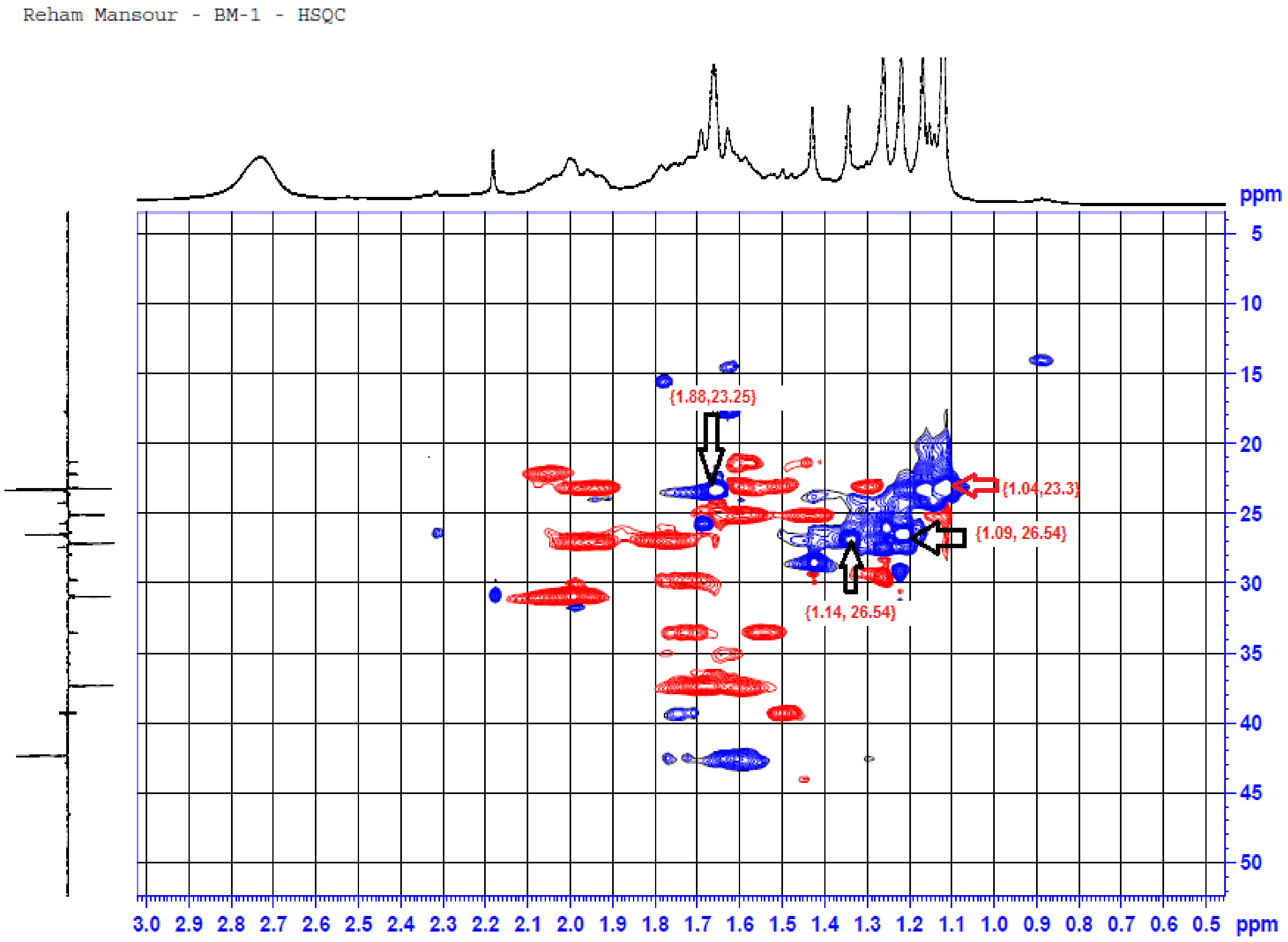

Figure S8. HSQC spectrum expansion of compound 2.

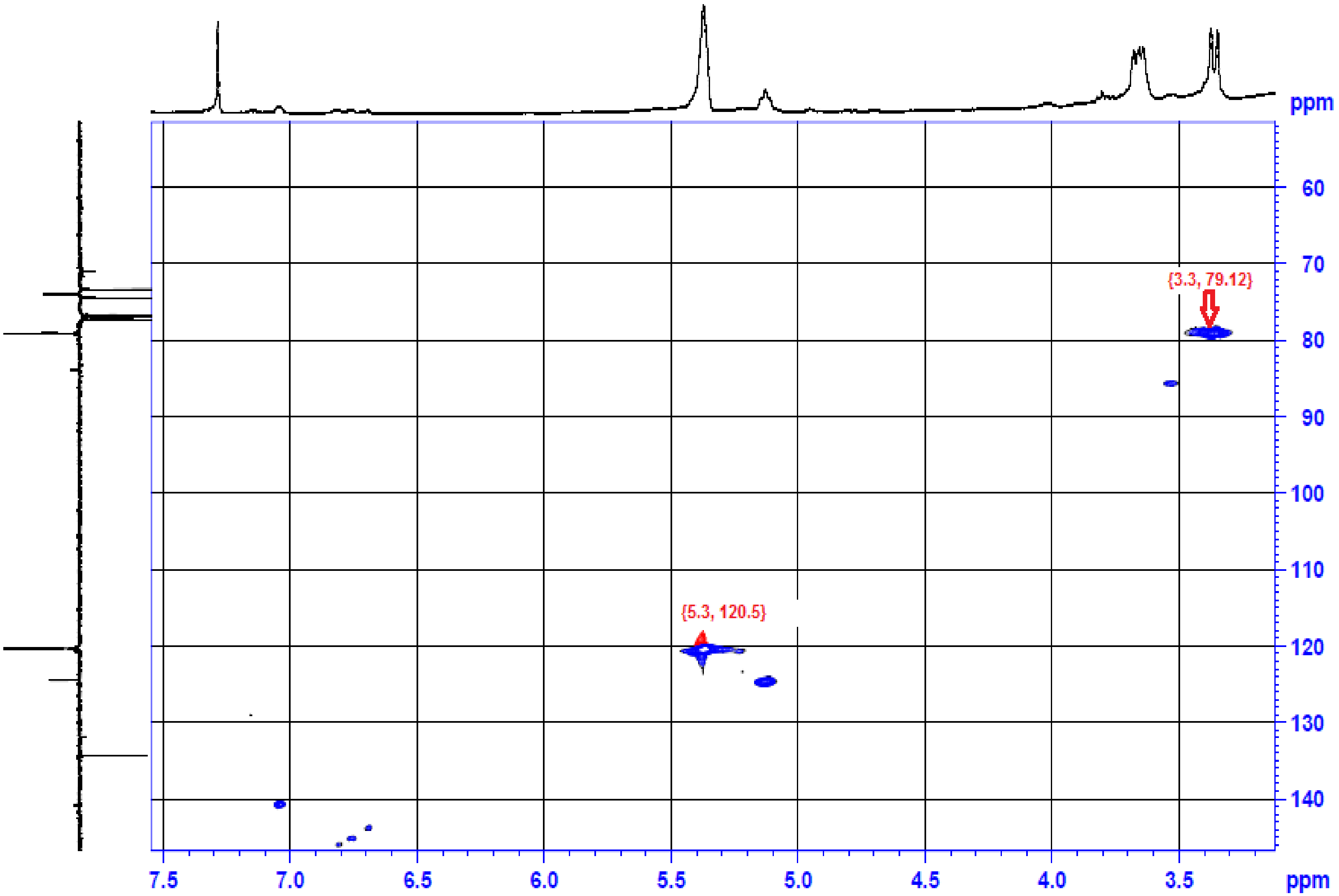

Figure S9. HSQC spectrum expansion of compound 2**.**

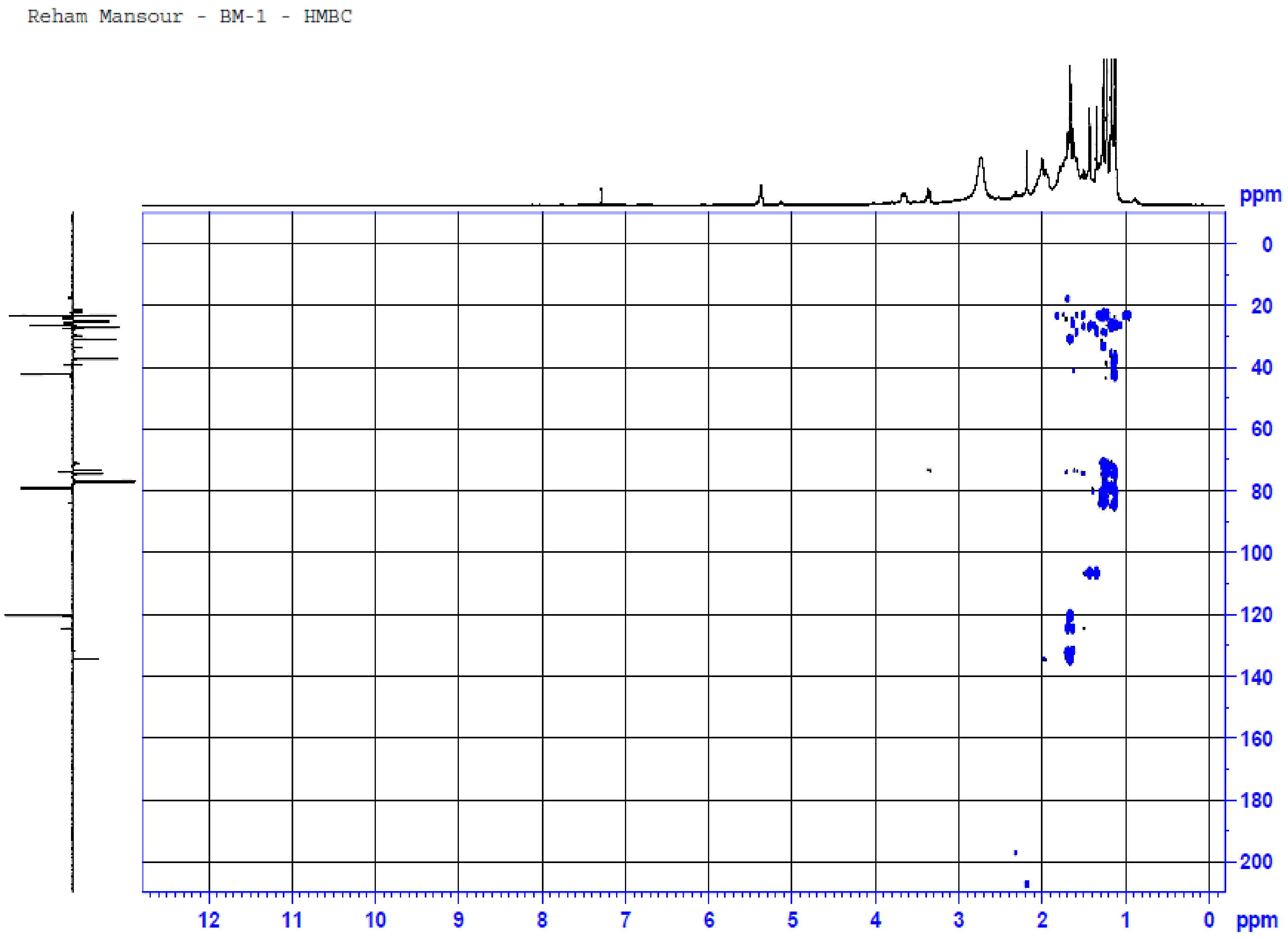

Figure S10. HMBC spectrum of compound 2 ($CDCl_3$, 100 MHz).

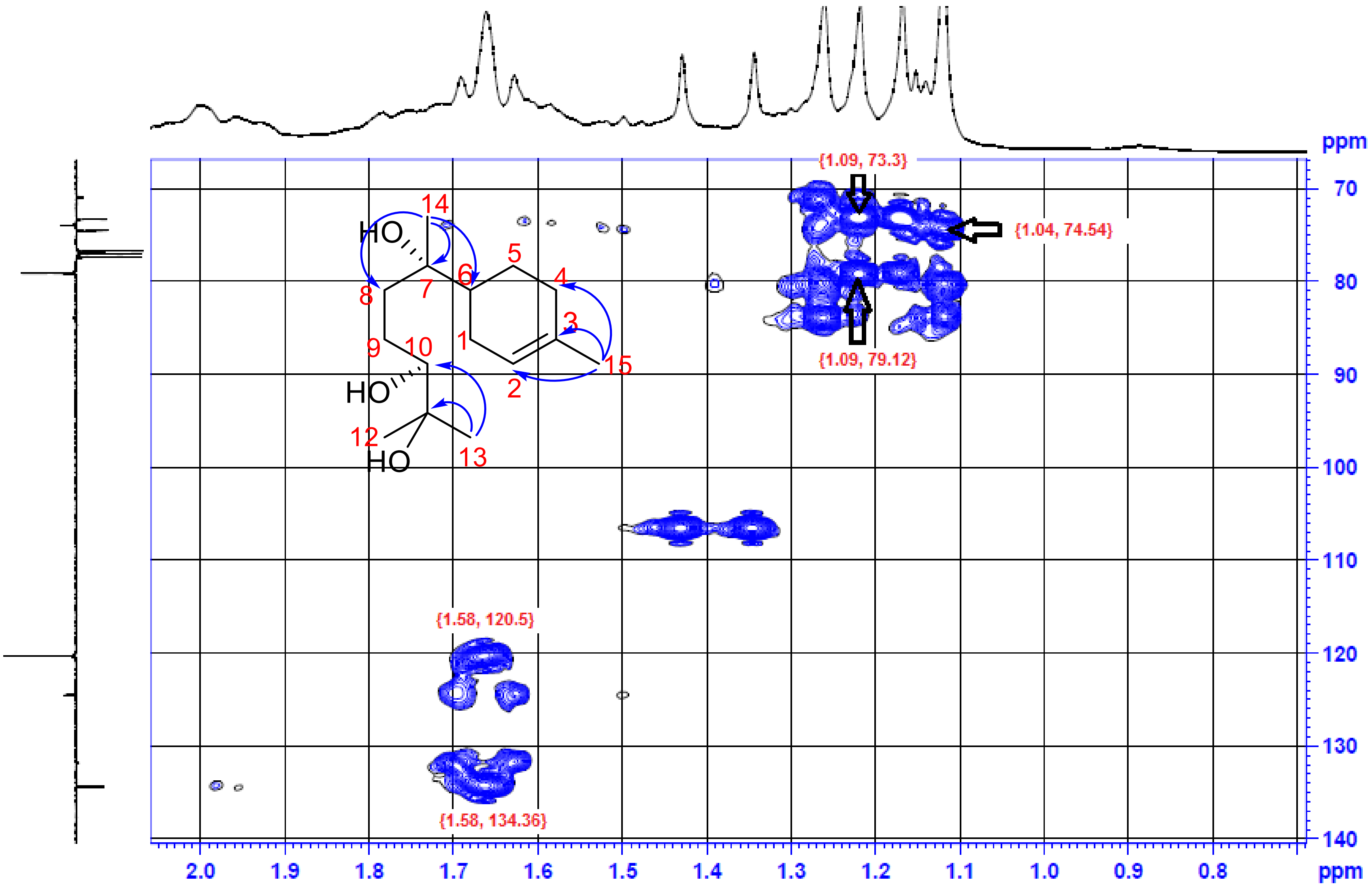

Figure S11. HMBC spectrum expansion of compound 2 ($CDCl_3$, 100 MHz).

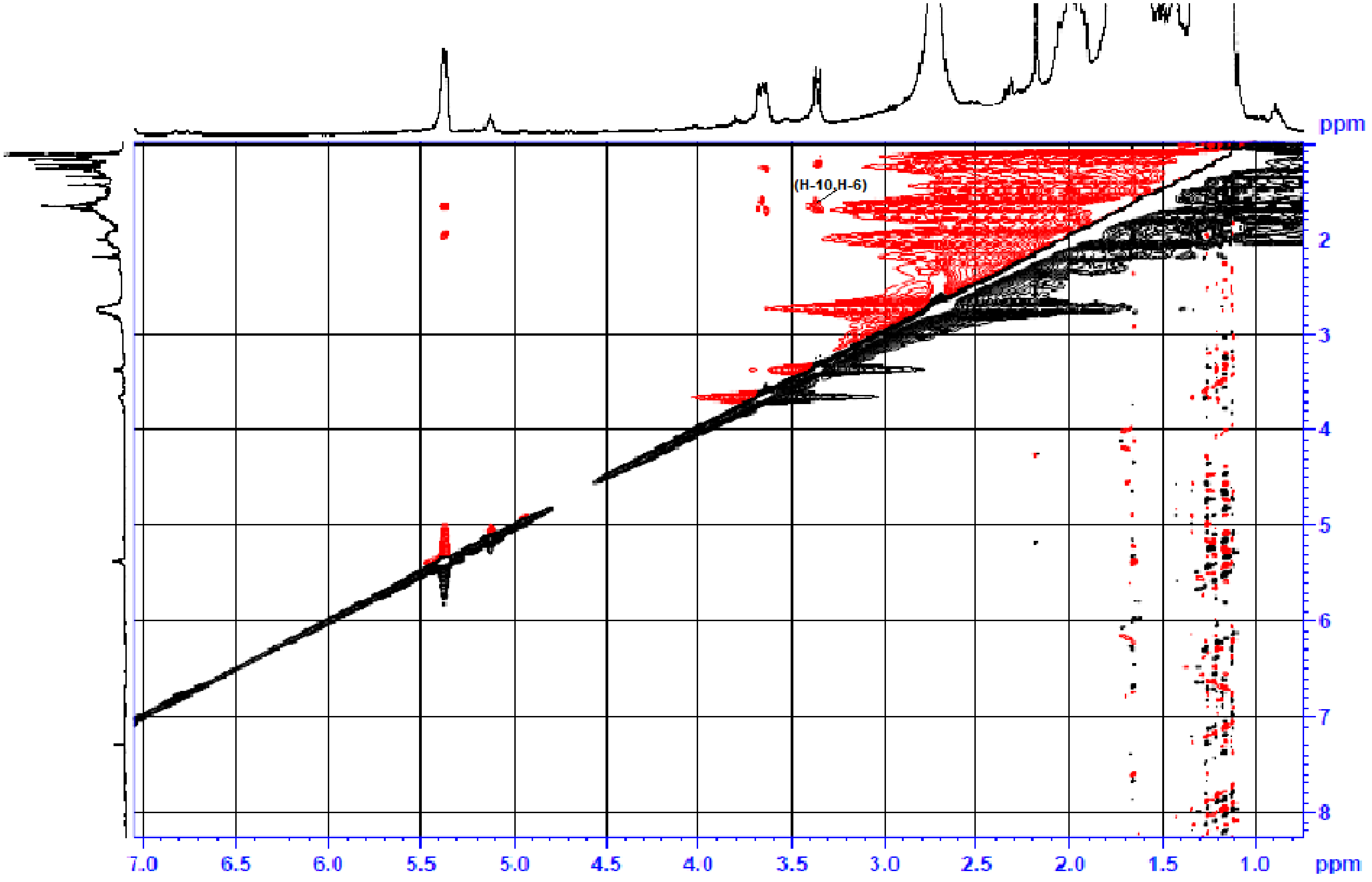

Figure S12. NOESY spectrum of compound 2 ($CDCl_3$, 100 MHz).

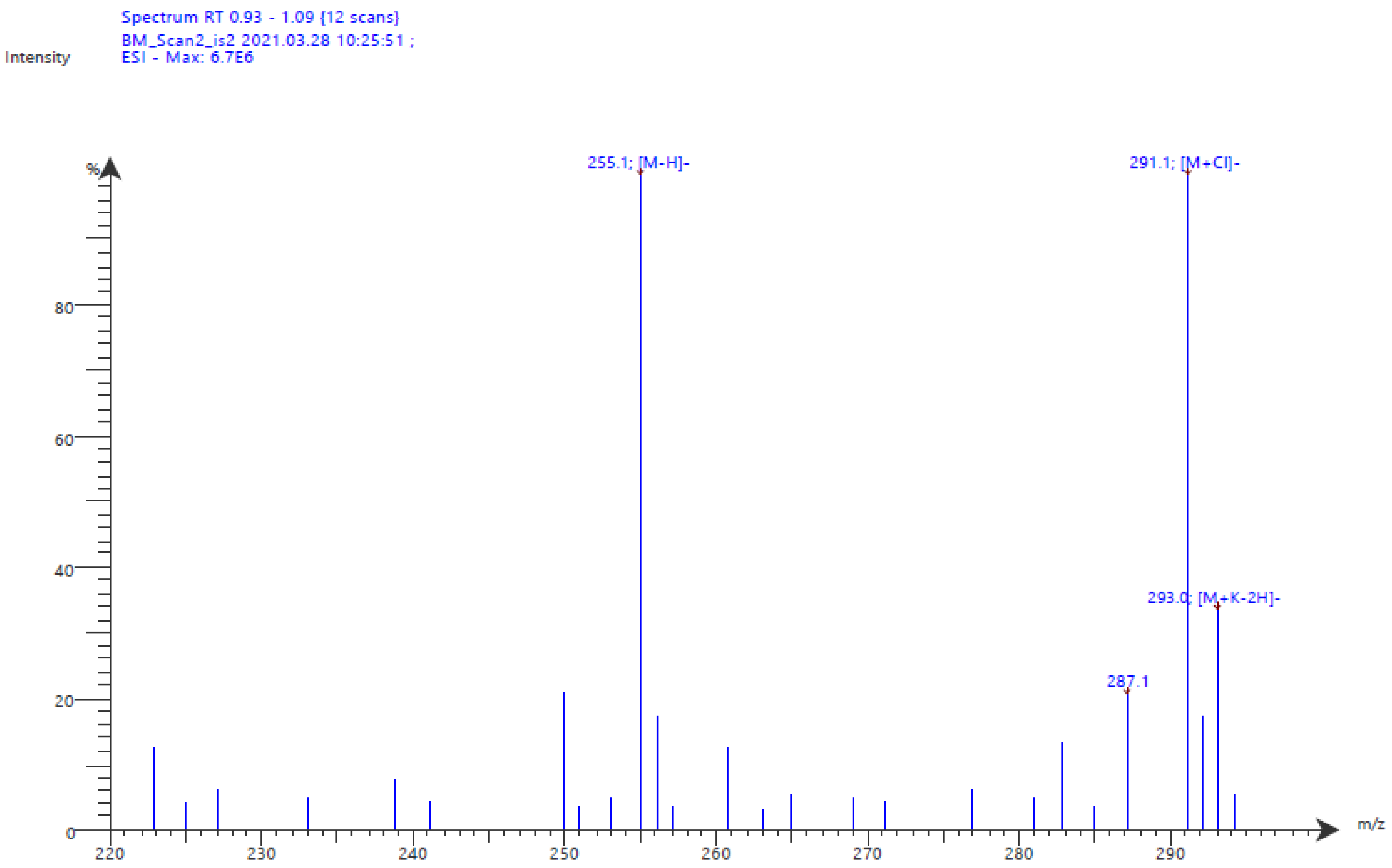

Figure S13. ESI-MS spectrum of compound 2.

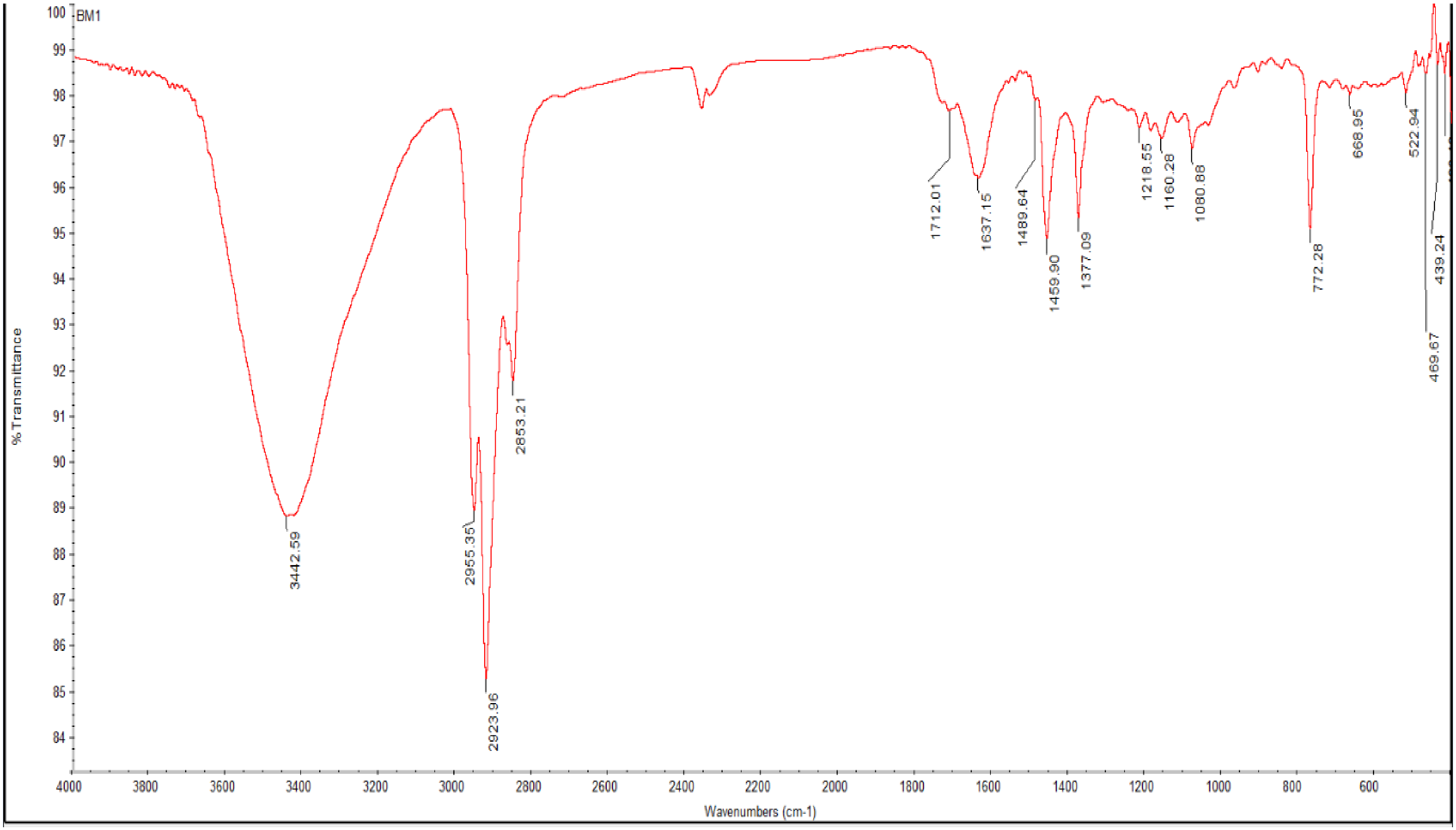

Figure S14. IR spectrum of compound 2.

Spectra of compound 3:

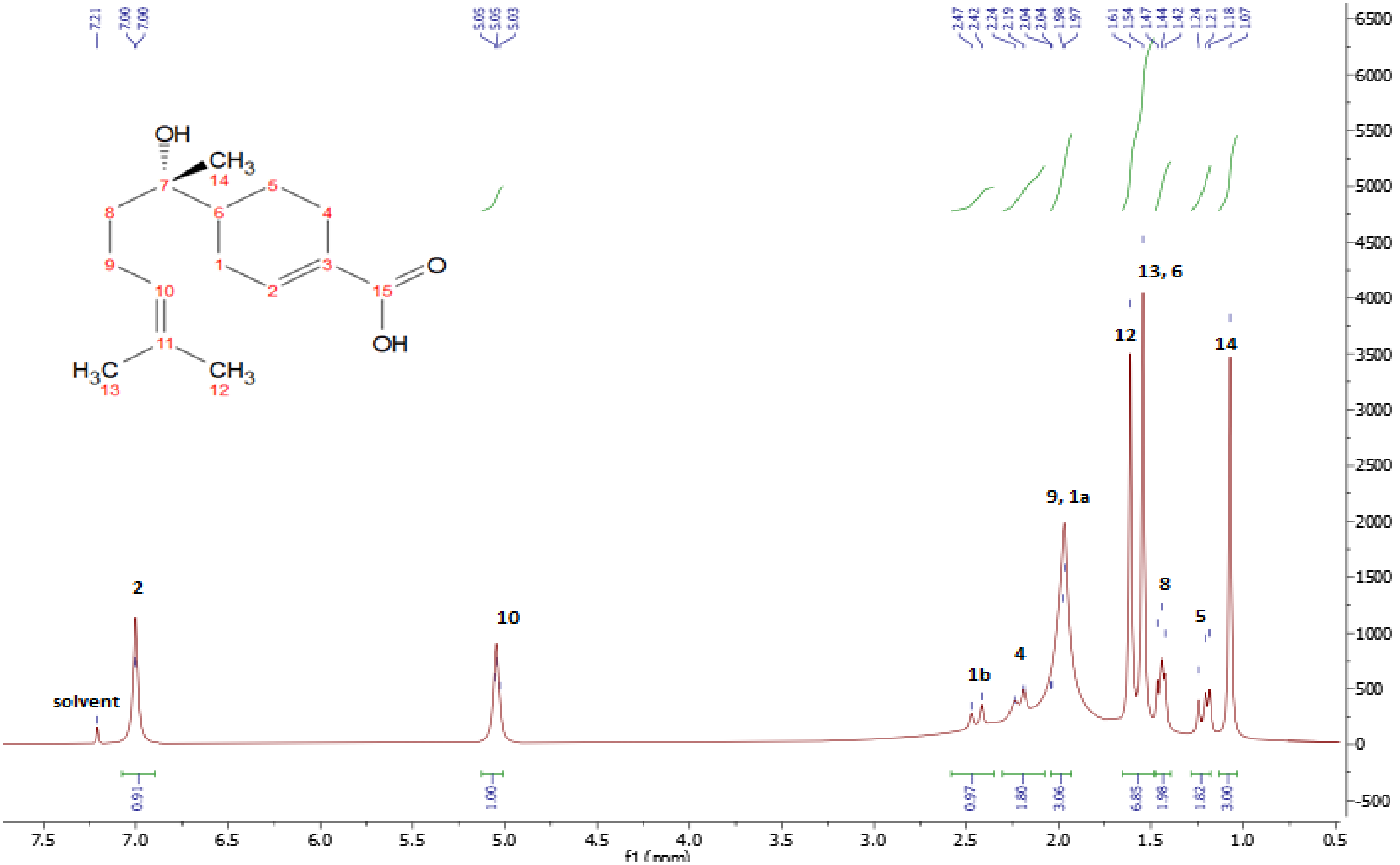

Figure S15. ^{1}H NMR spectrum of compound 3 ($CDCl_3$, 400 MHz).

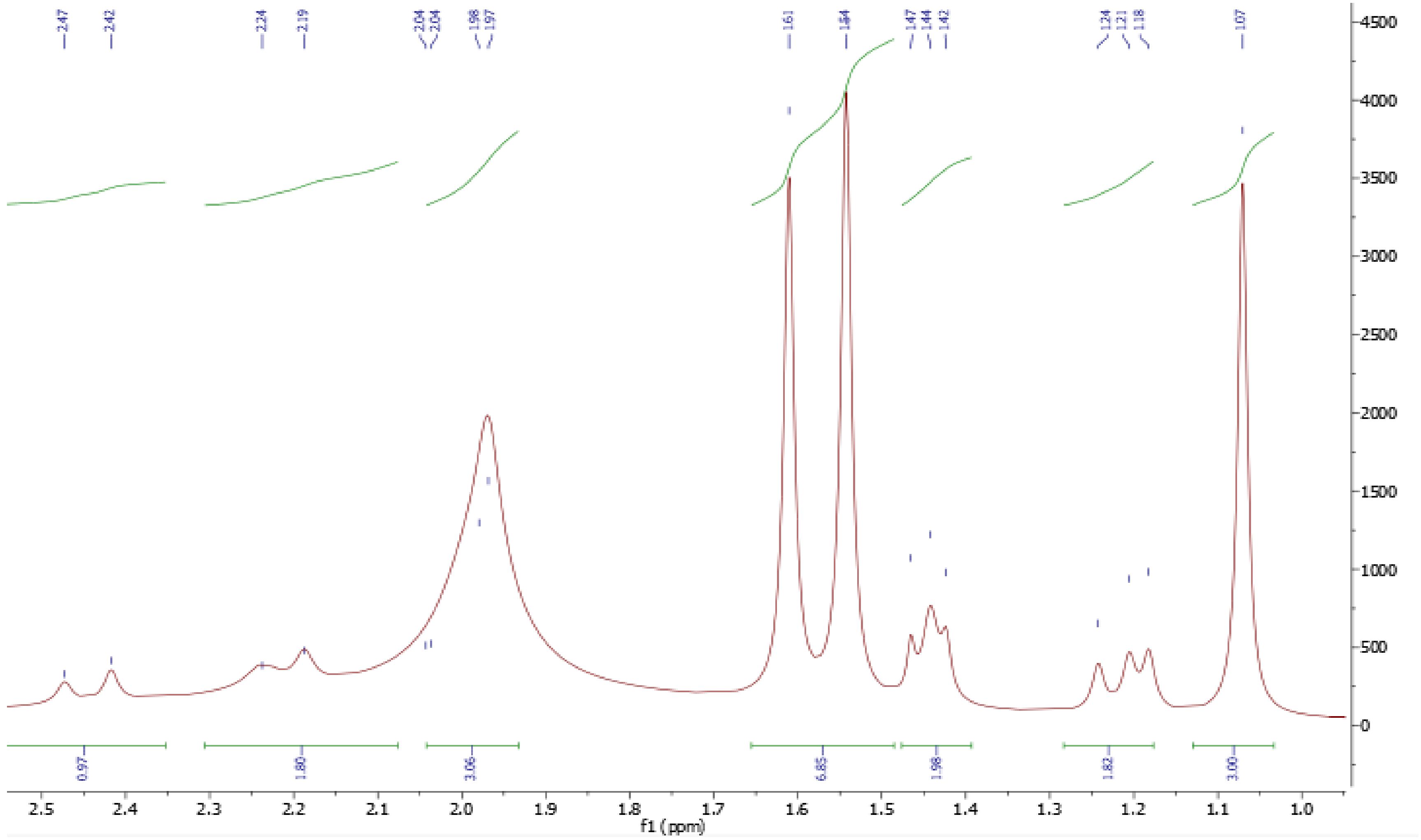

Figure S16. ^{1}H NMR spectrum expansion (1-2.5 ppm) of compound 3 ($CDCl_3$, 400 MHz).

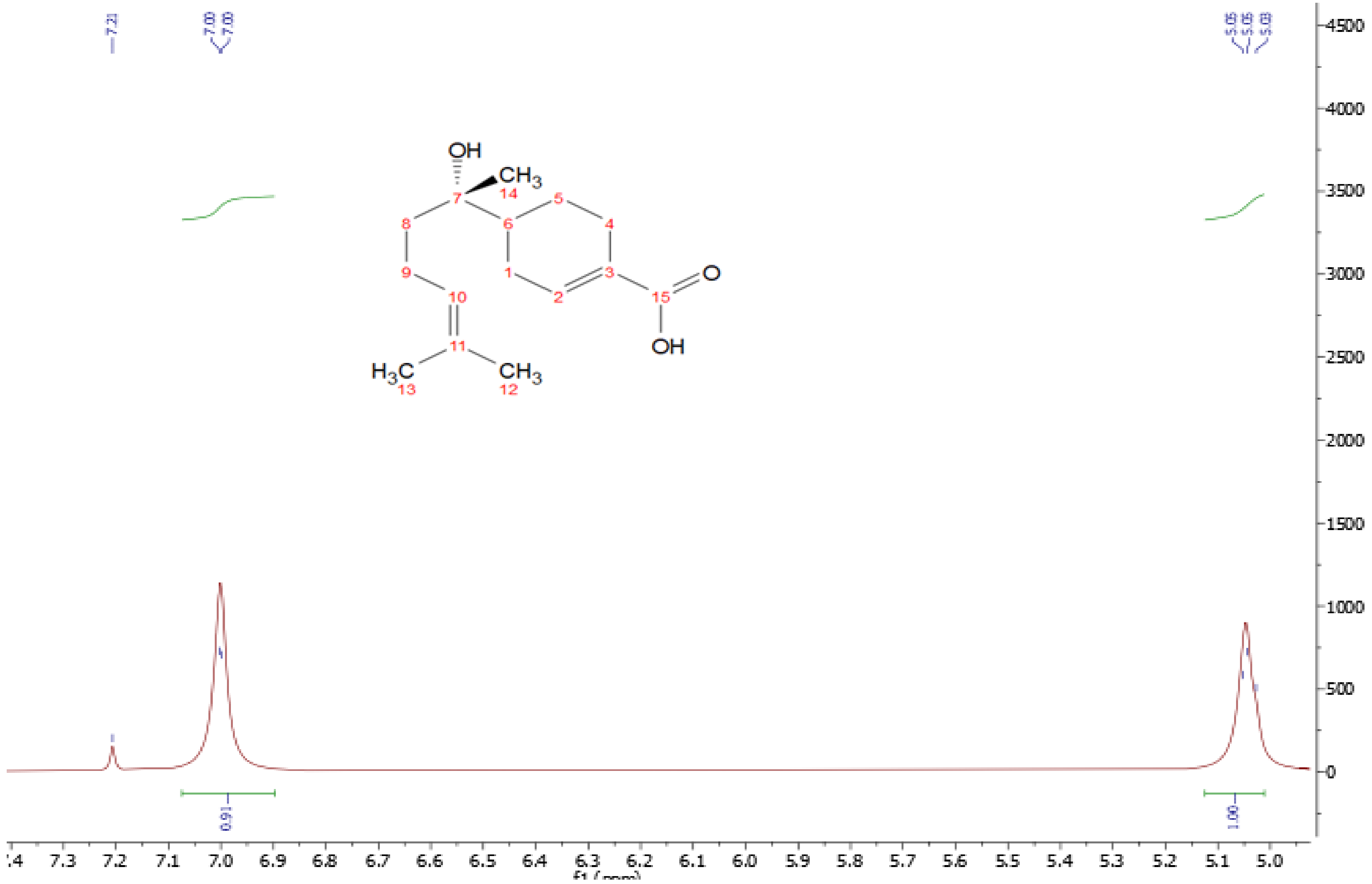

Figure S17. ^{1}H NMR spectrum expansion (5-7.3 ppm) of compound 3 ($CDCl_3$, 400 MHz).

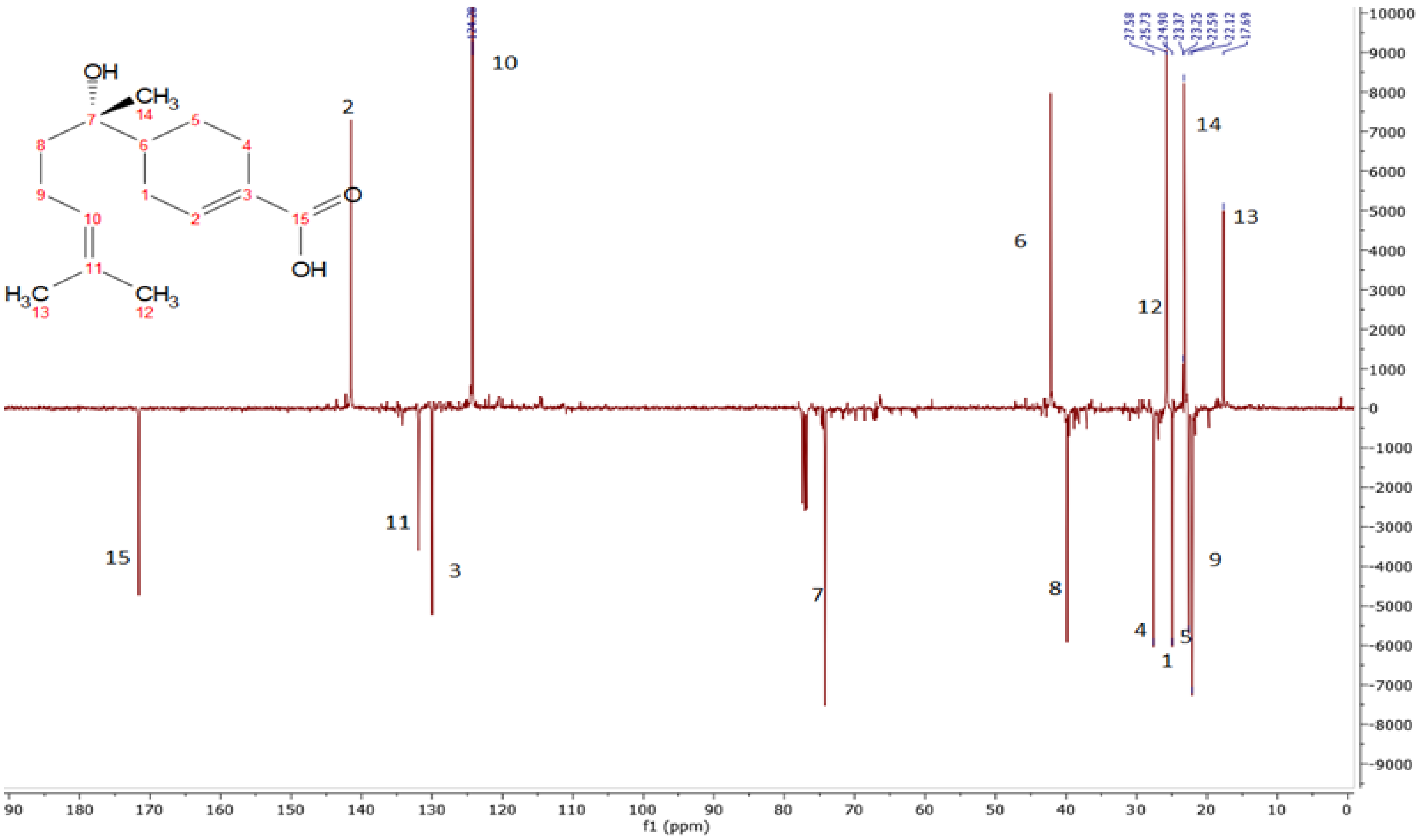

Figure S18. APT spectrum of compound 3 ($CDCl_3$, 100 MHz).

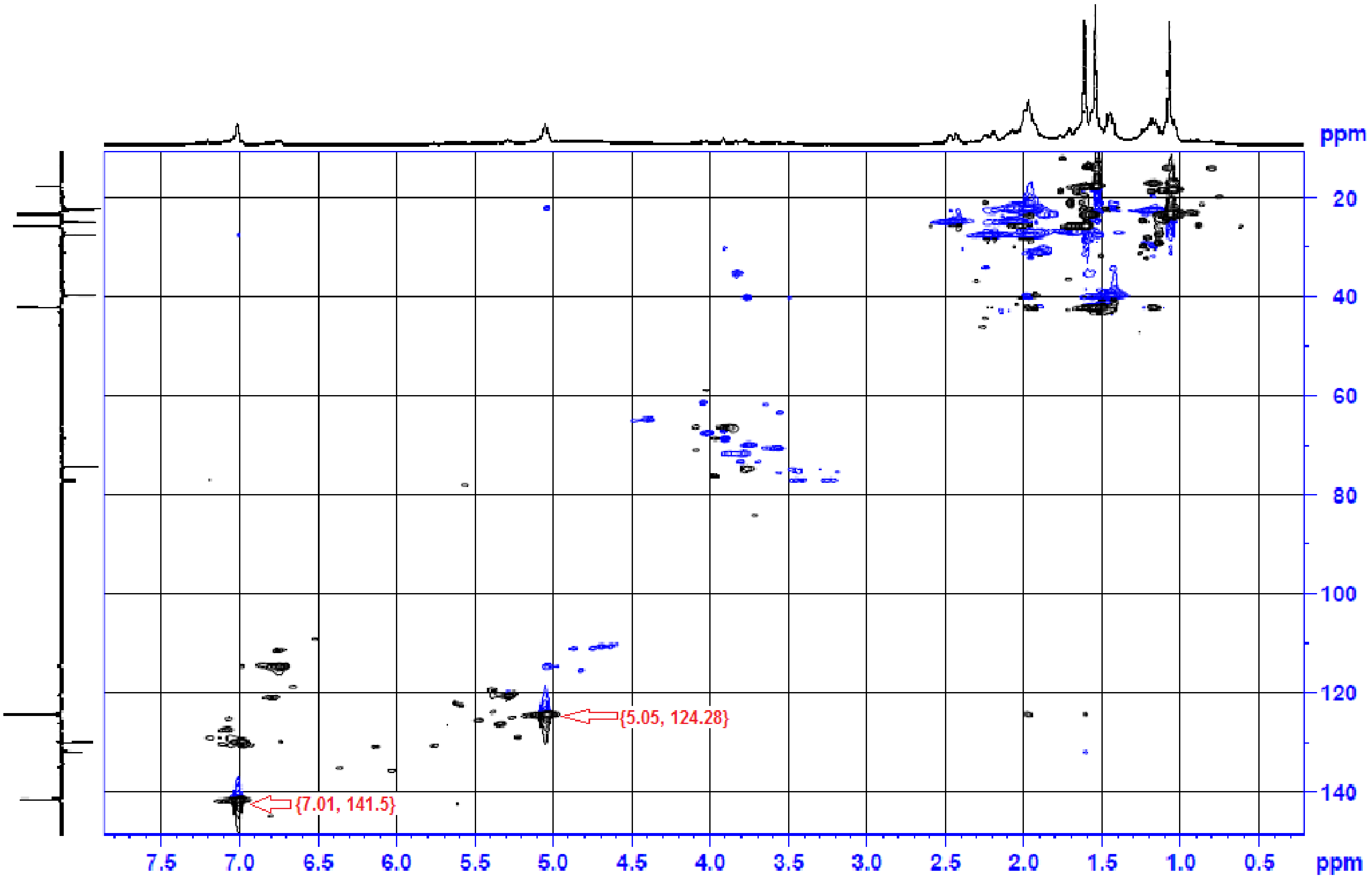

Figure S19. HSQC spectrum of compound 3.

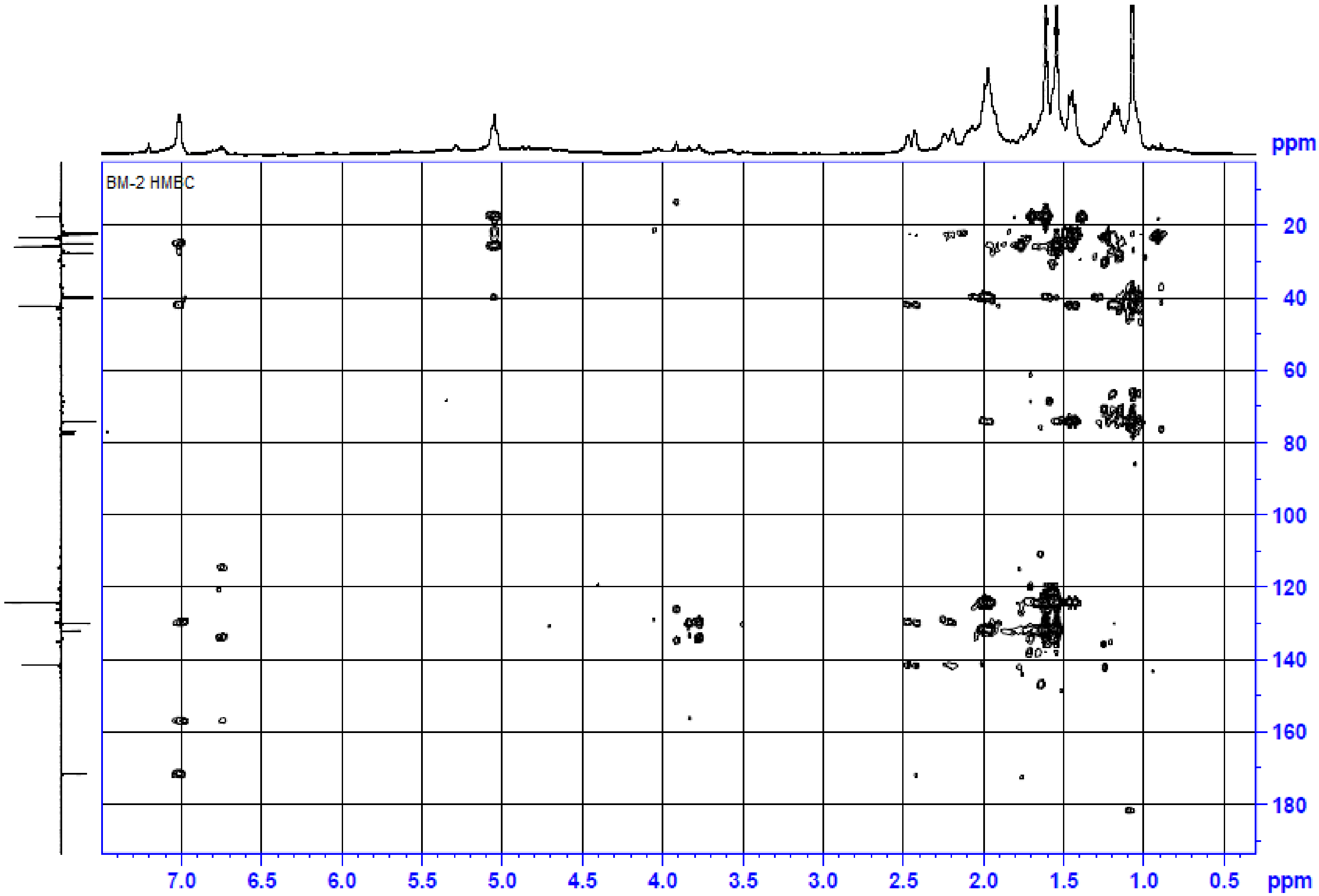

Figure S20. HMBC spectrum of compound **3**.

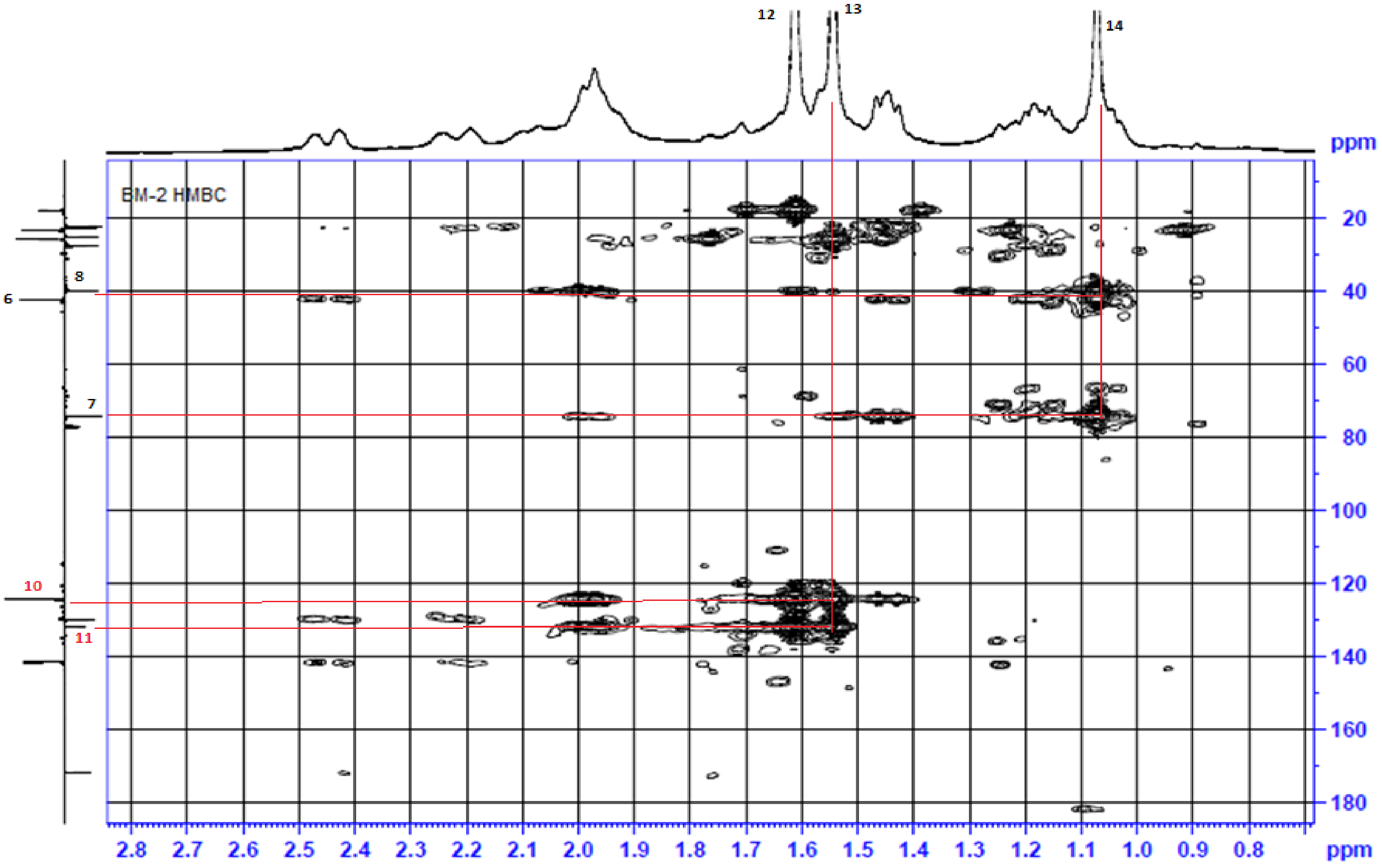

Figure S21. HMBC spectrum expansion of compound **3**.

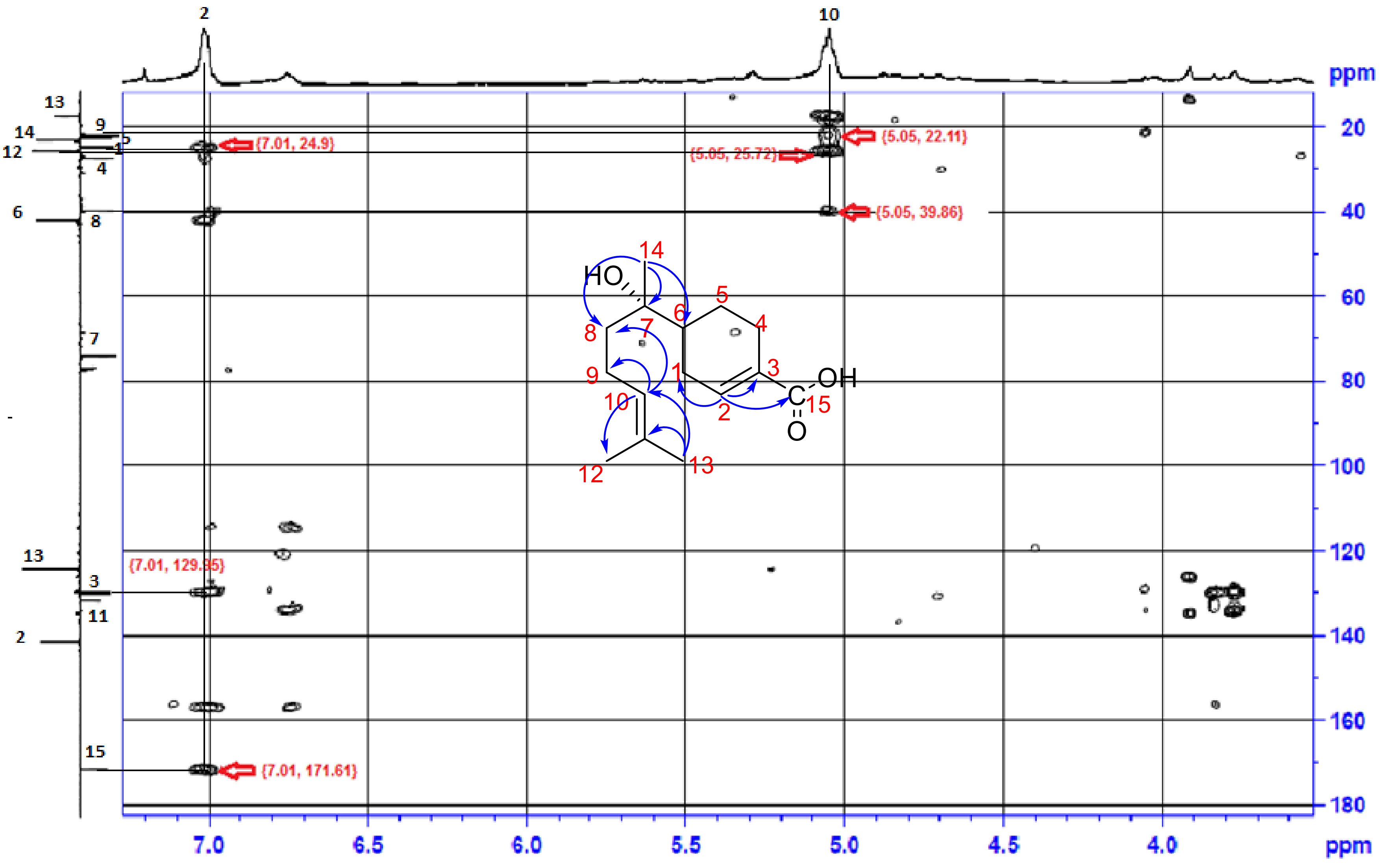

Figure S22. HMBC spectrum expansion of compound 3.

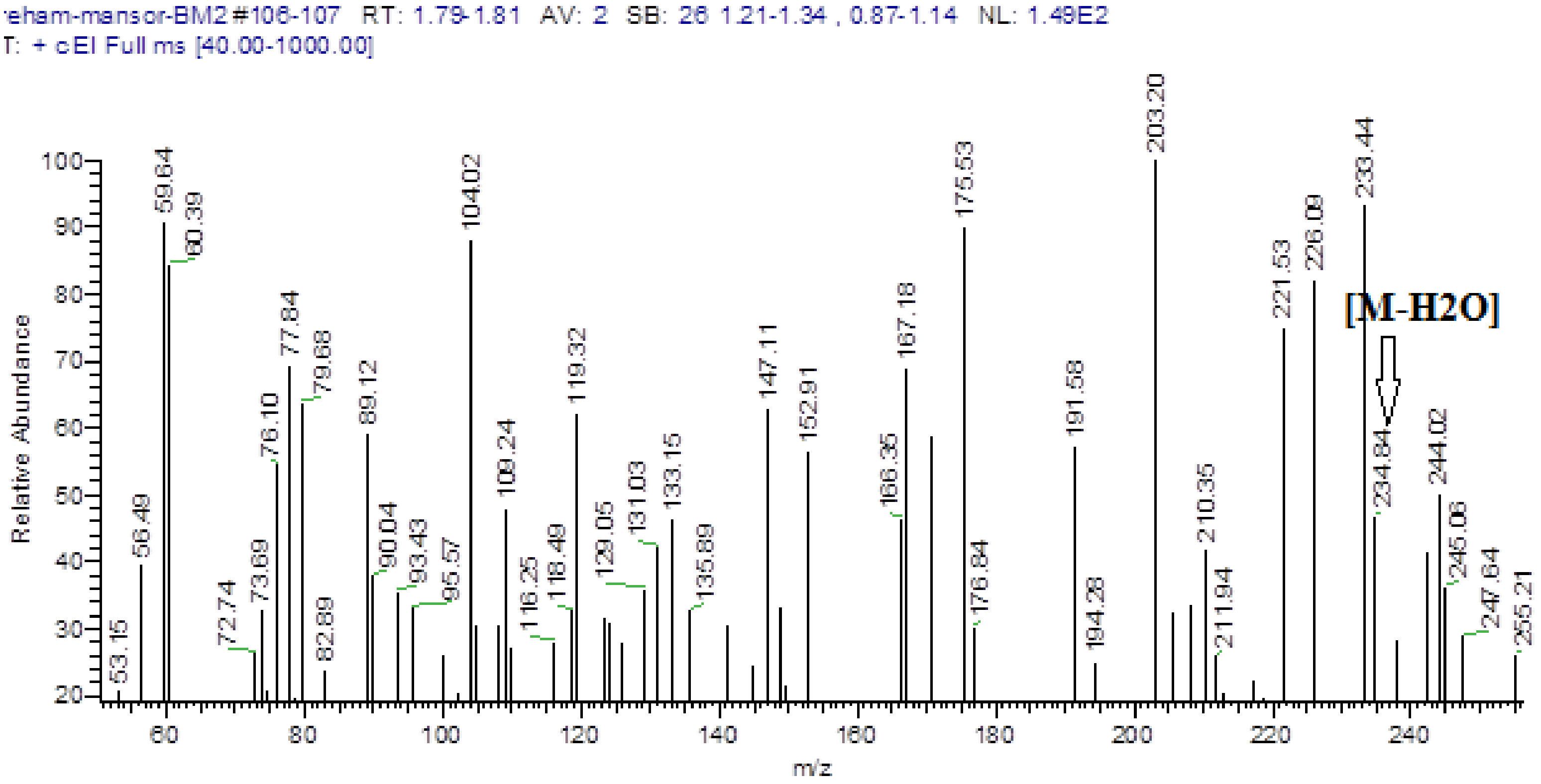

Figure S23. EI-MS spectrum of compound 3.

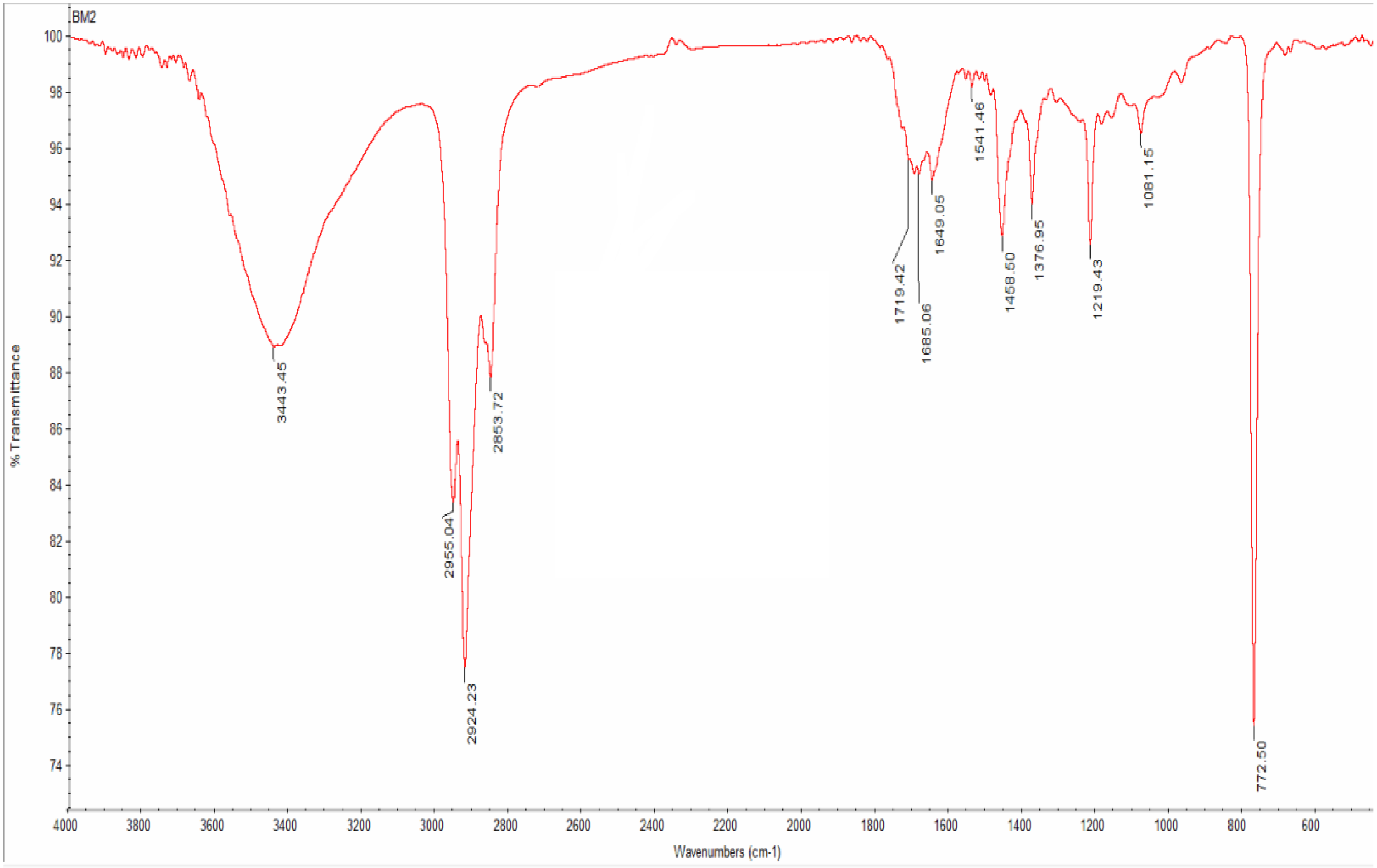

Figure S24. IR spectrum of compound 3.

Spectra of compound 4:

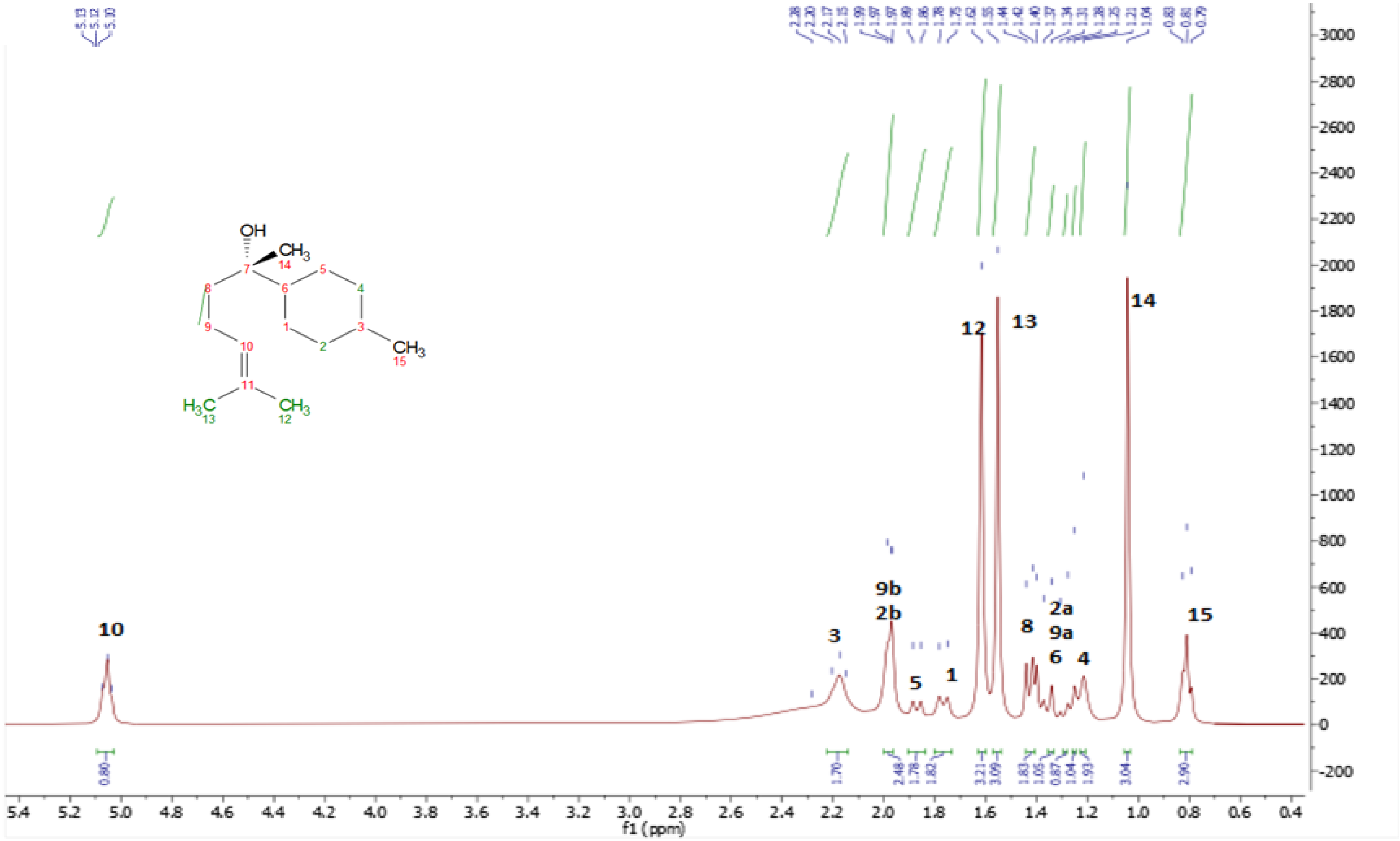

Figure S25. ^{1}H NMR spectrum of compound 4 ($CDCl_3$, 400 MHz).

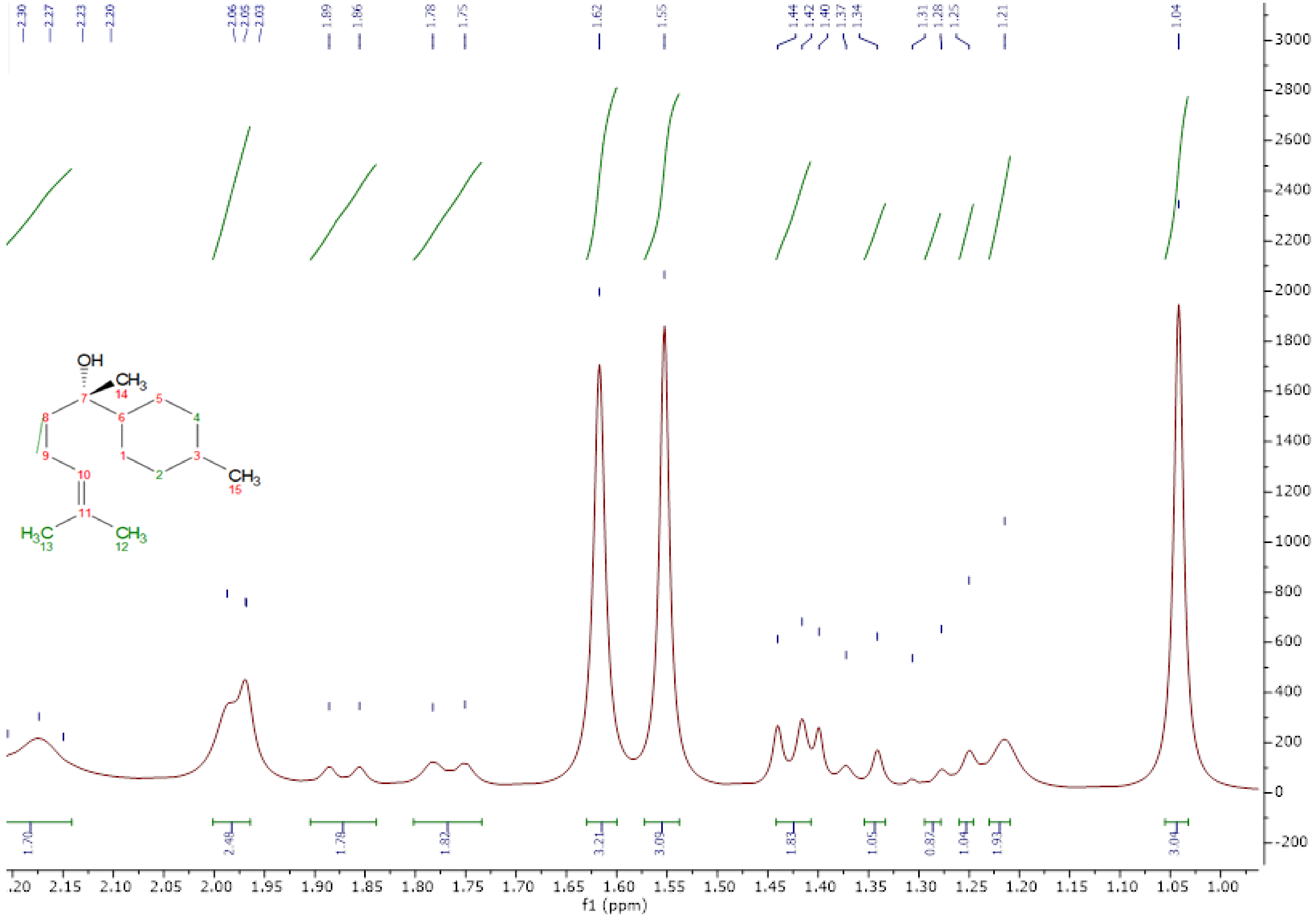

Figure S26. ^{1}H NMR spectrum expansion (0.8- 2.2 ppm) of compound 4 ($CDCl_3$, 400 MHz).

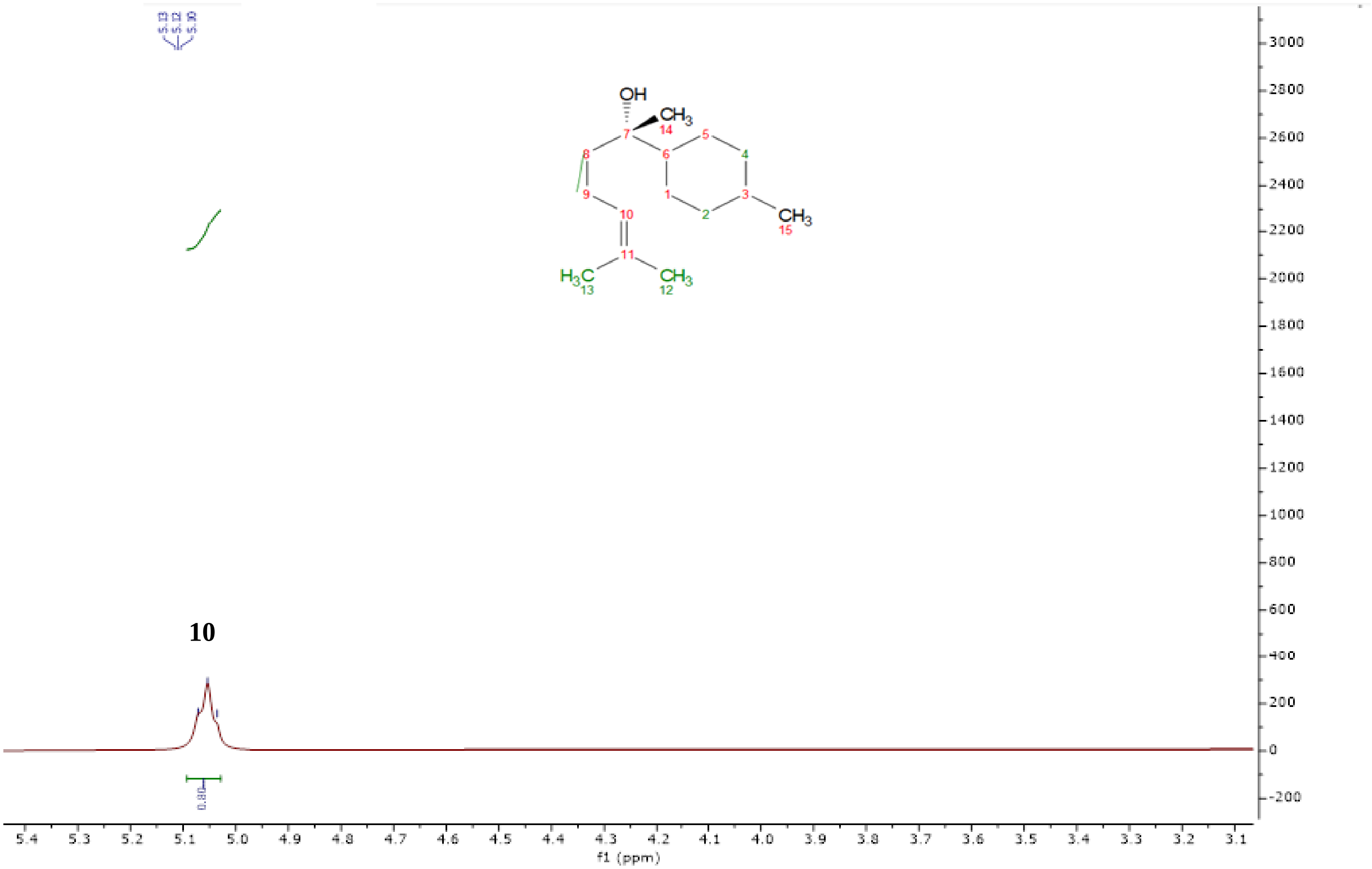

Figure S27. ^{1}H NMR spectrum expansion (3.1- 5.4ppm) of compound 4 ($CDCl_3$, 400 MHz).

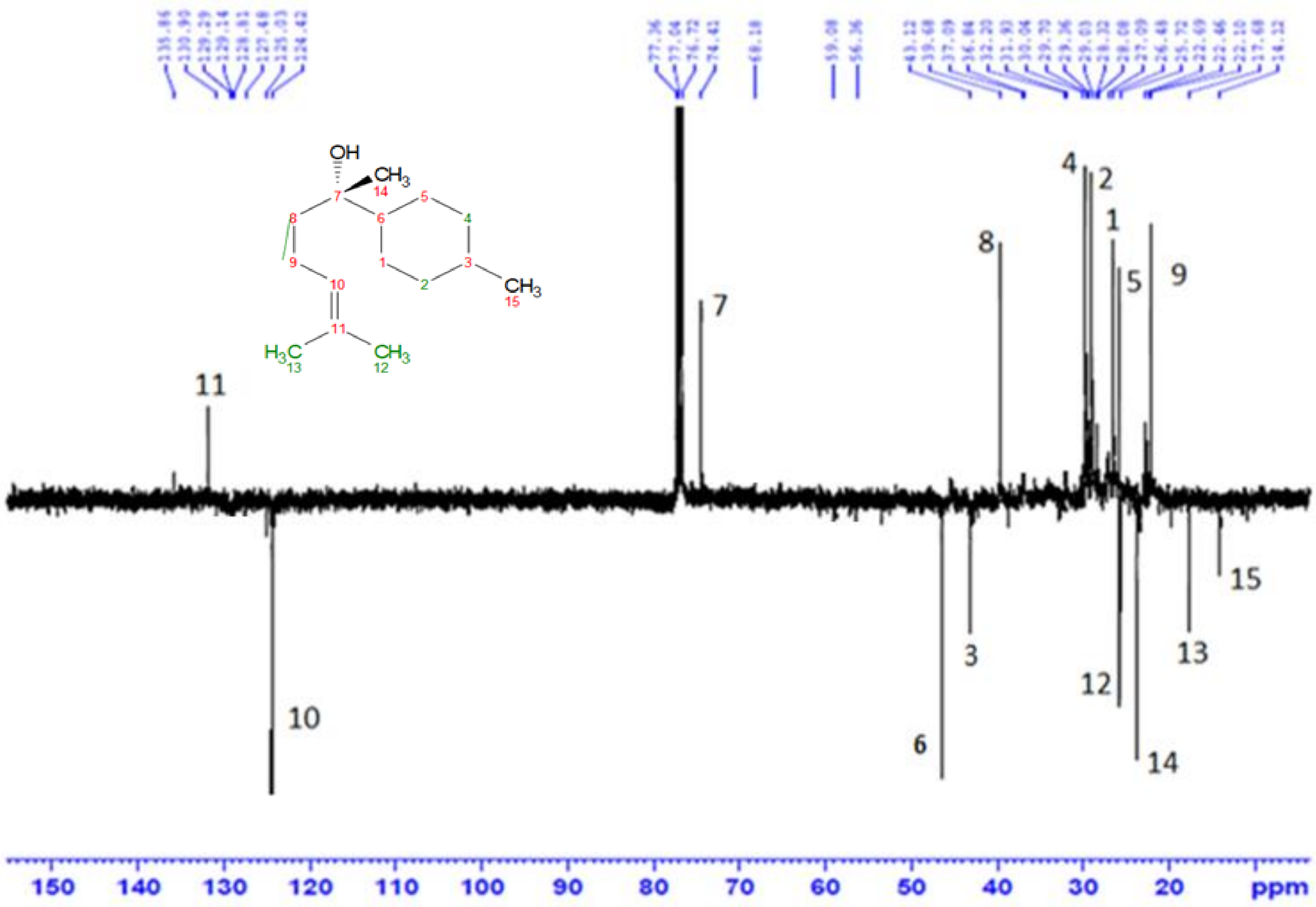

Figure S28. APT spectrum of compound 4 ($CDCl_3$, 100 MHz).

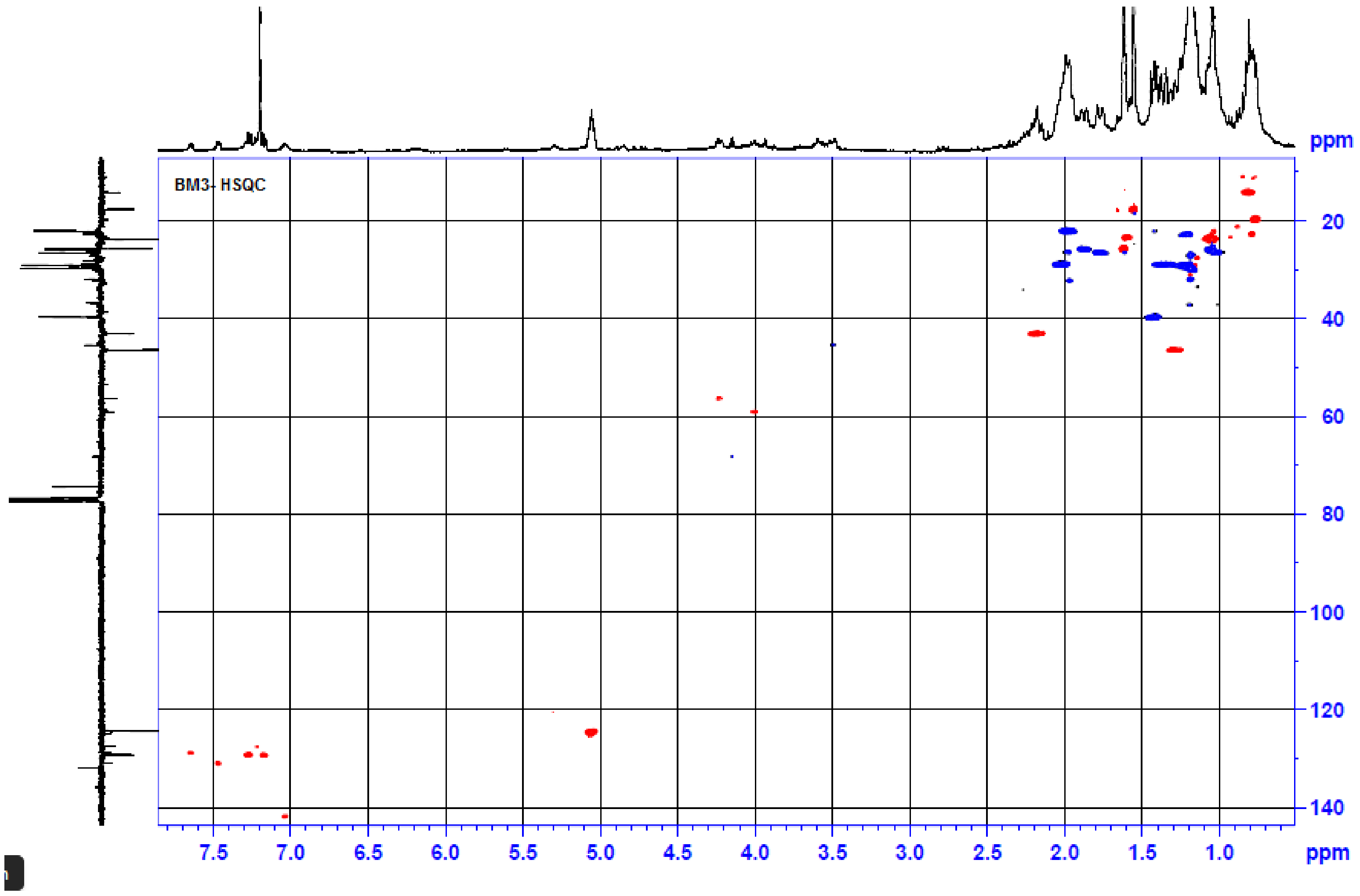

Figure S29. HSQC spectrum of compound 4.

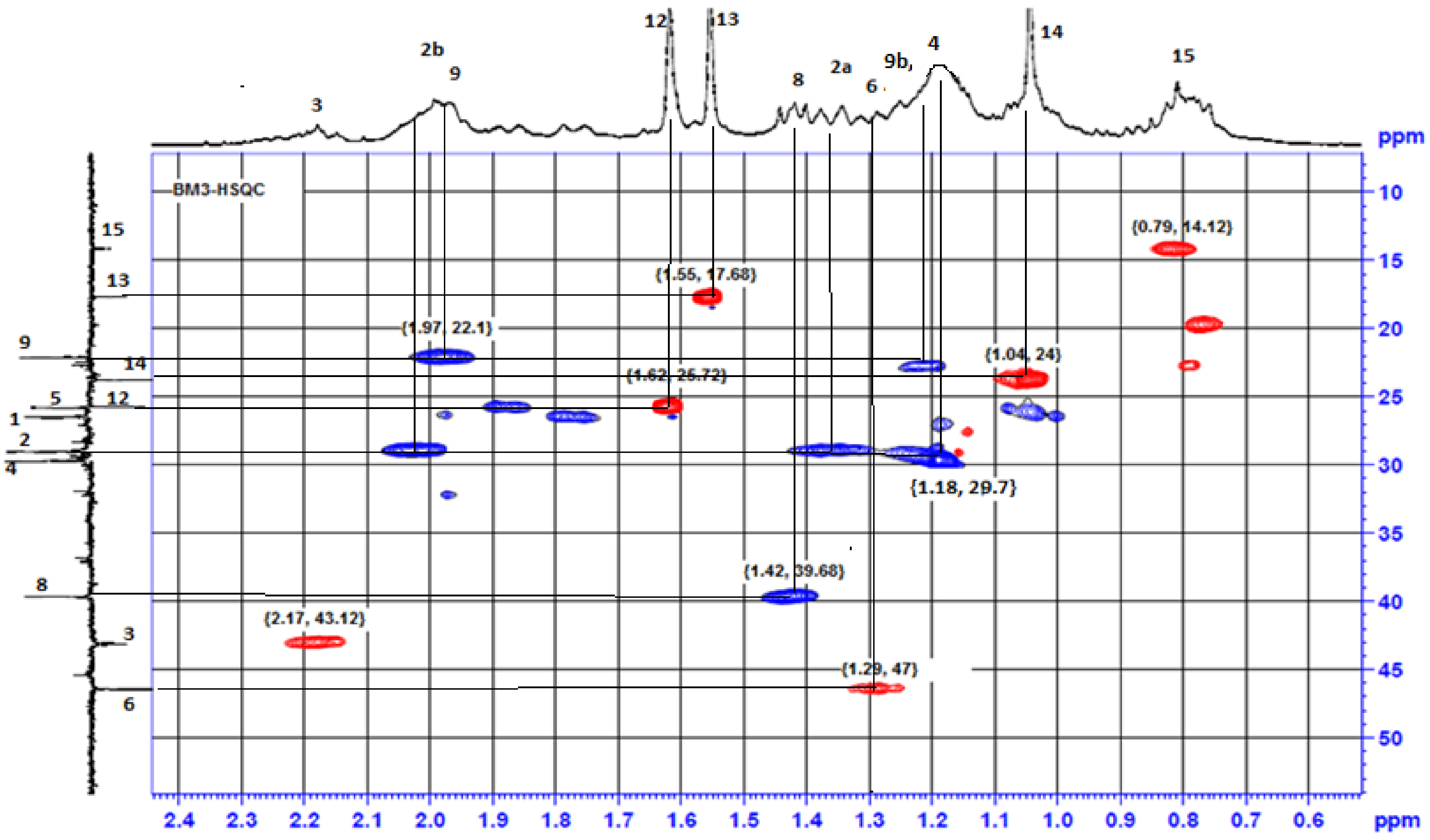

Figure S30. HSQC expansion of compound 4.

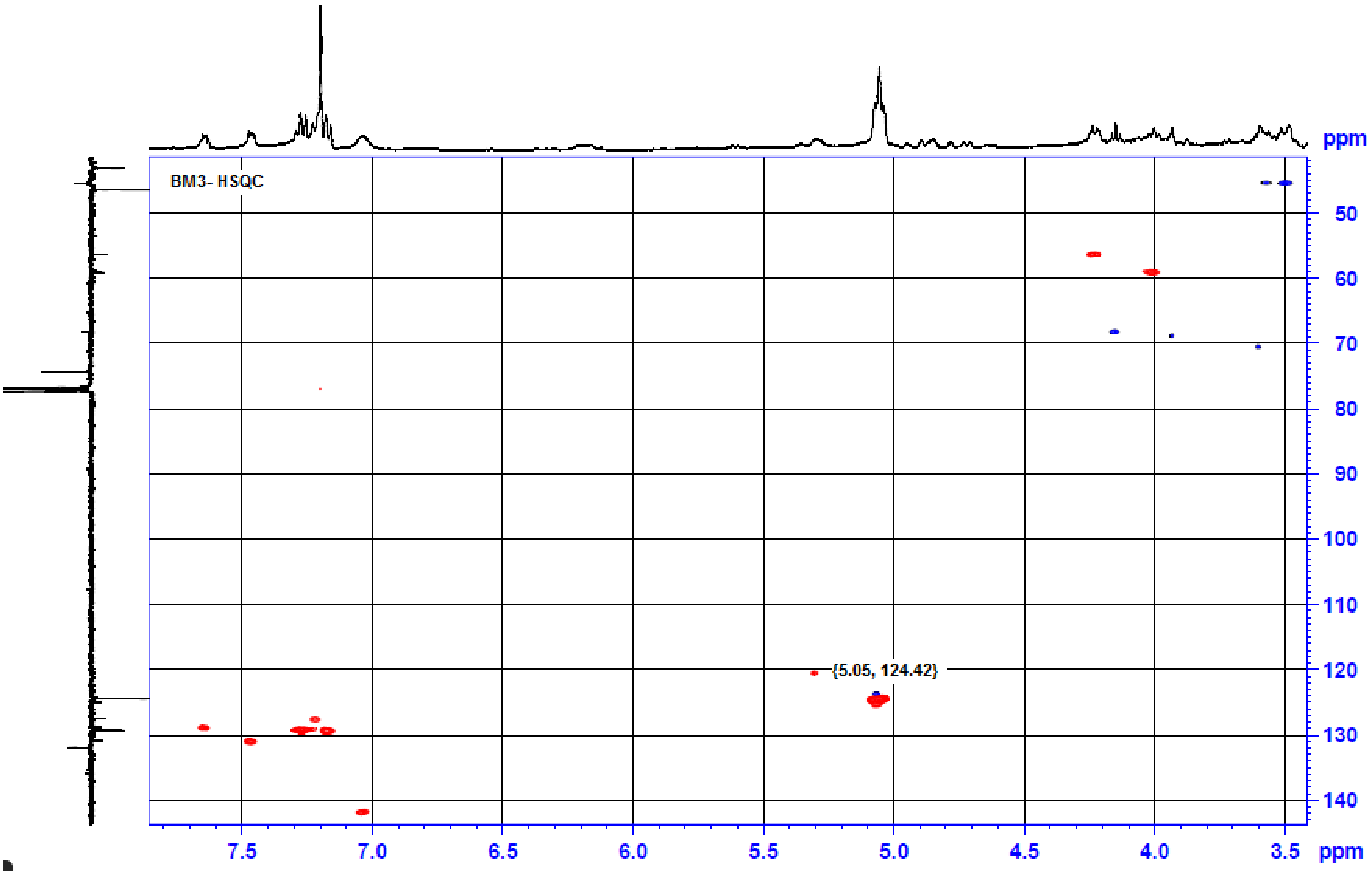

Figure S31. HSQC spectrum expansion of compound 4.

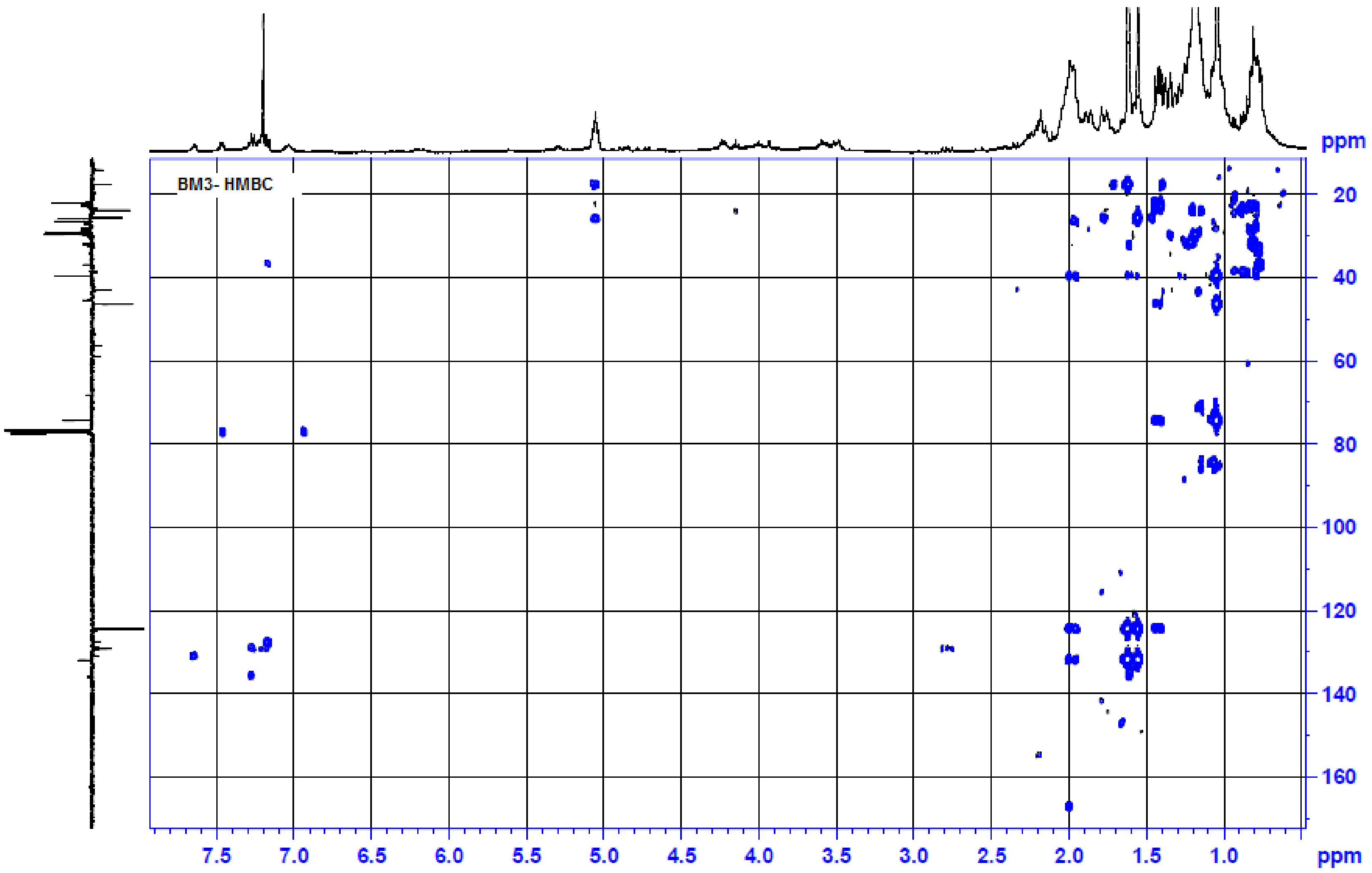

Figure S32. HMBC spectrum of compound 4.

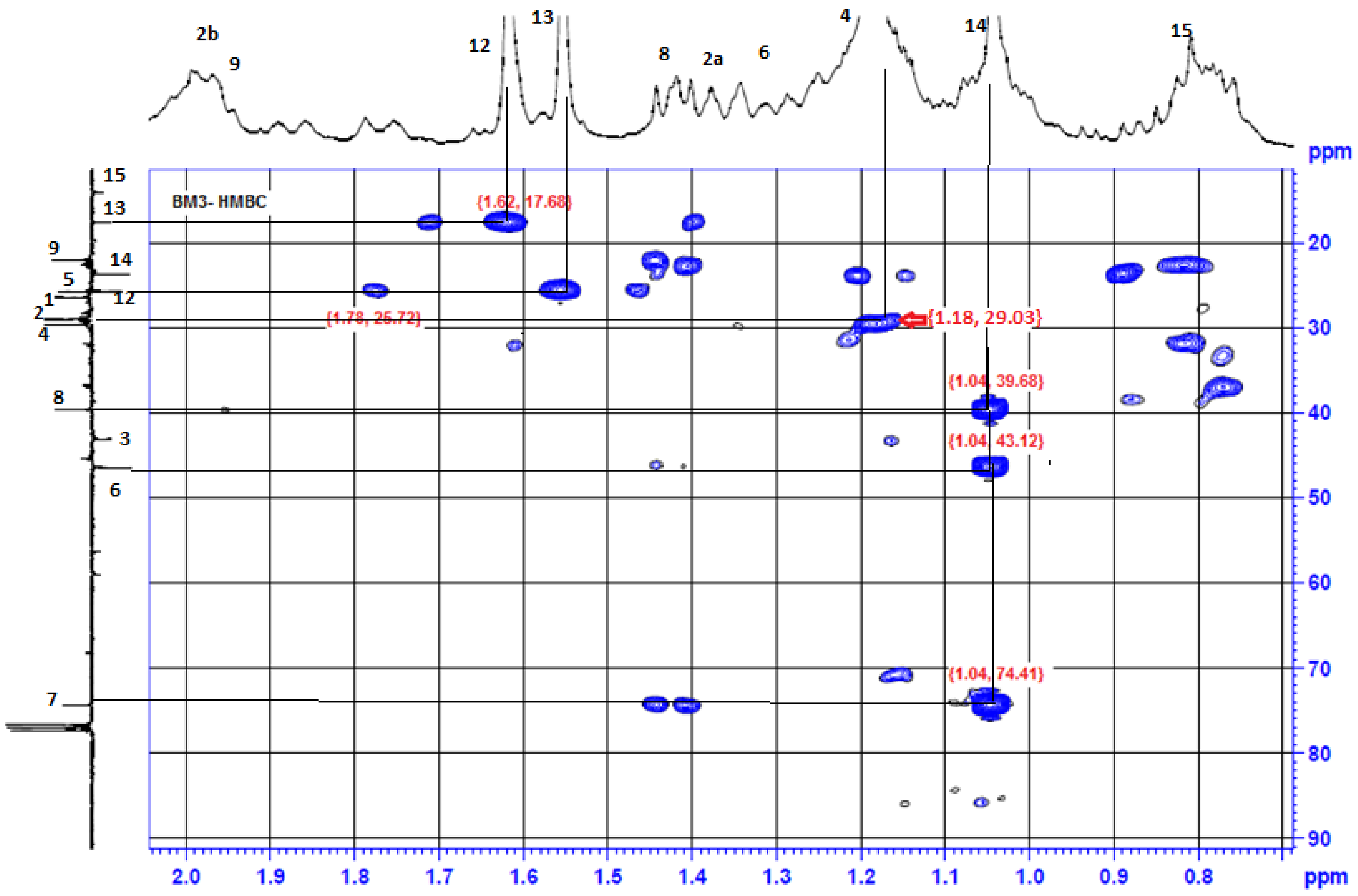

Figure S33. HMBC spectrum expansion (X =0.8 -2) of compound 4.

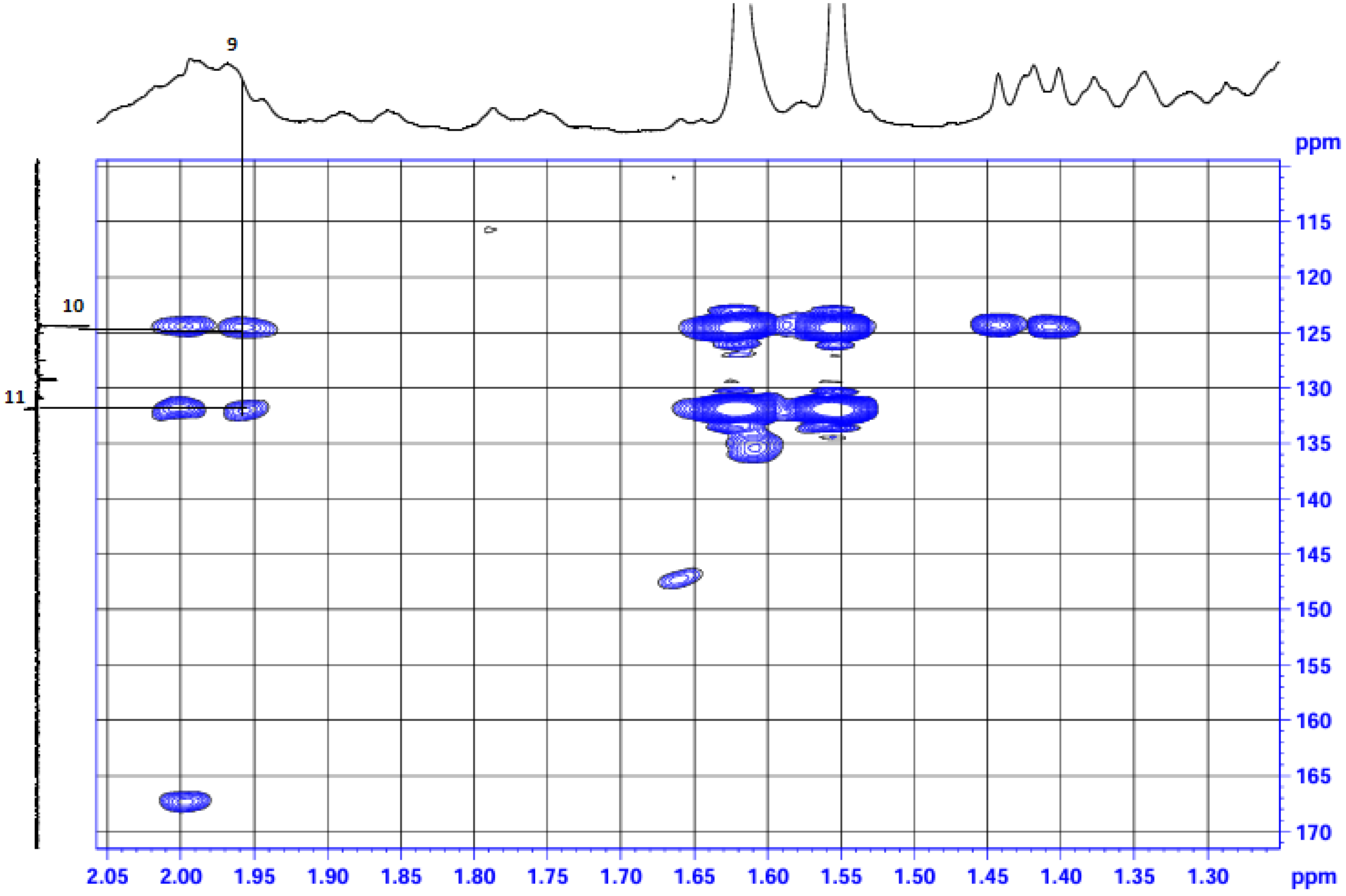

Figure S34. HMBC spectrum expansion (X= 1.03- 2.05 ppm) of compound 4.

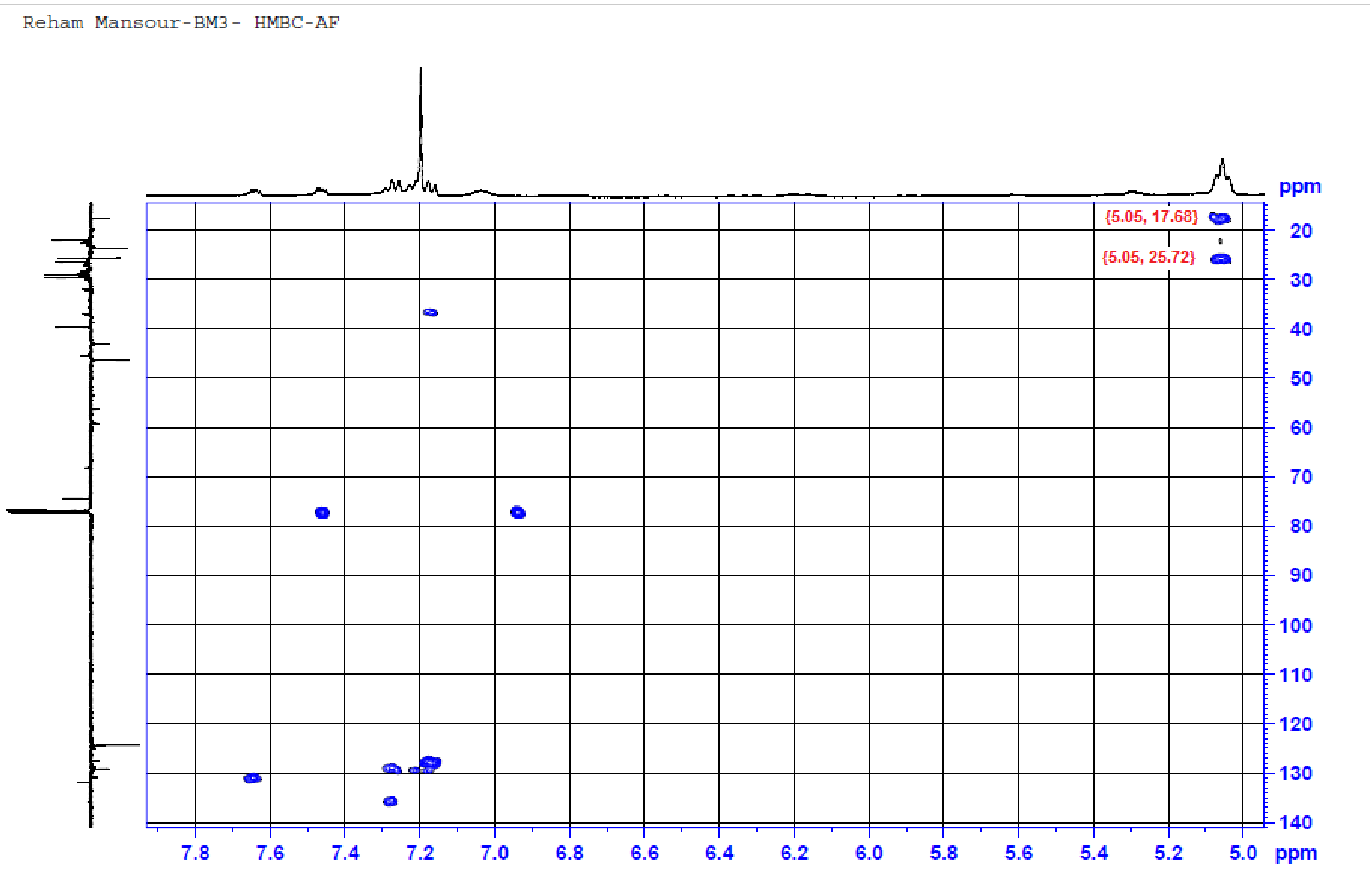

Figure S35. HMBC spectrum expansion (X= 5- 7.8 ppm) of compound 4.

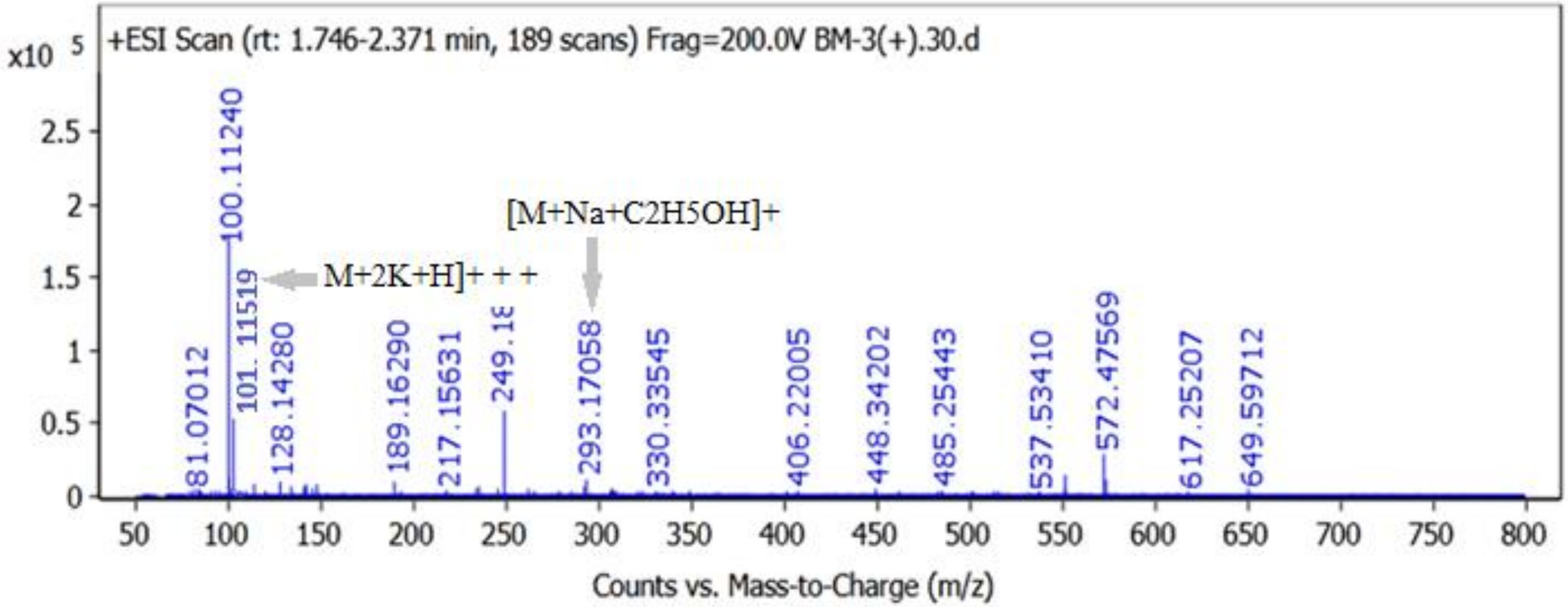

Figure S36. HR-ESI-MS spectrum of compound 4.

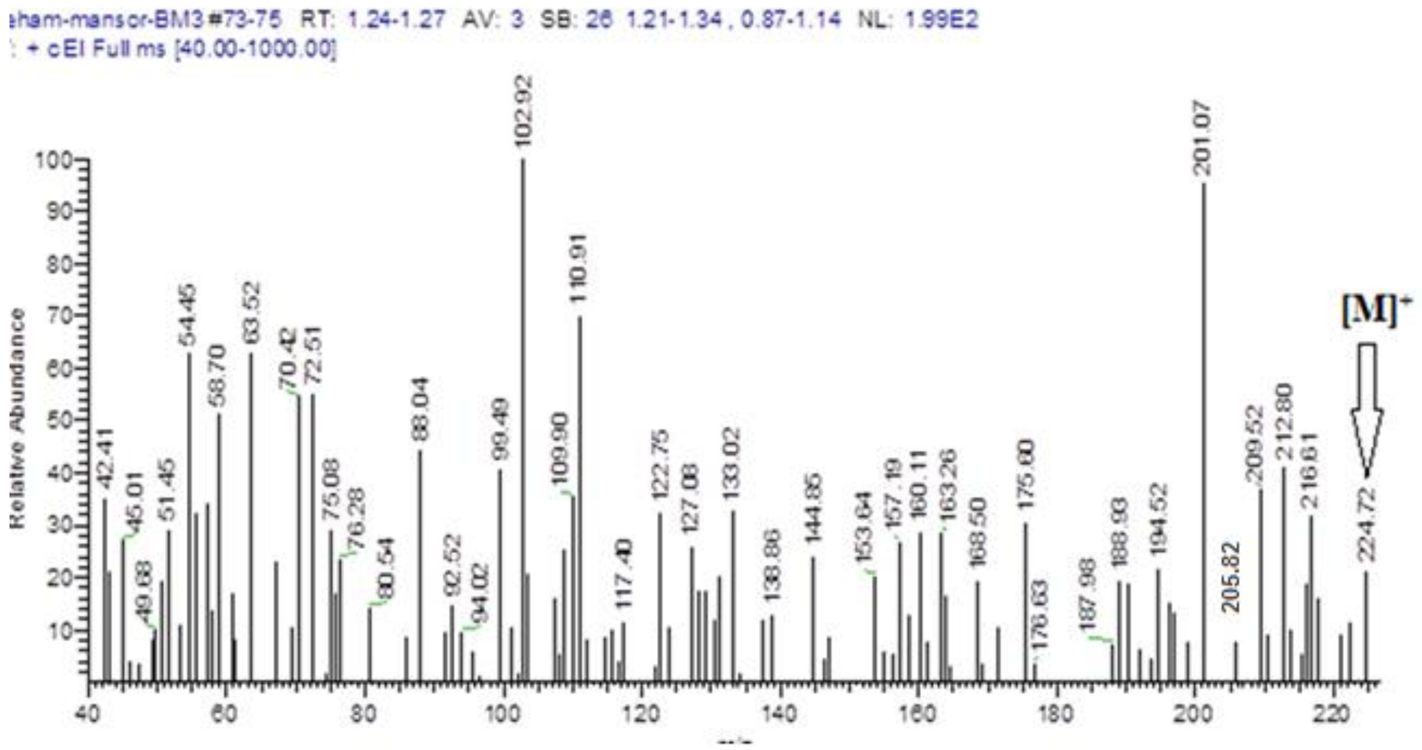

Figure S37. EI-MS spectrum of compound 4.

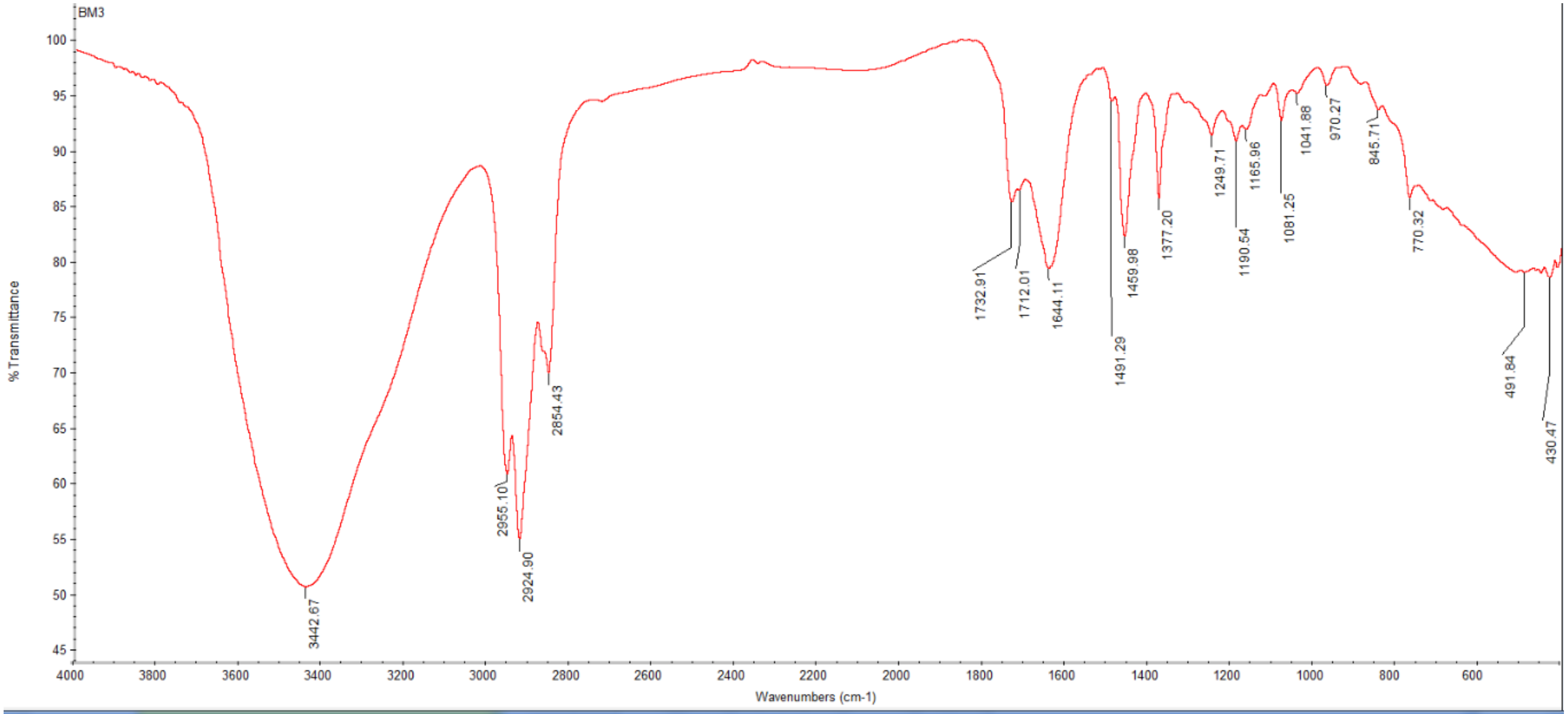

Figure S38. IR spectrum of compound 4.

Spectral data of compound 5

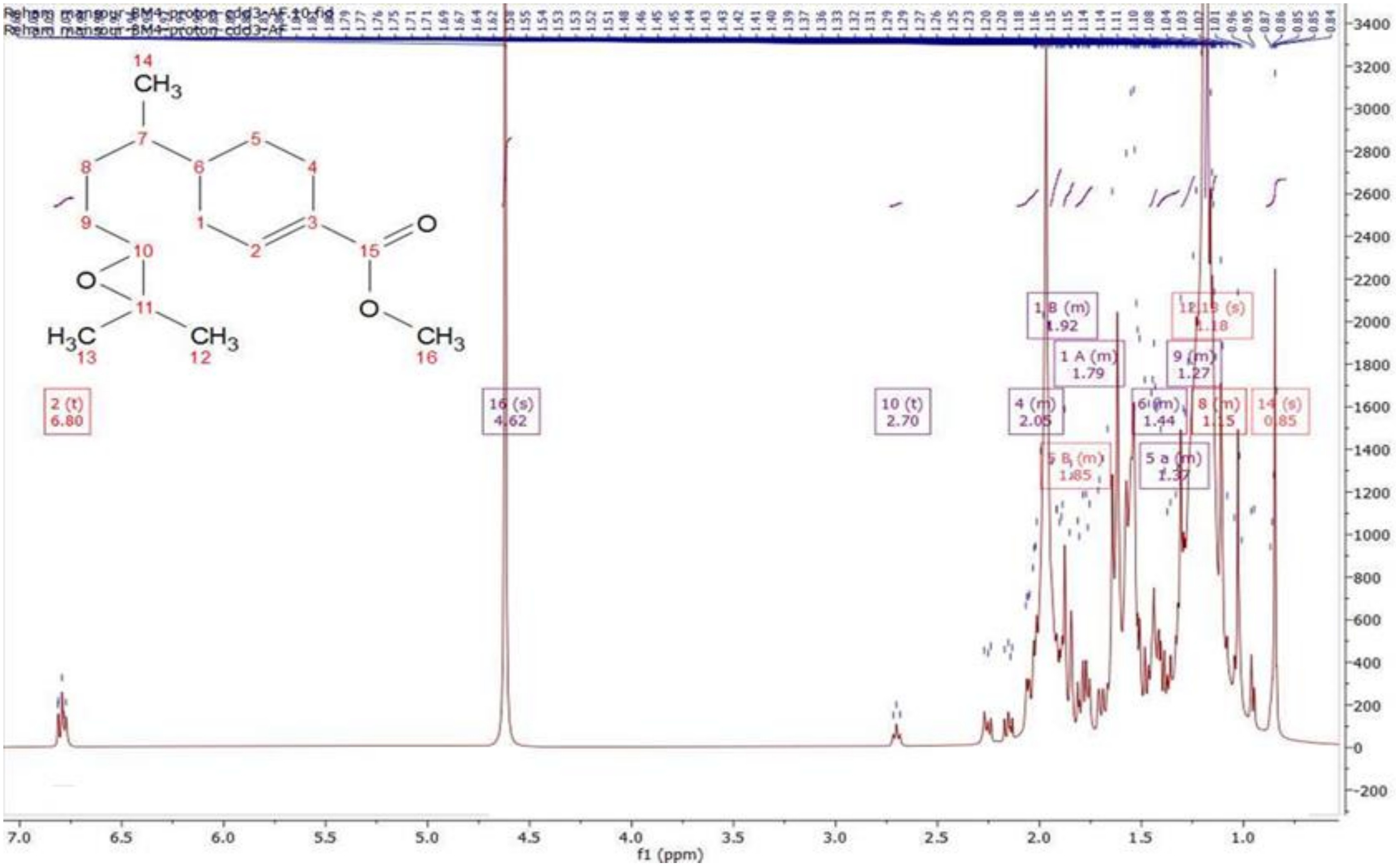

Figure S39. ^{1}H NMR spectrum of compound 5 ($CDCl_3$, 400 MHz).

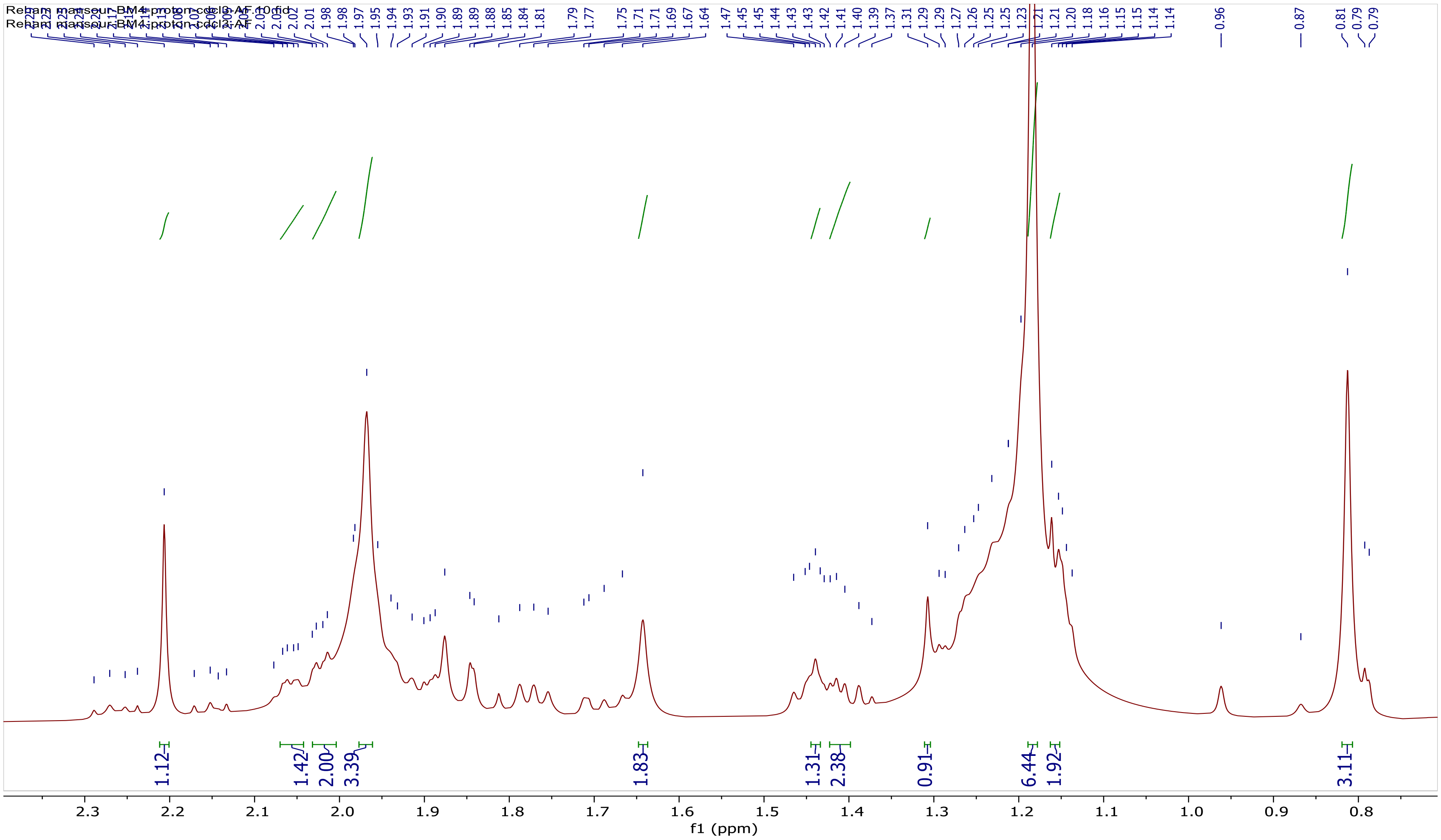

Figure S40. ^{1}H NMR spectrum expansion (0.8- 2.3ppm) of compound 5 ($CDCl_3$, 400 MHz).

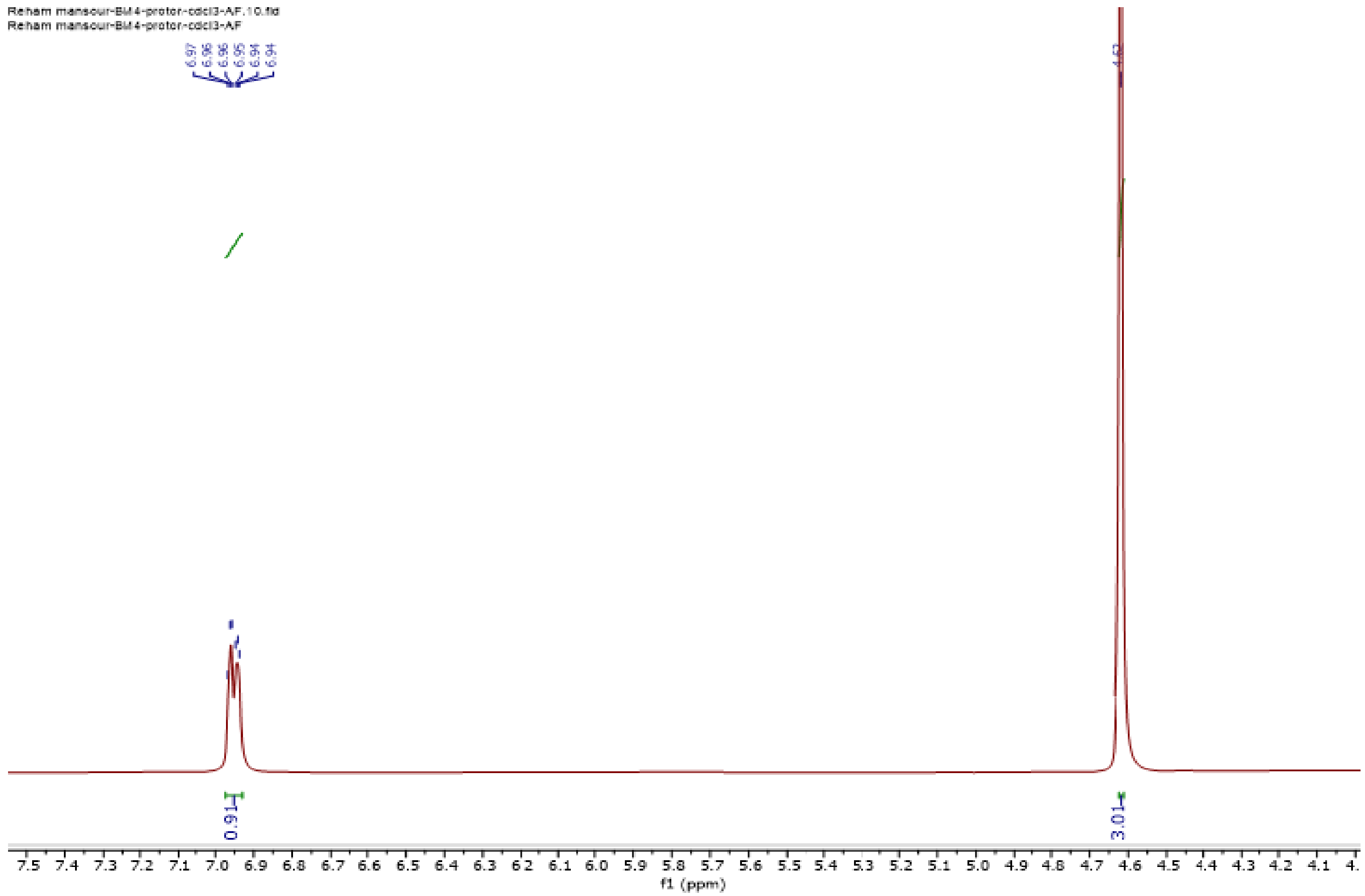

Figure S41. ^{1}H NMR spectrum expansion (4.0- 7.5 ppm) of compound 5 ($CDCl_3$, 400 MHz).

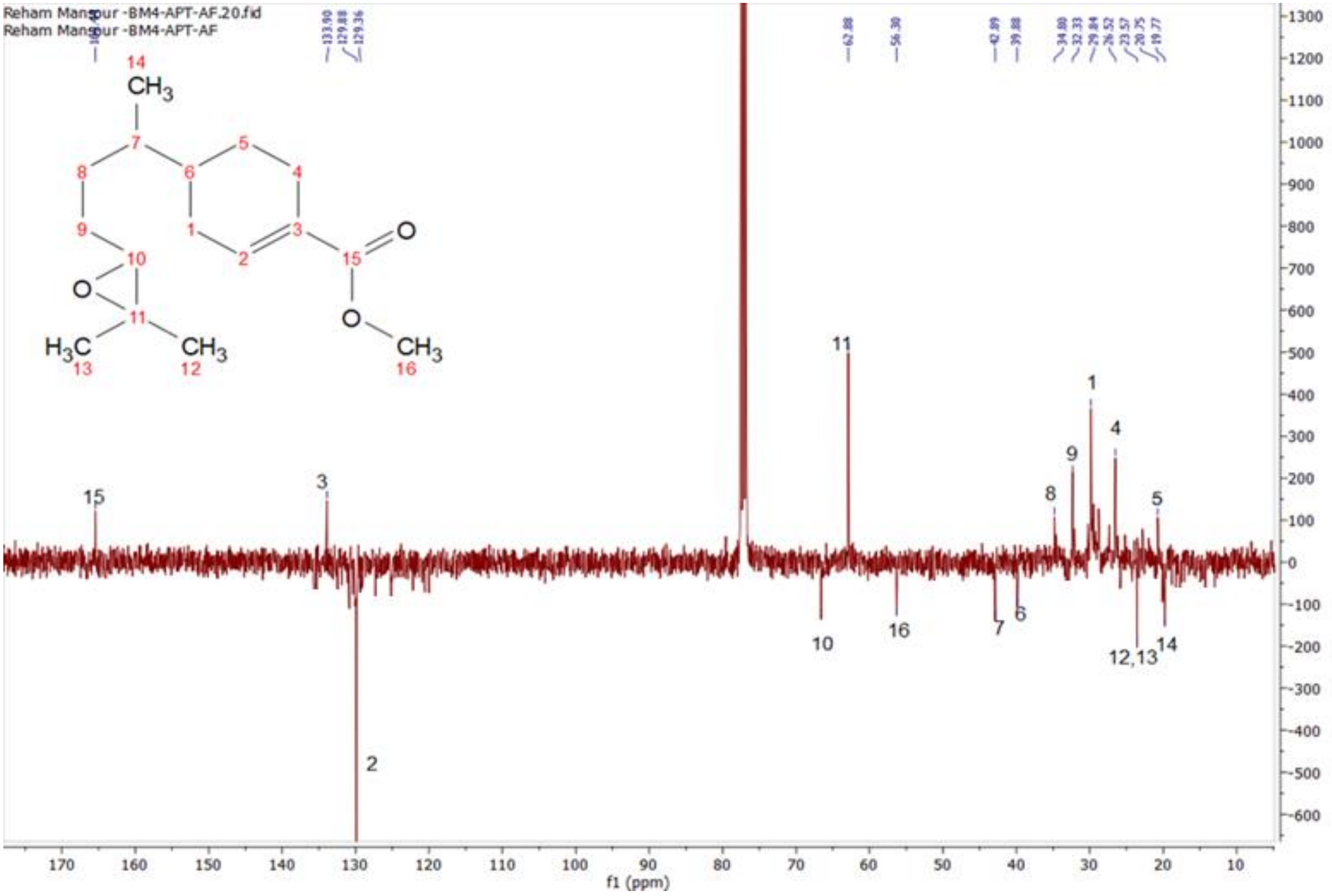

Figure S42. APT spectrum of compound 5 ($CDCl_3$, 100 MHz).

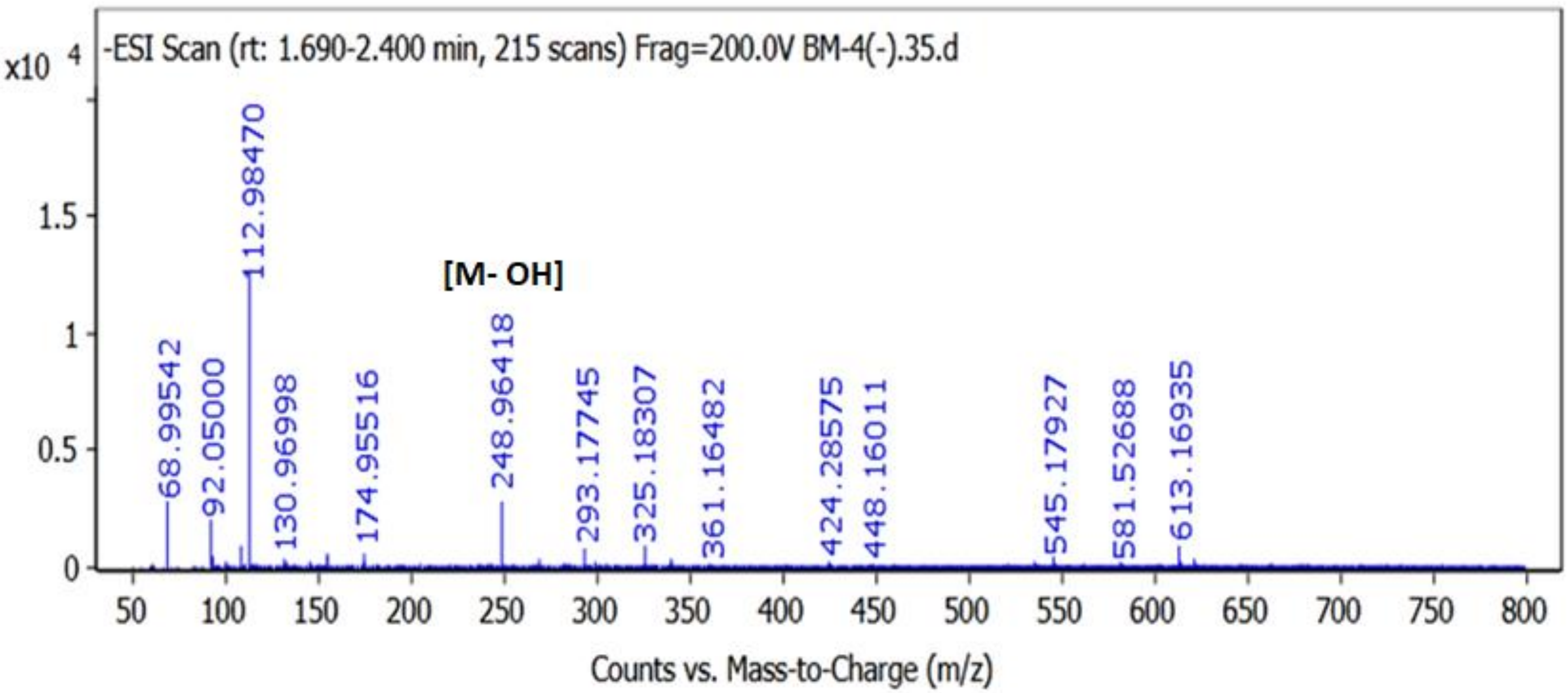

Figure S43. HR-ESI-Mass spectrum of compound 5.

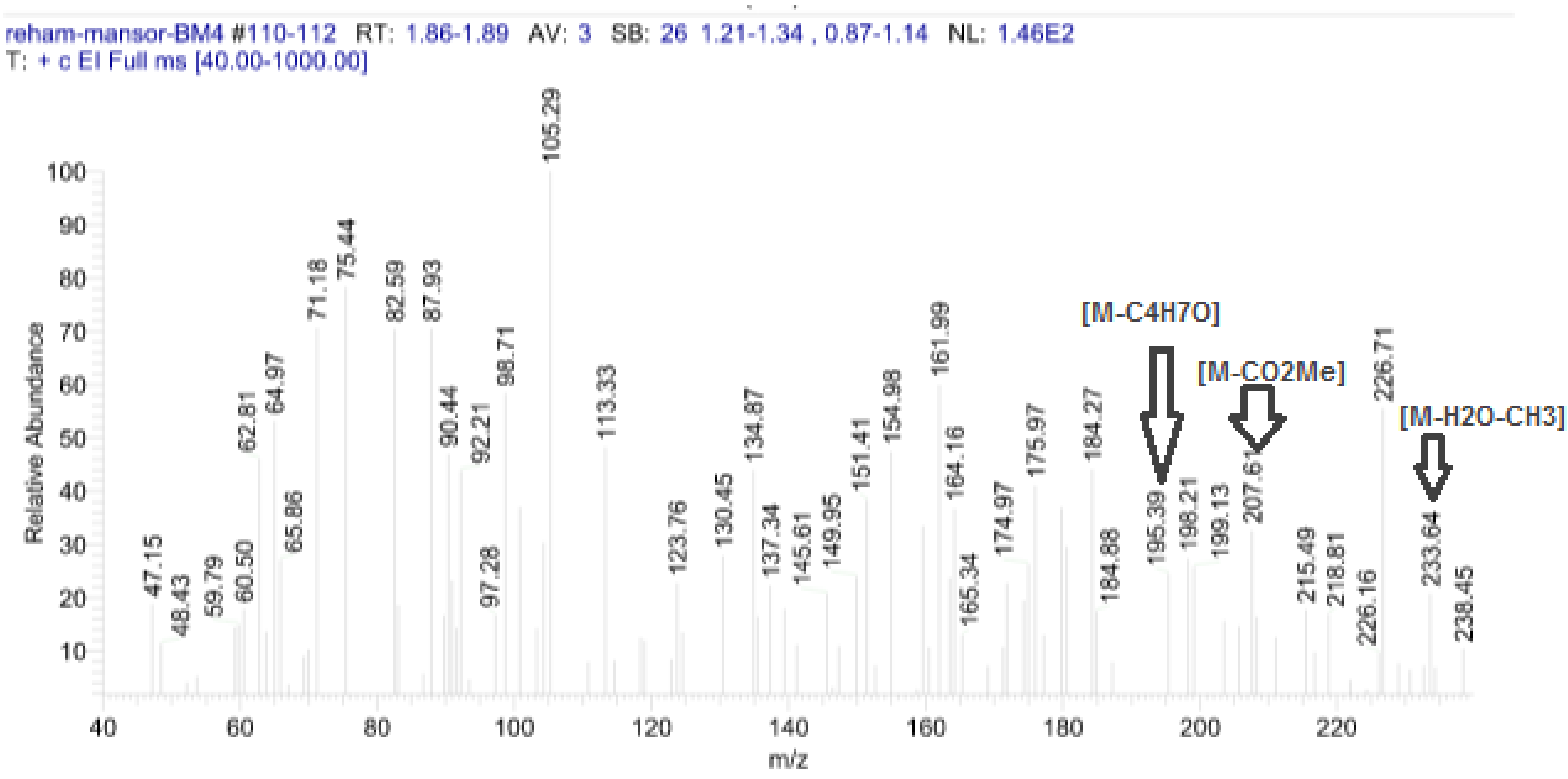

Figure S44. EI-Mass spectrum of compound 5.

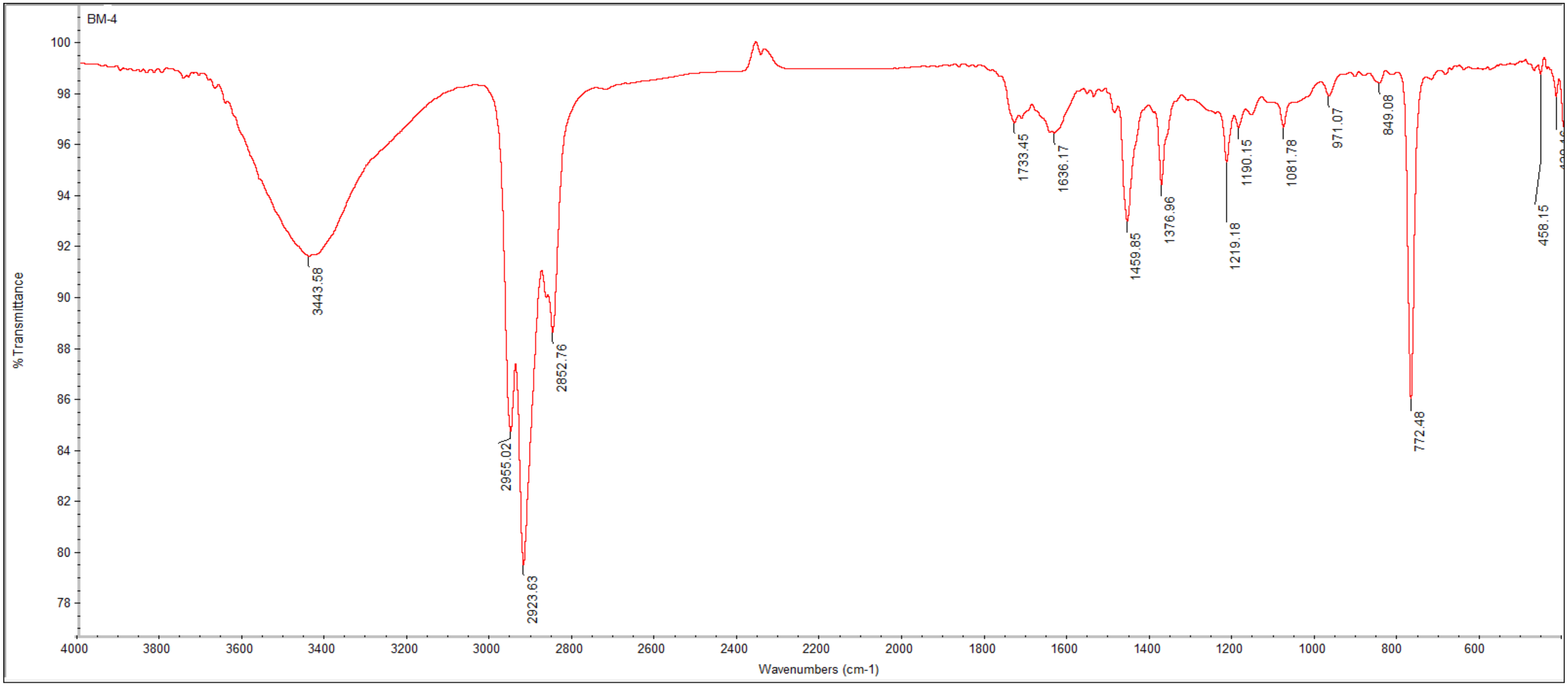

Figure S45. IR spectrum of compound 5.

Spectral data of compound 6

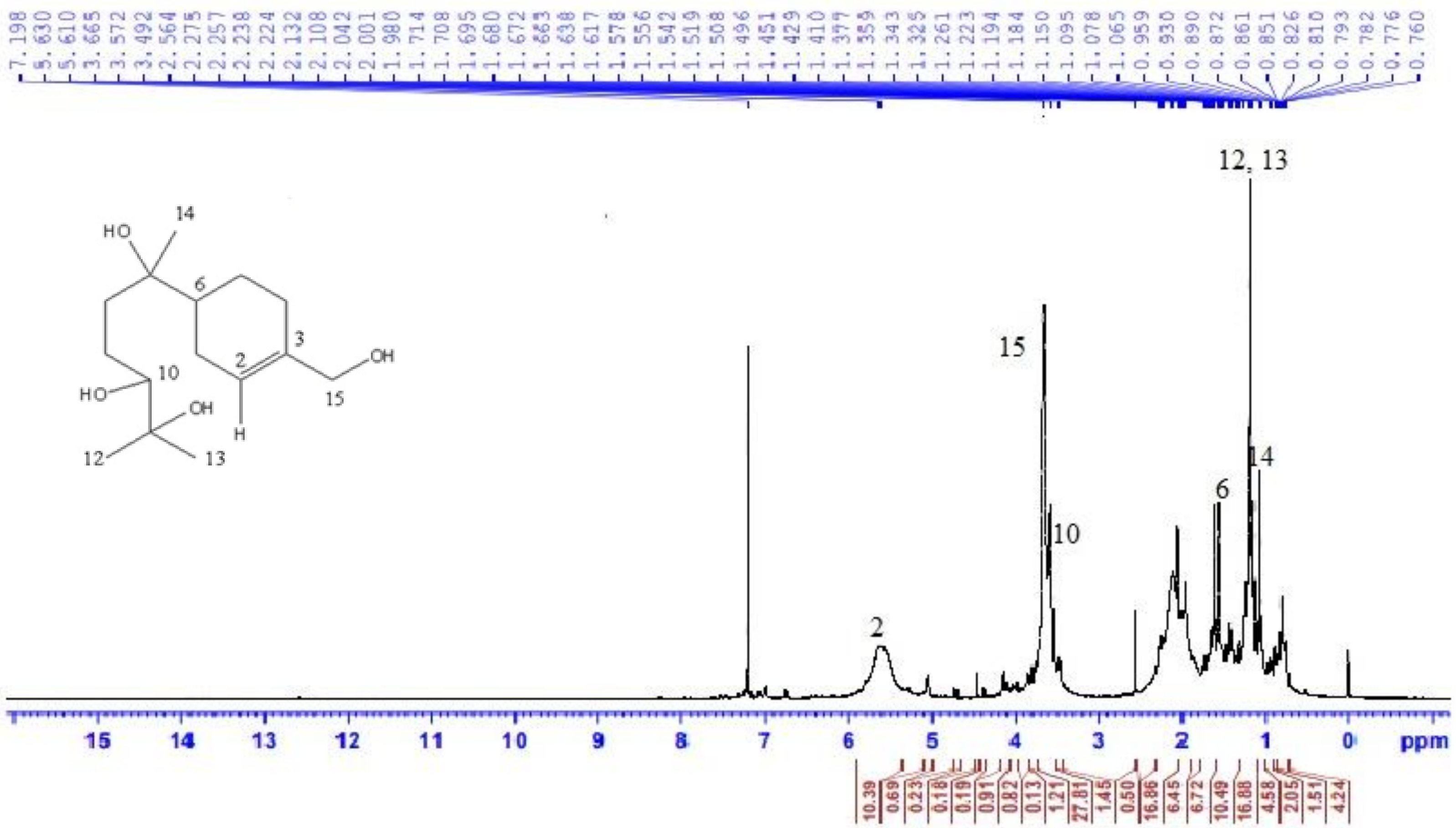

Figure S46. ^{1}H NMR spectrum of compound 6 ($CDCl_3$, 400 MHz).

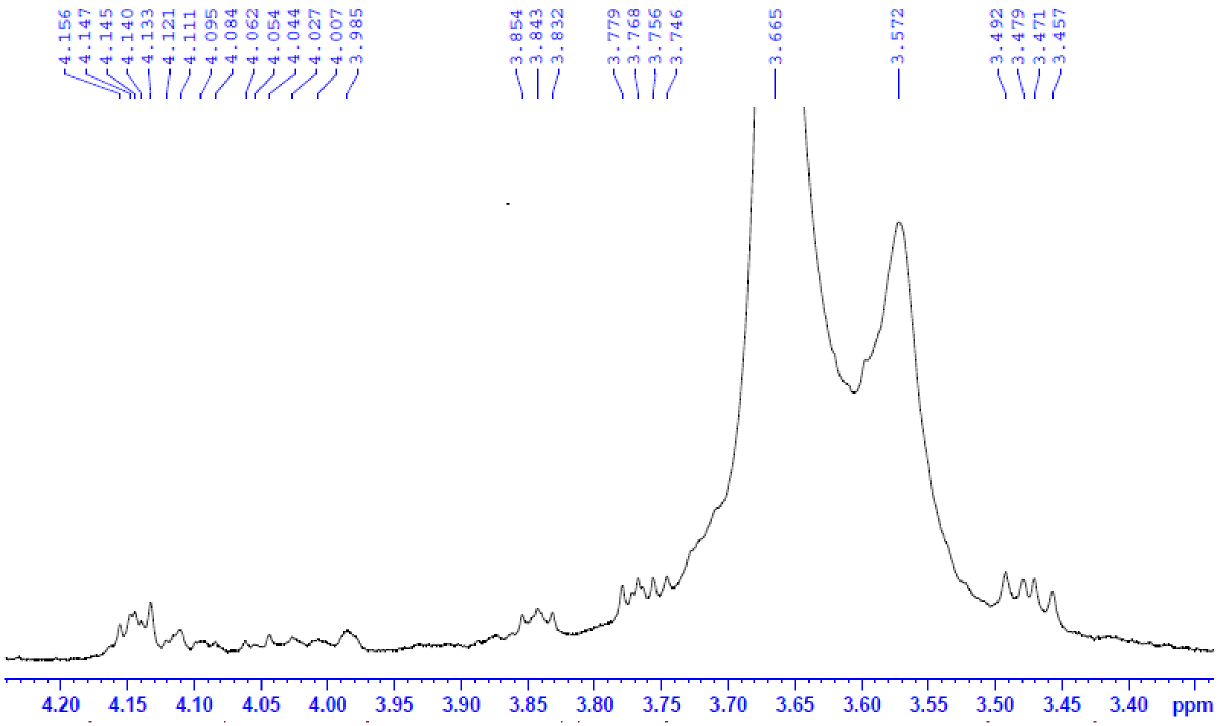

Figure S47. ^{1}H NMR spectrum expansion (3.4- 4.2 ppm) of compound 6 ($CDCl_3$, 400 MHz).

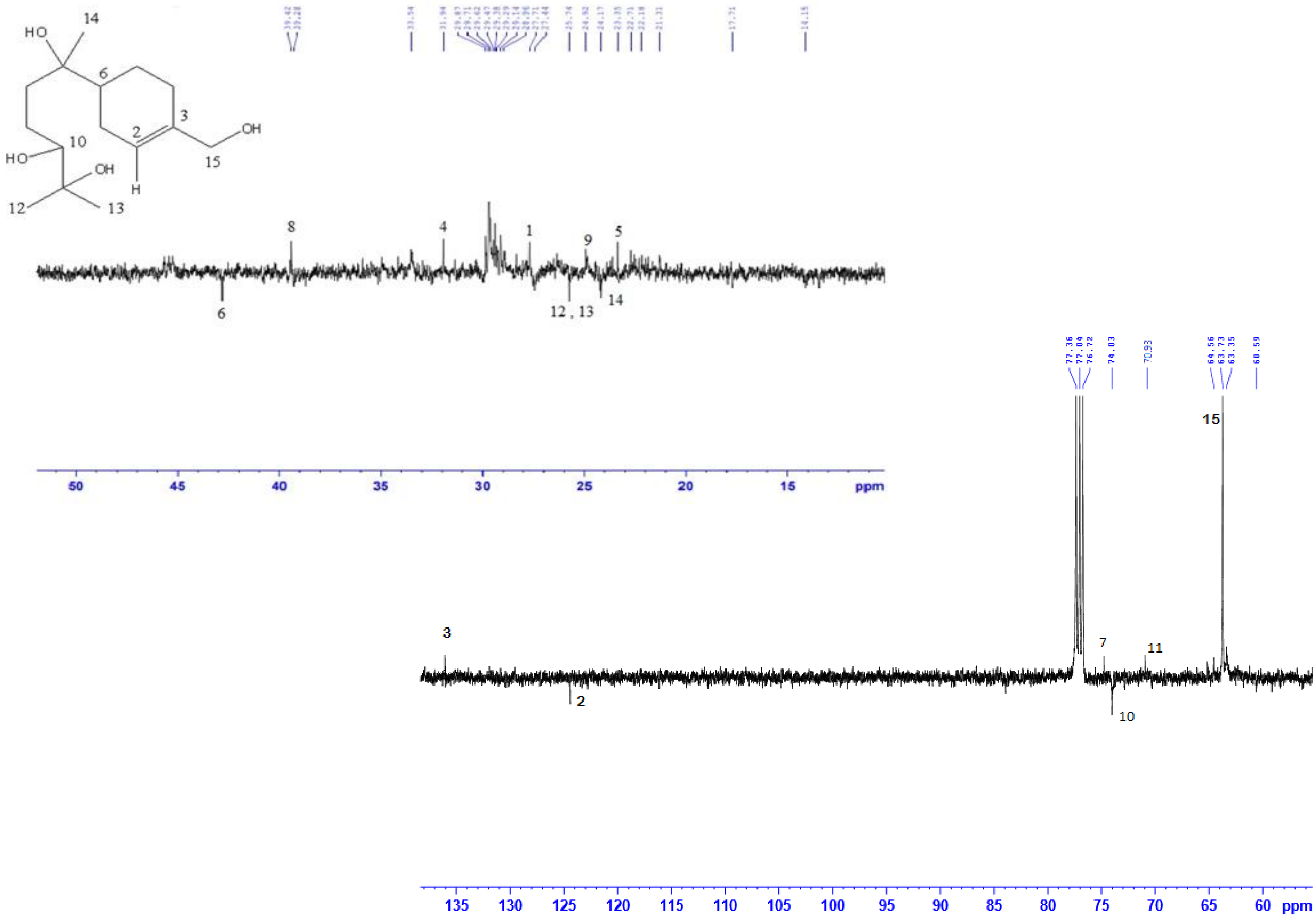

Figure S48. APT spectrum of compound 6 ($CDCl_3$, 100 MHz).

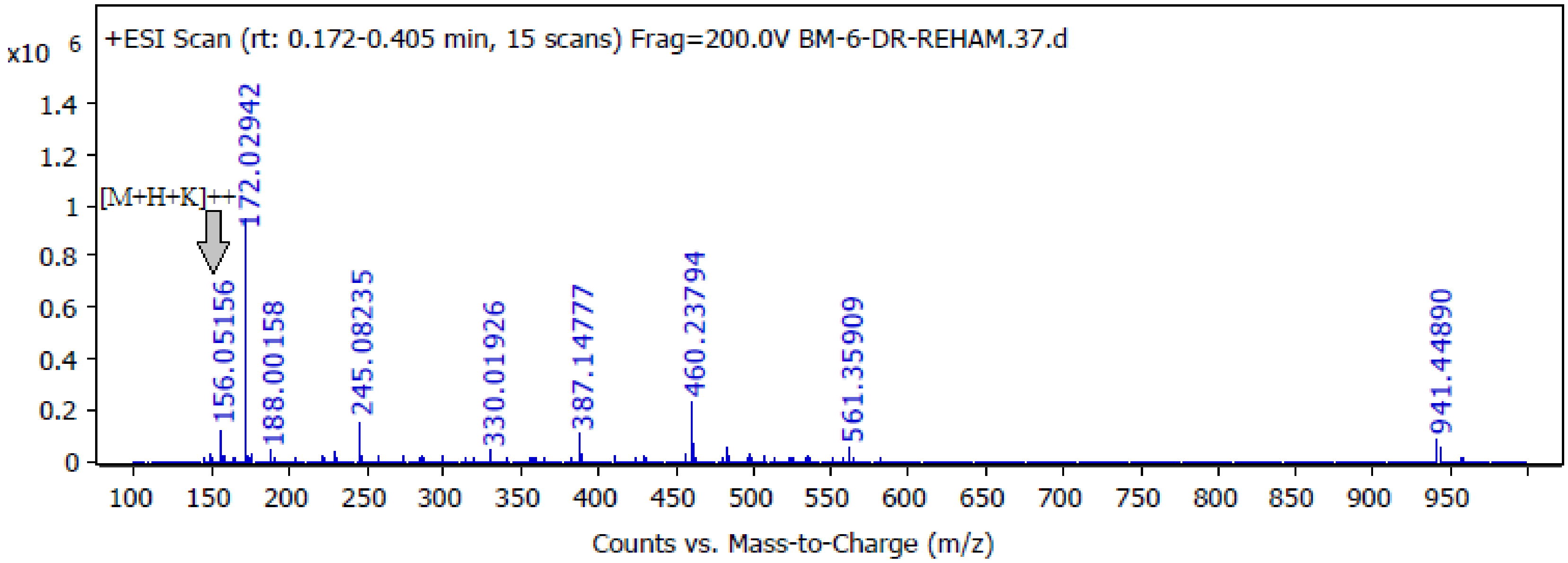

Figure S49. HR-ESI-MS spectrum of compound 6.

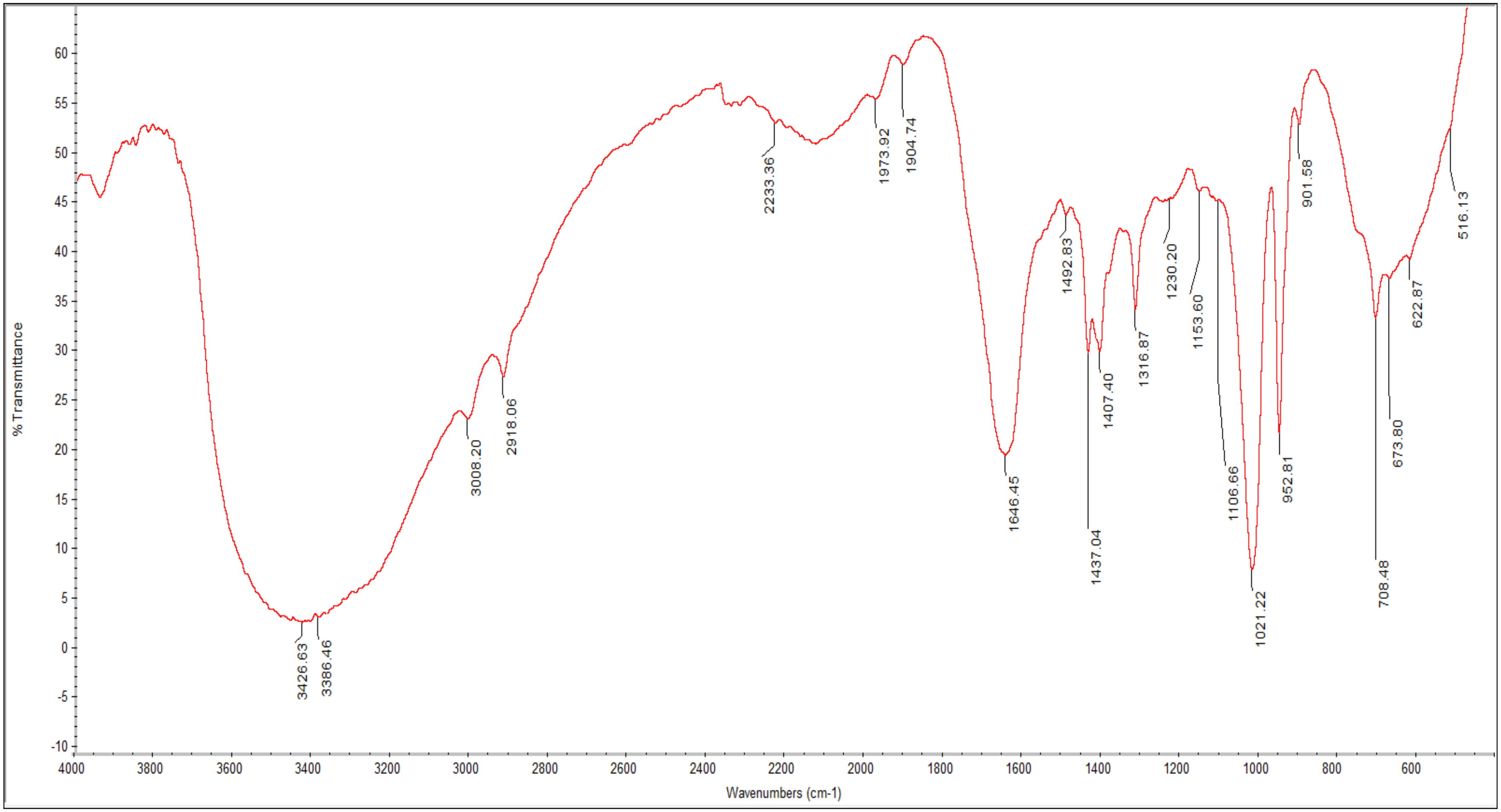

Figure S50. IR spectrum of compound 6.

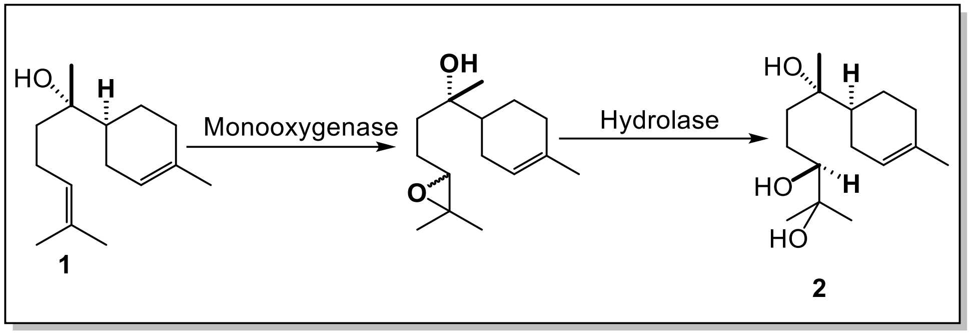

Figure S51. The proposed biosynthetic pathway of metabolite 2 resulted from biotrnsformation of 1 with *Cordyceps sinensis* ATCC 24400. It suggests that the transformation involves enzymatic epoxidation of the C-10(11) double bond by a monooxygenase enzyme, followed by hydrolytic cleavage of the resulting epoxide by a hydrolase enzyme

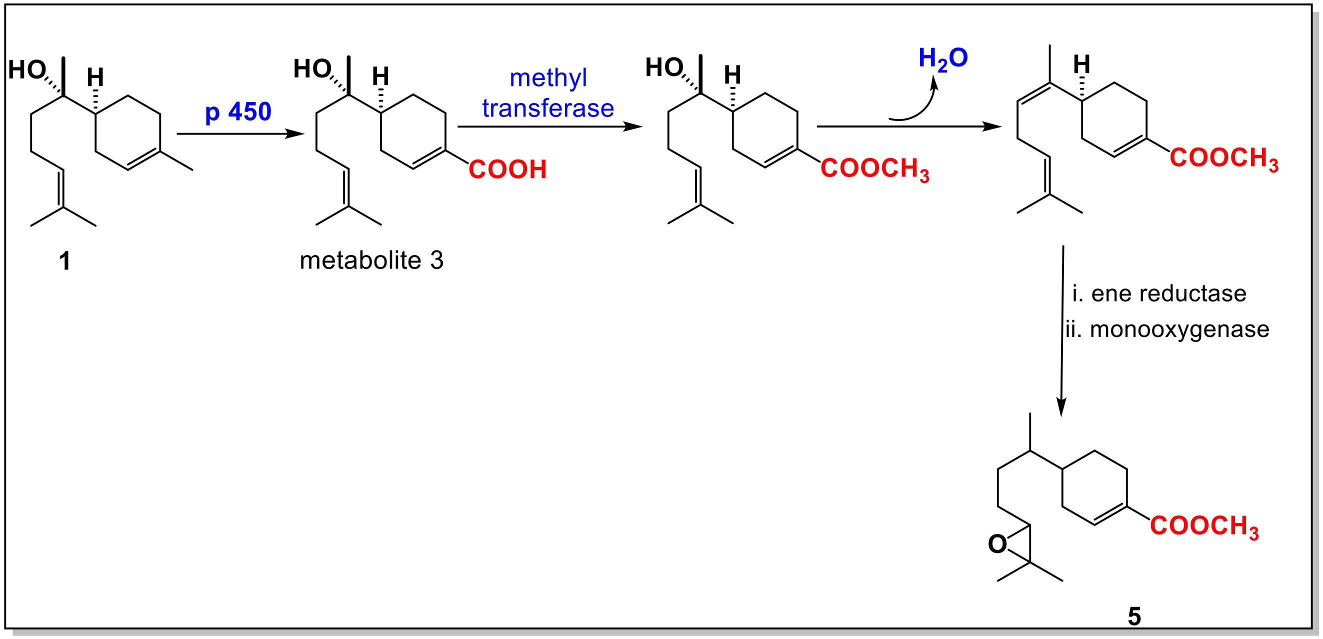

Figure S52. The proposed biosynthetic pathway of **5** resulted from biotrnsformation of **1** with *Aspergillus flavus* ATCC 16883 could be explained as follows: first, the methyl group attached to C-3 is oxidized to a carboxylic acid group, forming metabolite 3, which is then esterified to form 3-methylcarboxy-α-bisabolol. This esterification step likely involves an S-adenosylmethionine-dependent methyl-transferase, a member of a large family of enzymes with broad biological activity, including trans-methylation. Second, dehydration occurs leading to the formation of a double bond between C7 and C8, which removes the hydroxyl group from the lateral aliphatic chain of 3-methylcarboxy-α-bisabolol. Third, reduction of the newly formed C-7(8) double bond by ene reductase enzyme resulting in formation of 7-dehydroxy-3-methylcarboxy-α-bisabolol. Fourth, epoxidation reaction between C-10 and C-11 resulting in the formation of 7-dehyroxy-10,11-epoxy-3-methylcarboxy-α-bisabolol.

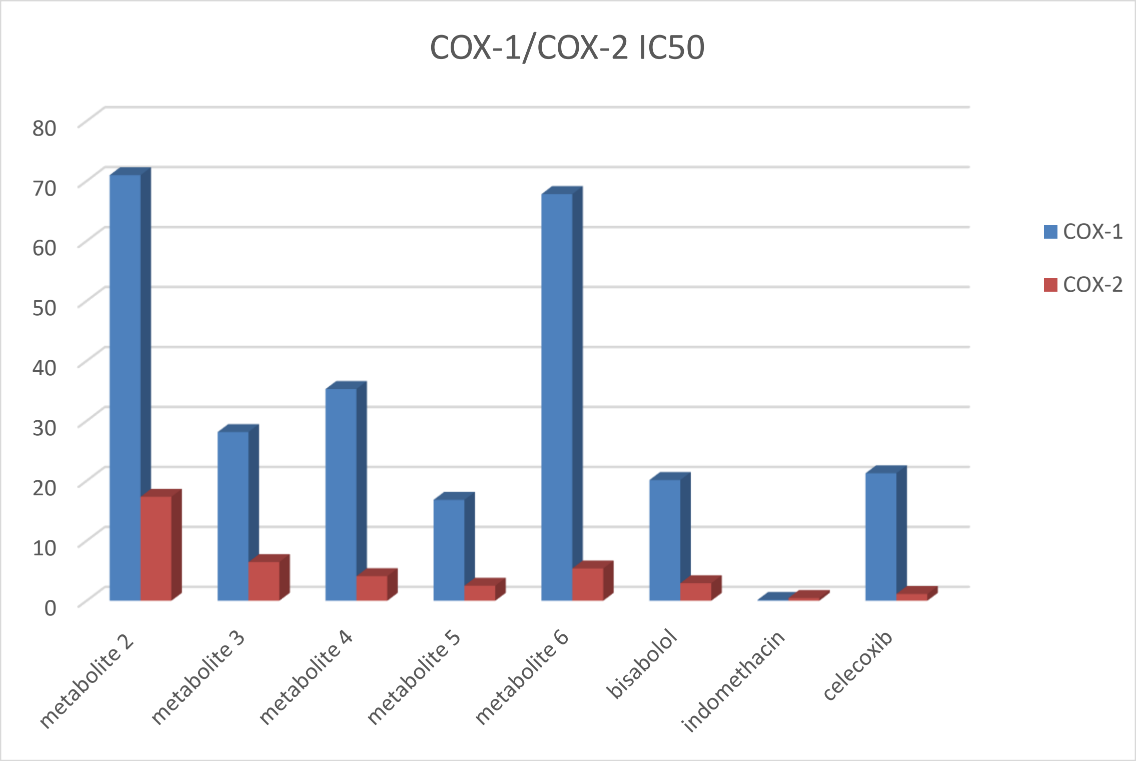

Figure S53. COX-1 and COX-2 minimum inhibitory dose causing 50% activity (IC_{50})(µM) of bisabolol and its five metabolites and reference compounds(indomethacin and celecoxib), sample size =10µl. Results are presented as means ± standard deviations (SD), with n = 3. Indomethacin is a non-selective COX inhibitor, while celecoxib is a selective COX-2 inhibitor. Metabolite **5** was slightly more potent as COX-2 inhibitor than α-bisabolol.

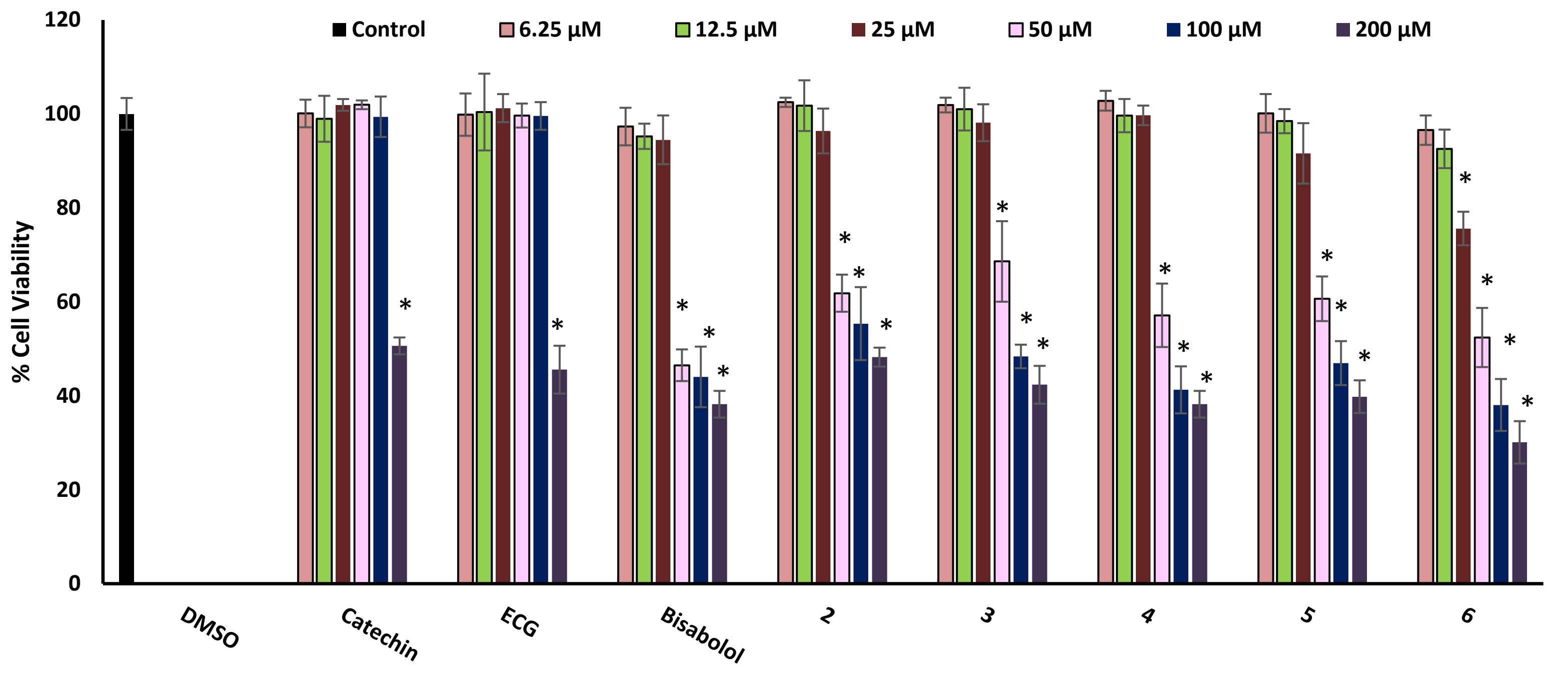

Figure S54. The effect of α-bisabolol, its metabolites and positive controls: catechin and epicatechin-3-gallate (ECG) on the SH-SY5Y cells viability. Results are presented as means ± standard deviations (SD), with n = 5. Cell viability was expressed as a percentage of WST-1 reduction, considering that the viability of cells treated with 1% DMSO as a negative control was 100%. * Indicates a statistically significant difference compared to the cell viability of DMSO-treated cells at $p < 0.001$. Statistical significance was determined using one-way ANOVA followed by Dunnett's post-hoc test in GraphPad Prism® 10. Bisabolol and its metabolites (except for metabolite 6), were non-cytotoxic at a conctrations ≤ 25 µM. Metabolite **6** showed no significant cytotoxicity at concentration ≤ 12.5 µM.

Table S1. ^{1}H NMR (400 MHz) spectroscopic data for α-bisabolol and compounds 2,3,4,5 and 6 (in $CDCl_3$).

C	α -bisabolol	δH (multiplicities, *J* in Hz)				
		2	3	4	5	6
**1**	1.85-1.73 (m, 1H, Hα) 2.00-1.95 (m, 1H, Hβ)	1.85-1.73 (m, 1H, Hα) 2.00-1.95 (m, 1H, Hβ)	2.13-2.03 (m, 1H, Hα) 2.46 – 2.42 (d, 1H, Hβ)	1.78 – 1.75 (d, 2H)	1.82-1.74 (m, 1H, Hα) 1.95-1.89 (m, 1H, Hβ)	2.00-1.95 (m, 2H)
**2**	5.32 (m, 1H)	5.28 (m, 1H)	7.01(1H)	1.35(1H, Hα) 1.99 (1H, Hβ)	6.94 (dd, 1H)	5.65 (br.s, 1H)
**3**	-	-	-	2.17 (m,1H)	-	-
**4**	1.94-1.93 (m, 2H)	1.94-1.93 (m, 2H)	2.24-2.19(m, 2H)	1.24- 1.18 (m, 2H)	2.11-2.00 (m, 2H)	1.99-1.89 (m, 2H)
**5**	1.29-1.16 (m, 1H, Hα) 1.90-1.84 (m, 1H, Hβ)	1.29-1.16 (m, 1H, Hα) 1.90-1.84 (m, 1H, Hβ)	1.24-1.18 (m, 2H)	1.89 – 1.86 (m,2H)	1.42-1.32 (m, 1H, Hα) 1.88-1.82 (m, 1H, Hβ)	1.61-1.35 (m, 2H)
**6**	1.55-1.48 (m, 1H)	1.55-1.48 (m, 1H)	1.55-1.48 (m, 1H)	1.29 (m,1H)	1.46-1.42 (m, 1H)	1.55 (m, 2H)
**7**	-	-	-	-	1.44 (1H)	-
**8**	1.47-1.42 (m, 2H)	1.47-1.42 (m, 2H)	1.47-1.42 (m, 2H)	1.44-1.42 (m, 2H)	1.16 (m, 2H)	1.33 (m, 2H)
**9**	2.04-1.97 (m, 2H)	2.04-1.97 (m, 2H)	2.01-1.97 (m, 2H)	1.26-1.24(1H, Hα) 1.99-1.97 (1H, Hβ)	1.30-1.24 (m, 2H)	2.04 – 1.97 (m,2H)
**10**	5.08 (t, J = 6.8, 1H)	3.31-3.28(dd,1H)	5.05(t,1H)	5.05(t,1H)	2.71 (t,1H)	3.57 (br.s, 1H)
**11**	-	-	-	-	-	-
**12**	1.63(s, 3H)	1.14(s, 3H)	1.61(s, 3H)	1.62(s, 3H)	1.18 (s, 3H)	1.18 (s, 3H)
**13**	1.57 (s, 3H)	1.09(s, 3H)	1.54 (s,3H)	1.55 (s,3H)	1.18 (s, 3H)	1.18 (s, 3H)
**14**	1.05 (s, 3H)	1.04(s, 3H)	1.07(s,3H)	1.04(s,3H)	0.81 (d, 3H)	1.15 (s, 3H)
**15**	1.60 (s, 3H)	1.58(s, 3H)	-	0.79(m, 3H)	-	3.65 (s, 2H)
**16 - OMe**	-	-	-	-	4.62 (s, 3H)	-

Table S2. ^{13}C NMR (100 MHz) spectroscopic data for α-bisabolol and compounds 2,3,4,5 and 6 (in $CDCl_3$).

Carbons	α -Bisabolol	^{13}C (ppm) (multiplicities, *J* in Hz)				
		2	3	4	5	6
1	26. 92 , CH2	27.2, CH2	24.9 , CH2	26. 4 , CH2	30.1, CH2	27.7, CH2
2	120.58, CH	120.5, CH	141.5 , CH	29.0 , CH2	129.8, CH	124.03, CH
3	134. 02, C	134.02, C	129.95, c	43.1, CH	133.8, C	135.9, C
4	31.03 , CH2	30.97, CH2	27.57, CH2	29.7 , CH2	26.4, CH2	31.9, CH2
5	23.30, CH2	23.25, CH2	22.59, CH2	25.7 , CH2	20.6, CH2	23.4, CH2
6	43.00, CH	42.35, CH	42.17, CH	47 , CH	39.8, CH	43, CH
7	74.24, C	74.41, C	74.22, C	74.4, C	42.8, CH	75.0, C
8	40.13, CH2	37.35, CH2	39.86, CH2	39.6, CH2	34.7, CH2	39.4, CH2
9	22.07, CH2	25.12, CH2	22.11, CH2	22.1, CH2	32.2, CH2	24.9, CH2
10	124.6, CH	79.12, CH	124.28, CH	124.4, CH	66.5, CH	74.3, CH
11	131.54 , C	73.3, C	131.95,C	132, C	62.8, C	70.9, C
12	25.66 , CH3	26.54, CH3	25.72, CH3	25.7, CH3	23.4, CH3	25.7, CH3
13	17.62 , CH3	26.54, CH3	17.69, CH3	17.6, CH3	23.4, CH3	25.7, CH3
14	23.16 , CH3	23.30, CH3	23.25, CH3	24, CH3	19.7, CH3	24.2, CH3
15	23.34 , CH3	23.39, CH3	171.61, C	17.1, CH3	165.3, C	63.7, CH2
16- OMe	-	-	-	-	56.2, CH3	

Table S3. The types of binding interactions of the most selective COX-2 inhibitors compound 4 and 5 in COX-1 compared to indomethacin a reference non-selective COX inhibitor.

Comp.	COX-1 inhibition (IC_{50} µM) / SI	2D pose against COX-1	Type of binding interactions
**4**	35.31±2.18 / 8.598	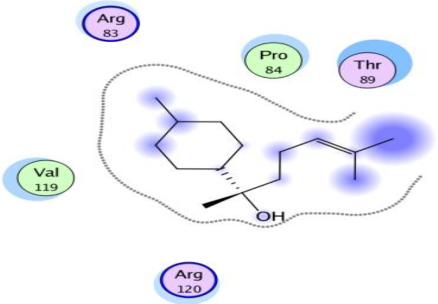	• Strong hydrophobic interaction with Thr89 and Pro84
**5**	16.83±1.04 / 6.711	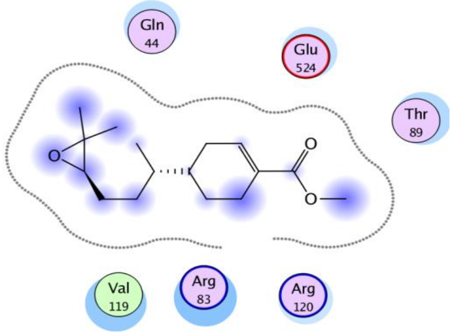	• Strong hydrophobic interaction with Va119 and Arg83

Indomethacin	0.15±0.01 / 0.318	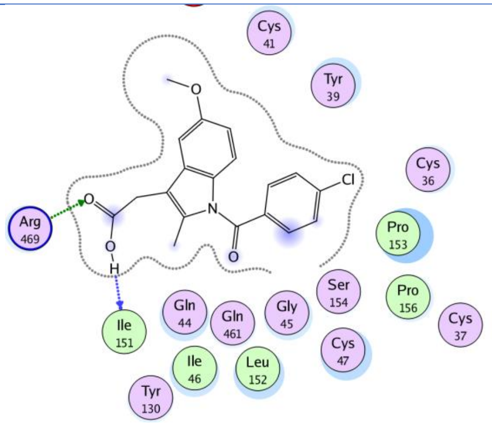	• H-bonds with Arg469 and Ile151<br>• Strong hydrophobic interaction with Pro153, Cys47, Leu152 and Tyr39

Table S4. The COX-2 inhibition (IC_{50}, μM, values are represented as means ± SD, n=3), selectivity index (SI)[a], docking scores [b] and type of binding interactions of bisabolol, its metabolites (2-6) and Celecoxib (reference compound).

Compound	COX-2 inhibition (IC_{50} μM) / SI	Binding energy (kcal/mol)[c] (docking score)	Type of binding interactions
**2**	17.34±0.66 / 4.093	-9.6	• Strong hydrophobic interaction with Val523, Ala527 and Val349
**3**	6.449±0.24 / 4.362	-10.6	• H-bond with Arg120<br>• Strong hydrophobic interaction with Val523 and Val349
**4**	4.107±0.15 / 8.598	-10.1	• H-bond with Val523<br>• Strong hydrophobic interaction with Val349, Leu352 and Ala527
**5**	2.508±0.09 / 6.711	-13.5	• H-bonds with Arg120 and Tyr355<br>• Strong hydrophobic interaction with Val523, Val349, Leu352 and Ala527
**6**	5.39±0.18 / 12.58	-10	• H-bonds with Ser119<br>• Strong hydrophobic interaction with Arg120, Tyr115, Met471, Pro86 and Lys83
**Bisabolol**	2.925±0.13/6.889	-9.9	• Strong hydrophobic interaction with Val523, Val349 and Leu352
**Celecoxib***	1.138±0.044 / 18.69	-15.2	• H-bonds with Arg120, Ser530 and Tyr355<br>• Strong hydrophobic interaction with Val349, Leu352 and Ala527

- *Reference COX-2 inhibitor.
- All data are presented as mean value ± SD for three independent experiments.
- [a] selectivity index (**SI**)= IC_{50} (μM) against COX-1 divided by IC_{50} (μM) against COX-2
- [b] More negative score refers to better capability of a molecule to dock with the target and make more desirable interactions.

Table S5. Results of acetylcholine esterase inhibitory assay represented by IC_{50} (µM, values are represented as means ± SD, n=3). The half-maximal inhibitory concentrations (IC_{50}) were calculated from the regression equations of the curves prepared in Microsoft Excel 2010 by plotting the % inhibition against sample concentrations.

**Compound**	**IC_{50} (µM)**
Bisabolol	> 100
2	12.94 ± 1.32
3	>100<br>(at 100 µM gave 20 % inhibition)
4	> 100
5	> 100
6	> 100

*Galantamine as positive control showed IC_{50} value of 1.50 ± 0.52.

Table S6. Acetyl choline esterase (AChE) inhibition (IC_{50}, µM), docking scores, and binding interactions of the tested isolated compounds and the reference compound (Galantamine).

compound	AChE inhibition (IC_{50} µM)	Binding energy (Kcal/mol)[c] (docking score)	Type of binding interactions
**2**	12.94 ± 1.32	-11.3	• H-bonds with Trp86 and Tyr337<br>• Strong hydrophobic interaction with Trp86 and Tyr337
**Bisabolol**	> 100	-5.3	• Strong hydrophobic interaction with Trp286
**Galantamine**	1.50 ± 0.52	-9.3	• H-bonds with Ser125 and Tyr337<br>• Arene-arene interactions with Trp86 and Tyr337<br>• Strong hydrophobic interaction with Trp86, Ser125 and Tyr337

- All data are presented as mean value ± SD for three independent experiments.
- Galantamine was used as reference AChE inhibitor compound.

Table S7. Calculated parameters of Lipinski's rule of five for α-bisabolol and metabolites 2-6.

Compound	Parameter						nVs[g]
	Log P[a]	TPSA[b]	MW[c]	nHBA[d]	nHBD[e]	nRB[f]	
**2**	2.27	60.69	256.38	3	3	5	0
**3**	2.86	57.53	252.35	3	2	5	0
**4**	3.99	20.23	224.38	1	1	4	0
**5**	3.51	38.83	266.38	3	0	6	0
**6**	1.44	80.92	272.38	4	4	6	0
α-bisabolol	3.76	20.23	222.37	1	1	4	0

[a]Calculated lipophilicity. [b]Total polar surface area. [c]Molecular weight. [d]Number of hydrogen bond acceptors. [e]Number of hydrogen bond donors. [f]Number of rotatable bonds. [g]Number of violations from Lipinski's rule of five.

Table S8. Predicted ADMET data for α-bisabolol and metabolites 2-6.

Compound	HIA%	Log S	CYP3A4 Inhibition	Carcinogenicity	hERG_inhibition
**2**	84.22	-3.39	Non-inhibitor	Non-carcinogenic	Low risk
**3**	94.51	-2.82	Non-inhibitor	Non-carcinogenic	Low risk
**4**	100	-3.97	Non-inhibitor	Non-carcinogenic	Low risk
**5**	98.59	-3.07	Non-inhibitor	Non-carcinogenic	Medium risk
**6**	72.70	-3.13	Inhibitor	Non-carcinogenic	Low risk
**α-bisabolol**	100	-4.37	Non-inhibitor	Non-carcinogenic	Low risk
